# Metabolic modeling and response surface analysis of an Escherichia coli strain engineered for shikimic acid production

**DOI:** 10.1186/s12918-018-0632-4

**Published:** 2018-11-12

**Authors:** Juan A. Martínez, Alberto Rodriguez, Fabian Moreno, Noemí Flores, Alvaro R. Lara, Octavio T. Ramírez, Guillermo Gosset, Francisco Bolivar

**Affiliations:** 1 Departamento de Ingeniería Celular y Biocatálisis, Instituto de Biotecnología, Universidad Nacional Autónoma de México (UNAM), Avenida Universidad 2001, Colonia Chamilpa, Cuernavaca, 62210 Morelos México; 2 Departamento de Ciencias Naturales, Universidad Autonoma Metropolitana (UAM), Vasco de Quiroga 4871, Colonia Santa Fe Cuajimalpa, Delegación Cuajimalpa de Morelos, México D.F., 05348 Mexico; 3 Departamento de Medicina Molecular y Bioprocesos, Instituto de Biotecnología, Universidad Nacional Autónoma de México, Avenida Universidad 2001, Colonia Chamilpa, Cuernavaca, 62210 Morelos México

**Keywords:** Metabolic modeling, Central carbon metabolism, Response surface analysis, Cybernetic modeling, Shikimic acid

## Abstract

**Background:**

Classic metabolic engineering strategies often induce significant flux imbalances to microbial metabolism, causing undesirable outcomes such as suboptimal conversion of substrates to products. Several mathematical frameworks have been developed to understand the physiological and metabolic state of production strains and to identify genetic modification targets for improved bioproduct formation. In this work, a modeling approach was applied to describe the physiological behavior and the metabolic fluxes of a shikimic acid overproducing *Escherichia coli* strain lacking the major glucose transport system, grown on complex media.

**Results:**

The obtained flux distributions indicate the presence of high fluxes through the pentose phosphate and Entner-Doudoroff pathways, which could limit the availability of erythrose-4-phosphate for shikimic acid production even with high flux redirection through the pentose phosphate pathway. In addition, highly active glyoxylate shunt fluxes and a pyruvate/acetate cycle are indicators of overflow glycolytic metabolism in the tested conditions. The analysis of the combined physiological and flux response surfaces, enabled zone allocation for different physiological outputs within variant substrate conditions. This information was then used for an improved fed-batch process designed to preserve the metabolic conditions that were found to enhance shikimic acid productivity. This resulted in a 40% increase in the shikimic acid titer (60 g/L) and 70% increase in volumetric productivity (2.45 gSA/L*h), while preserving yields, compared to the batch process.

**Conclusions:**

The combination of dynamic metabolic modeling and experimental parameter response surfaces was a successful approach to understand and predict the behavior of a shikimic acid producing strain under variable substrate concentrations. Response surfaces were useful for allocating different physiological behavior zones with different preferential product outcomes. Both model sets provided information that could be applied to enhance shikimic acid production on an engineered shikimic acid overproducing *Escherichia coli* strain.

**Electronic supplementary material:**

The online version of this article (10.1186/s12918-018-0632-4) contains supplementary material, which is available to authorized users.

## Background

The aromatic amino acid pathway (AAAP) branches from the central carbon metabolism (CCM) by the aldolic condensation of erythrose-4-phosphate (E4P) and phosphoenolpyruvate (PEP), being present in bacteria and plants. The AAAP is responsible for the production of aromatic amino acids and aromatic vitamins. As a consequence, it is an essential and highly regulated pathway [1, 2]. AAAP intermediates and final compounds play important roles in the pharmaceutical and food industries, either as raw materials, additives or final products [3–9]. Among them, shikimic acid (SA) can be used as an enantiomeric precursor to produce valuable biological molecules such as antipyretics, antioxidants, anticoagulants, antithrombotics, anti-inflammatories, analgesic agents, antibacterial, hormonal or antiviral compounds [8, 9]. SA was at first produced from the seed of the Chinese star anise plant *Illicium verum*, employing classic extraction processes with yields of only 30 *mg*/*Kg* approximately [10–12]. For this reason, over the past years, many studies concerning SA production have focused on recovery technologies, chemical synthesis methods and biotechnological production using different microorganisms [9, 13, 14]. The latter resulted in many genetically engineered strains that produce SA at laboratory and industrial scales with relatively high yields (between 40–50% mol/mol), but still far from the theoretical maximum (86% mol/mol) [2, 9, 13–15].

Although classic metabolic engineering (ME) allows flux redirection in a biochemical network into valuable compounds by genetic manipulation, it often induces significant flux imbalances to the CCM that may cause undesirable outcomes. These imbalances can disrupt precursor availability and energy balances, causing the accumulation of pathway intermediates and unwanted byproducts, reducing strain fitness and product yields [16]. These imbalances derive from alterations to the complex connectivity of biological information networks (genome, transcriptome, proteome, and metabolome) [17, 18]. Therefore, there is an increasing interest into a more global and detailed understanding of the metabolic and regulatory network changes imposed by different genetic modifications or process conditions in various production systems. In recent years, mathematical models, advances on informatics and the availability of big and more precise *omics* data sets have proved useful to resolve and clarify the complex network interactions and system characteristics [19–22].

To mathematically model metabolism, a metabolic network must be assembled with sufficient detail and curated from genomic data to be represented as a matrix of equations, including all available stoichiometric, thermodynamic and kinetic data. Given the complexity of microorganisms, the parameter sets required to describe the networks for genome-scale models are quite large and require informatically-intensive modeling approaches [23]. Most of the constructed metabolic models use mass balances and assume pseudo-steady state conditions to solve the highly undetermined linear equation systems and render a convex space, which contains all the possible solutions for the system. This solution space then must be narrowed with experimental data and some other assumptions to acquire a meaningful and useful solution [18, 19, 21, 24]. Different approaches have been developed to find the most meaningful solution, such as mechanism-based, interaction-based and the constraint-based methodologies. The latter, are the most commonly used for their capability to render useful flux distributions, even with relatively small amounts of information [20, 23]. Nevertheless, a challenging ground for models still exists for high-throughput data acquisition and interpretation when non-defined cultivation media and dynamic processes are used. The challenges and achievements within this field can be consulted elsewhere [20, 21, 25–31].

Regarding SA production with *E. coli*, few modeling studies could be found in the literature. Chen et al. (2011) [32] used a constraint-based analysis with flux balance analysis (FBA), assumed no growth and used SA as the objective function, to design modifications for the overproduction of AAAP intermediates. The model identified *aroF*, *tktA*, *ppsA* and *glf* genes as candidates for overexpression. As well, suggested the inactivation of *ldhA* and *ackA* genes to avoid carbon waste through lactate (LA) and acetate (AC) fluxes. These genes and nodes are in accordance to other reports on AAAP intermediate production [2, 5, 7, 14]. Nevertheless, this model also identified the non-evident *zwf* gene as critical for redirection of the carbon flux into E4P on the AAAP. Its overexpression resulted in an increase of 47% molar conversion of glucose (GLC) to aromatic intermediates [32, 33]. Similarly, Ahn et al. (2008) [34] constructed a model for maximizing SA production from GLC highlighting the importance of CCM genes like *tktA* and *zwf*, although growth or maintenance requirements were not considered. Rizk and Liao (2009) [35] used ensemble modeling, a mechanism-based approach, to identify *tktA* as the first-rate controlling step, founding that the *ppsA* gene can only augment production of aromatic intermediates when *tktA* is simultaneously overexpressed. There still are several challenges that must be addressed regarding model construction and implementation. For example, models are often limited by specific assumptions, defined conditions and are performed primarily under stationary constraints. Importantly, the assumption of stationary state provides only limited information on the dynamic properties of the system or network regulation. These limitations can result in some contradictions to real cell behavior under changing conditions, given by the existence of complex regulatory mechanisms modifying metabolic fluxes. New models and tools accounting for more complex solutions and on dynamic conditions, would result in a better understanding of cell behavior and produce new insights for strain and bioprocess design. On the other hand, for *E. coli* strains constructed for SA production, most of the work done has been focused on testing and improving expression platforms, genetic backgrounds, including the use of strains lacking the main phosphoglucotransferase transport (PTS), which lack catabolite repression and can redirect part of the carbon flux in to the production of aromatic compounds [2, 5, 7, 9, 33, 36], and culture strategies using traditional engineering approaches. Only few studies have focused on metabolic modeling to better understand and engineer SA overproduction at a more global level. Even less has been done on modeling production strains under complex media or on dynamic conditions, which are critical considerations for further process improvement. Here, a dynamic modeling approach of the physiological behavior and the dynamic metabolic flux distributions for an engineered *E. coli* strain is presented. The results were useful for strain behavior characterization and SA productivity enhancement on variable complex media compositions.

## Results

### Physiological characterization, parametrization and modeling of strain AR36 on variant substrate conditions

**Fig. 1:**
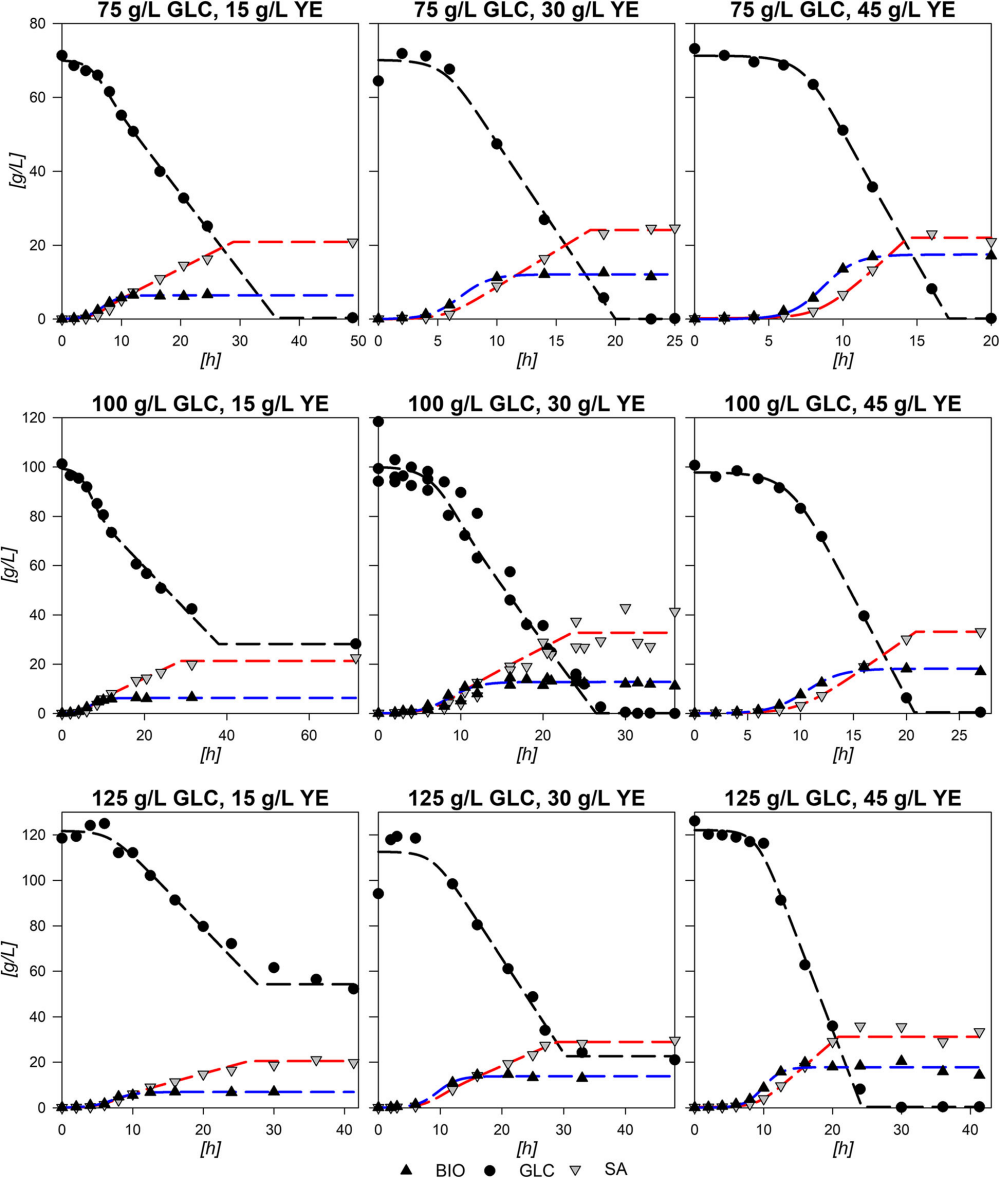
Physiological model approximation to each point of the experimental design

Figure 1 depicts the results from the 9 experimental design fermentations with the central point done by triplicate along with constructed physiological models (see “Methods” section). Central experimental condition (100:30 GLC:YE g/L) average parameters and deviations are summarized in Table 1. The standard deviations show relatively low values in accordance to experiments using yeast extract (YE) from three different batches. The largest standard deviations corresponded to final SA produced ([SA] _*f*_), final consumed GLC (*Δ*[GLC]) and the exponential consumption rate $\left (q_{s}^{exp}\right)$. Nevertheless, the averaged model depicts a fair agreement with data as can be observed in Fig. 1. The observed behavior and statistical data proved that the logistic models were suitable to describe and parametrize the consumption of GLC and production of SA in strain AR36, within the boundaries of the experimental design. Statistical validation and accuracy of the models are presented in Additional file 1.

**Table 1:** Average parameters and estimated standard deviations from the central point in the experimental design

	*X*_*max*_	*Δ*[GLC]	[SA] _*f*_	[AC] _*f*_	*μ*	$q_{glc}^{exp}$	$q_{sa}^{exp}$	$q_{glc}^{sta}$	$q_{sa}^{sta}$	*Y*_*ps*_	*Y*_*px*_	$q_{ac}^{exp}$	$q_{ac}^{sta}$
	[g/L]	[g/L]	[g/L]	[g/L]	[h ^−1^]	[g/Lh]	[g/Lh]	[g/Lh]	[g/Lh]	[g/g]	[g/g]	[g/Lh]	[g/Lh]
Mean	12.80	99.82	32.80	9.24	0.58	1.24	0.35	0.45	0.13	0.36	0.76	0.52	0.50
*σ* ^2^	0.77	4.77	8.13	0.95	0.12	0.48	0.09	0.19	0.08	0.03	0.16	0.19	0.19

**Fig. 2:**
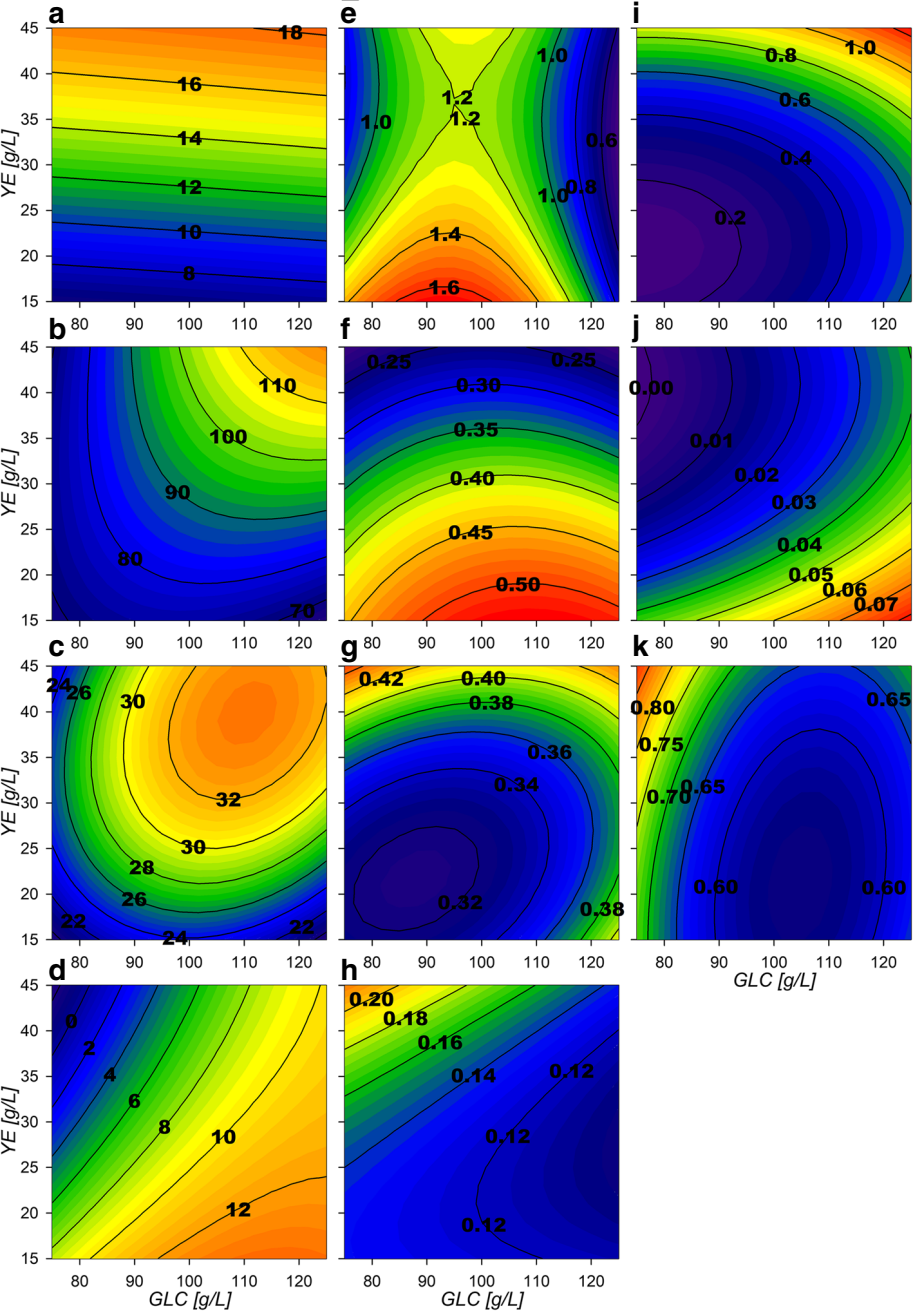
Response surface contour plots for the model estimated parameters. **a** Max biomass [g/L], **b** *Δ*[GLC] [g/L], **c** Final SA [g/L], **d** Final AC [g/L], **e**$q_{glc}^{exp}$ [g/Lh], **f**$q_{sa}^{exp}$ [g/Lh], **g**$q_{glc}^{sta}$ [g/Lh], **h**$q_{sa}^{sta}$ [g/Lh], **i**$q_{ac}^{exp}$ [g/Lh], **j**$q_{ac}^{sta}$ [g/Lh], **k** *μ*_*max*_ [h ^−1^]

With the parameters obtained, three-dimensional response surfaces were constructed (Fig. 2 and “Methods” section). Maximum biomass (*X*_*max*_) response surface (Fig. 2a) shows only small increases with higher GLC concentrations at similar amounts of YE. *Δ*[GLC] response surface (Fig. 2b) depicts that GLC consumption increases proportionally with higher starting GLC and YE concentrations. This is especially observed under high GLC concentrations, where at least ≈40 g/L of YE are required for complete exhaustion of more than ≈110 g/L of GLC. Regarding final SA concentration ([SA] _*f*_), surface morphology is similar to the consumed GLC surface, but exhibits a maximum critical point at 110:40 g/L GLC:YE initial concentrations (Fig. 2c). For kinetic parameters, GLC consumption rate at exponential phase $\left (q_{glc}^{exp}\right)$ shows a saddle type behavior on its response surface (Fig. 2e). This morphology is characterized by the existence of a maximum critical point for GLC and a simultaneous minimum for YE, found at 96 g/L and 37 g/L concentrations, respectively. These results suggest that cellular responses to GLC concentrations lower than ≈75 g/L (increasing consumption) or higher than ≈100 g/L (decreasing consumption), may be occurring in strain AR36. Surfaces also showed that $q_{glc}^{exp}$ highest values are found at lower concentrations of YE and GLC and the lowest rates under high concentrations of initial GLC. For the SA exponential production rate $\left (q_{sa}^{exp}\right)$ surface, a tendency to increase towards lower initial [YE] was found, with an up to 50% decrease when more than 40 g/L of YE are utilized (Fig. 2e and f). The growth rate (*μ*_*max*_) displays a minimum critical point on 105:21 g/L GLC:YE initial concentrations (Fig. 2k) with the highest values found towards lower [GLC] in combination with higher [YE]. Finally, the AC production rate $\left (q_{ac}^{exp}\right)$ shows a tendency to present higher values as [YE] and [GLC] increase (Fig. 2i) and could be responsible for reducing biomass and SA production rates as the AC highest rates were found above ≈40 g/L [YE] and ≈110 g/L GLC. In summary, all the specific rates at exponential phase suggest an allocation of rate maximization zones or quadrants on the experimental design as follows: at high [GLC] and high [YE] concentrations AC production is predominant, at low [GLC] and high [YE] concentrations biomass production is predominant, at high [GLC] but low [YE] concentrations SA production is predominant and finally at lower concentrations of both substrates a more balanced growth and production of all final products is to be found (Fig. 2).

At the stationary phase, a reduced metabolic activity on all consumption and production rates was observed. $q_{glc}^{sta}$ surface (Fig. 2g) tends to have larger values on higher initial concentration of substrate sources (GLC and YE). The SA stationary production rate $\left (q_{sa}^{sta}\right)$ surface (Fig. 2h) reveals a tendency to increase towards low GLC with high YE initial concentrations, showing an opposite behavior than $q_{ac}^{sta}$ (Fig. 2j). Their surface analysis helps to allocate predominant stationary phase output zones as follows. A SA production zone found above an imaginary diagonal line cutting the experimental design area from low initial concentrations of both substrate sources to high initial concentrations and a predominantly AC production zone found below this imaginary diagonal. It should be also noted that zone preferences on stationary phase are found on opposite sides respective to the allocated ones on the exponential phase. More so, SA specific production rates observed at higher initial [YE] and lower initial [GLC] conditions seem to have smaller variations between phases and AC specific production rates seem to vary less on low initial [YE] high initial [GLC] conditions.

**Fig. 3:**
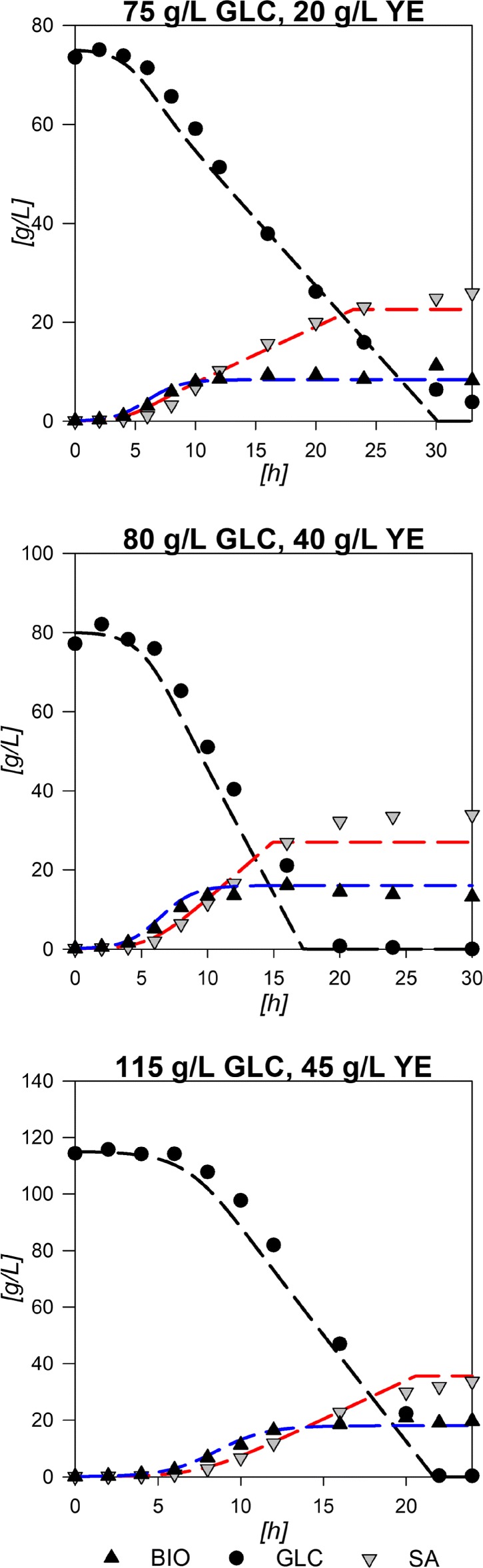
Physiological model predictions to three experimental validation experiments

The descriptive viability of the constructed response surfaces was validated by performing fermentations using three conditions not included in the experimental design (75:20, 80:40 and 115:45 GLC:YE initial conditions). Figure 3 shows the results for the logistic growth model and the consumption/production integrated models rendered with the surface calculated parameters: *X*_*max*_, *μ*, $q_{glc}^{exp}$, $q_{sa}^{exp}$, $q_{glc}^{sta},q_{sa}^{sta}$ and *SA*_*final*_ parameters. As it can be seen, all models follow the experimental data with good agreement. The largest observable deviation is on the maximum SA achievable on the 80:40 GLC:YE experiment, probably due to the contribution from YE to SA production. In addition, mathematical assessment of the validation was performed by a set of descriptive and inferential statistical comparisons between the modeled data and the experimental results. Error percentage was obtained by ratio of quadratic sums and presented values from 0.14 to 0.91 for biomass comparisons, from 0.04 to 0.08 for GLC and from 0.04 to 0.26 for SA, suggesting a relatively small deviation between experimental and modeled data along the fermentation. *R*^2^ values were found between 0.96 and 0.99 for all three curves with percentile deviations from the expected slope (SPD) values lower than 1% and *p*-values below 0.05. These statistical values and the depicted models from Fig. 3 show that the models constructed by the surface predicted parameters can render comprehensively good representations for biomass, GLC and SA for initial conditions within the range of the experimental design. Surface predicted parameters were also validated by comparison with the ones calculated directly from experimental data. Table 2 shows the experimental and modeled parameters for all validation experiments and the average error calculated. The individual experimental error between predicted and calculated parameters can be found on Additional file 1. [AC]_0_ presented the highest error, probably because in experiments with high initial [GLC] and [YE] no AC was produced on stationary phase and surfaces constructed with the polynomial equation cannot properly render these behavior values. $q_{glc}^{exp}$ and *Y*_*p*/*s*_ also had relatively high errors above 10%. This may be related to the contribution of YE since it does not only contains the aromatic amino acids needed for growth, but also other amino acids and some carbohydrates that could contribute to some of the previously discussed effects. However, the two-tailed *t*-student test for the comparison of experimental and modeled parameters validated all parameters as similar, with *p*-values over 0.05. This means that the predicted values can be used within reason to compare and study the behavior of AR36 under the limits of the experimental design and that the constructed surfaces can be used to obtain further insights on cell behavior. Parameter data and statistical values for all experiments can be found on Additional file 1.

**Table 2:** Experimental vs Response surface predictions and statistical values calculated from fermentations used for model validation

initial GLC:YE g/L		75:20		80:40		115:45		%Error
		Exp.	Model	Exp.	Model	Exp.	Model	Average
Max biomass	[g/L]	9.30	8.40	14.30	16.03	19.76	18.05	7.18
Consumed GLC	[g/L]	70.03	70.70	80.11	78.19	115.76	113.84	1.18
Final SA	[g/L]	24.62	22.13	33.19	26.62	31.84	32.96	7.88
Final AC	[g/L]	5.25	6.45	0.00	0.85	4.90	8.73	35.70
*μ*_*max*_	[h ^−1^]	0.62	0.73	0.78	0.77	0.55	0.65	8.56
$q_{glc}^{exp}$	[g/Lh]	0.89	1.17	0.93	0.97	0.73	0.99	17.03
$q_{sa}^{exp}$	[g/Lh]	0.36	0.42	0.32	0.28	0.24	0.24	7.35
$q_{glc}^{sta}$	[g/Lh]	0.33	0.32	0.38	0.39	0.41	0.42	2.07
$q_{sa}^{sta}$	[g/Lh]	0.15	0.13	0.18	0.18	0.15	0.15	2.88
*Y*_*ps*_	[g/g]	0.41	0.36	0.35	0.29	0.34	0.23	14.49
*Y*_*px*_	[g/g]	0.59	0.59	0.42	0.38	0.44	0.38	5.68

### Dynamic Flux Metabolic Modeling of AR36 strain on variant substrate conditions

**Fig. 4:**
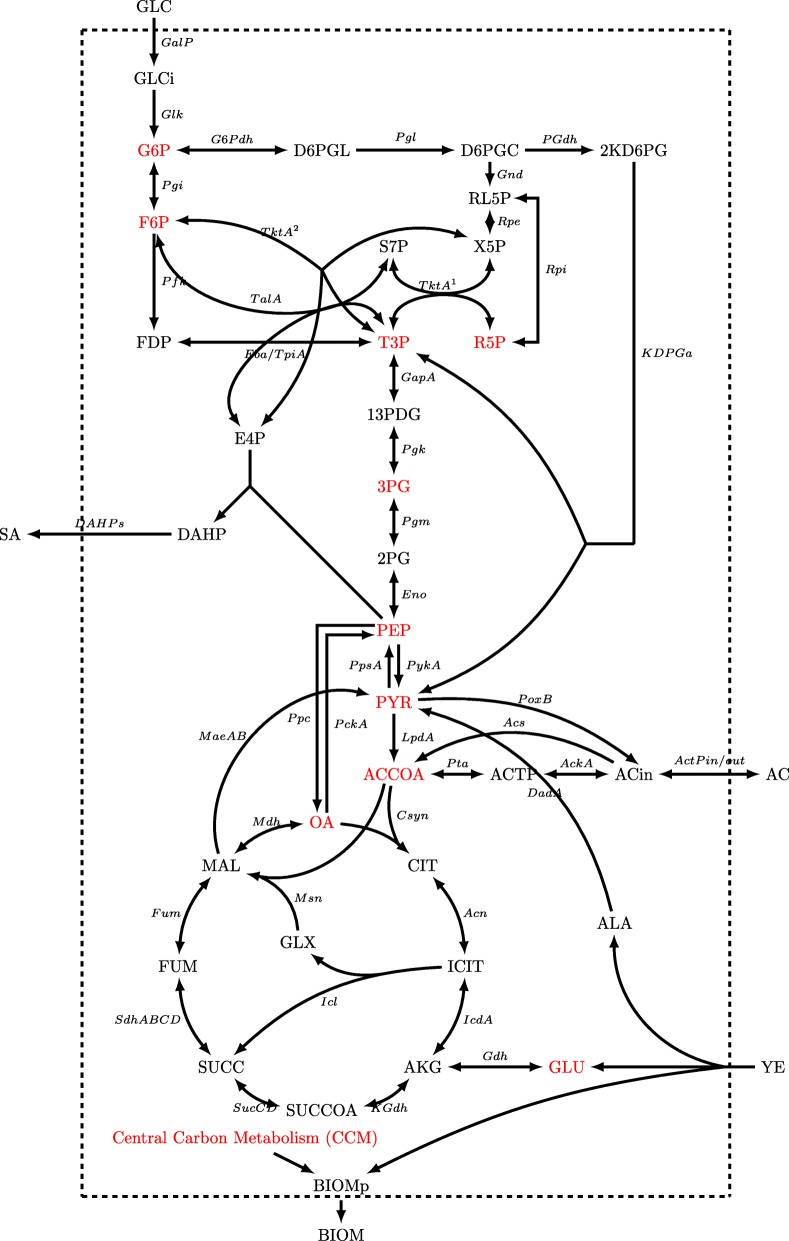
Central carbon metabolism constructed metabolic network for dynamic metabolic flux models. Metabolites on red refer to the CCM intermediaries used to produce biomass precursor (BIOMp)

**Fig. 5:**
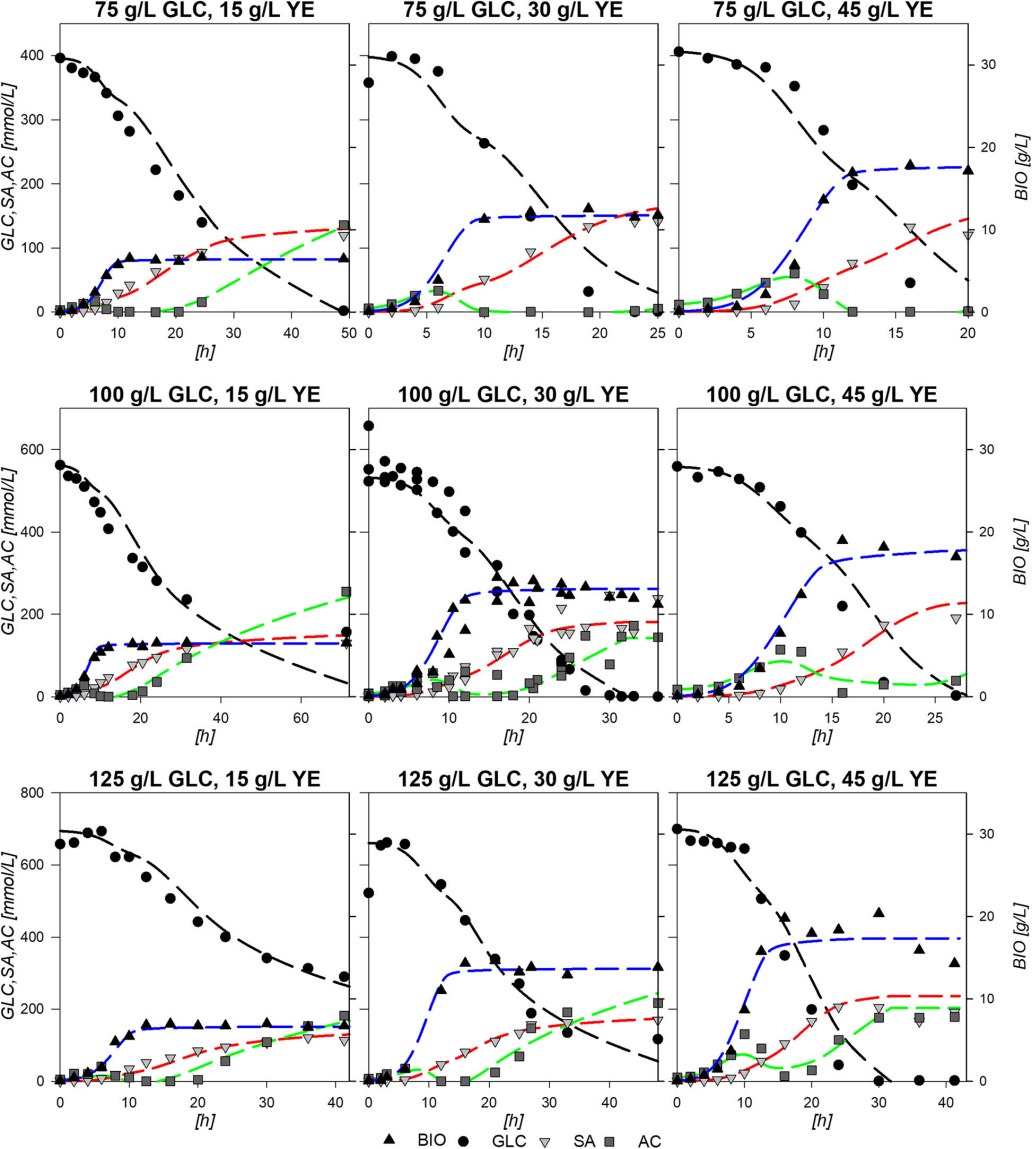
External metabolite model results from internal model flux computations for each point of the experimental design

To get further insight into these different output zoned behaviors, dynamic flux models were constructed. It is evident that AR36 strain regulation showed no linear borders and contributions between predominant outcomes. Since data on internal fluxes, constraints on regulation or other kinetic data were not available, a cybernetic modeling approach was used (See “Methods” section). The simplified metabolic network used for the metabolic models is depicted on Fig. 4, names of reactions will be referred onward as indicated in this figure. The complete description of the reactions can be found on Additional file 2. Calculations over this network resulted in dynamic models which followed the extracellular experimental data points with good agreement in all cases, as shown in Fig. 5. It should be noted that in this case, even the behavior of AC could be accurately described. The main characteristics of the common AC profile for fermentations start with an AC production section until approximately the middle of the exponential growth phase, only to be completely consumed in almost all fermentations towards the end of growth. A second AC production section starts at the stationary phase on all experiments except for the ones with 75:30 and 75:45 g/L GLC:YE initial conditions. For the models, values between 0.15 to 2.28% error were found for biomass approximations, from 0.56 to 2.3% error for GLC, from 0.25 to 4.19% error for SA profiles and 0.35 to 10.53% error for AC models in comparison to experimental data. All *R*^2^ from Pearson linear regressions were found to be above 0.9 and their significance *p*-values were all found to be below 0.05. Regarding SPD, the highest values were found for GLC and AC profiles. Specifically, a 21% deviation was found for GLC in the 100:15 condition, where the model presents higher GLC consumption at the last part of the fermentation compared to the experimental values. As it can be seen on Fig. 5, on this particular condition model almost exhaust GLC but experiment presents a final GLC value of 156 mM, which means that model over estimates GLC consumption on this particular condition. In comparison, in all other cases, models tend to underestimate the consumption rate on the last part of the fermentations with values ranging from 1.07 to 19% SPD, where the highest deviations corresponded to fermentations with greater initial YE concentrations. On that regard, on 75:15 g/L the previously observed underestimation of consumption rates at late stationary phase for the other experimental design conditions could mean that GLC may be exhausted on a time prior to the model estimations. Regarding AC, SPD deviations ranged from 1.31 to 9% in all cases, except for 100:30 and 100:45 where values where 21 and 42% respectively. For the 100:45 condition, this overestimation is due to the error in the AC peak found on mid exponential phase and to the lack of AC production in stationary phase. These large deviations can be explained by taking into account that YE contribution was simplified to only consider it as a biomass precursor and to provide simultaneously glutamate (GLU), alanine (ALA) and aromatic amino acids (taken as one individual metabolite). Nevertheless, the mathematical values along with the observed model behaviors depicted on Fig. 5 suggest that the models constructed are viable approximations to the observed strain behavior under the experimental conditions. All statistical data on the dynamic flux models are available in Additional file 1.

Calculated fluxes were normalized against GLC consumption derived fluxes for their analysis and surface construction on three different fermentation stages: initial exponential (IEx), mid exponential (MEx) and mid stationary (MST). IEx and MEx presented highly similar behaviors, so their description is similar and only IEx surfaces were addressed. However all surfaces and contour plots for all reactions and time sets can be found in Additional file 3.

#### Central carbon metabolism flux distribution behavior during growth

**Fig. 6:**
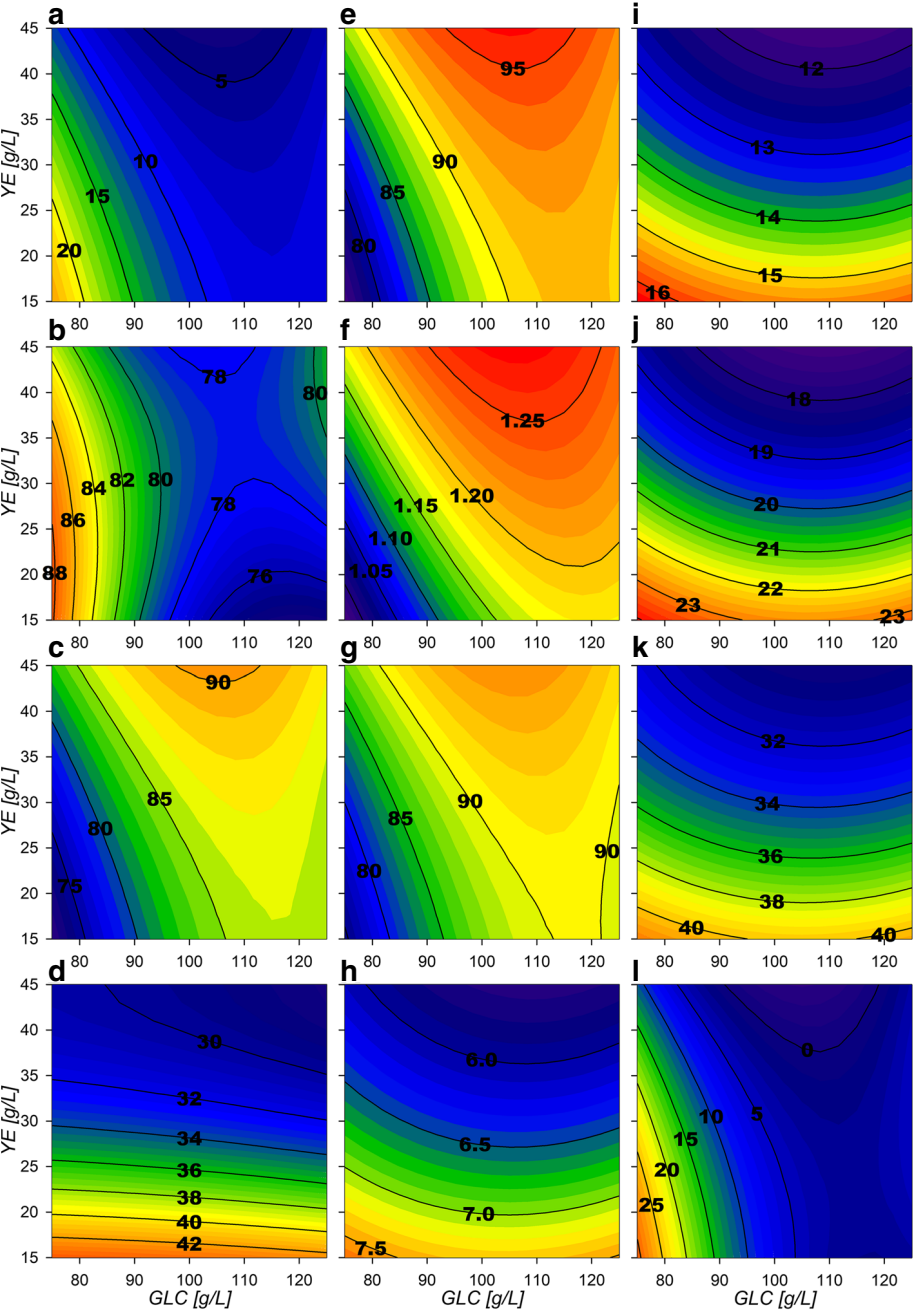
Response surface contour plots for the estimated internal fluxes at IEx (% Flux relative to GLC consumption). **a** Pgi, **b** GapA, **c** PykA, **d** LpdA, **e** G6Pdh, **f** Gnd, **g** PGdh, **h** TktA 1, **i** TktA 2, **j** DAHPs, **k** PckA, **l** Ppc

**Fig. 7:**
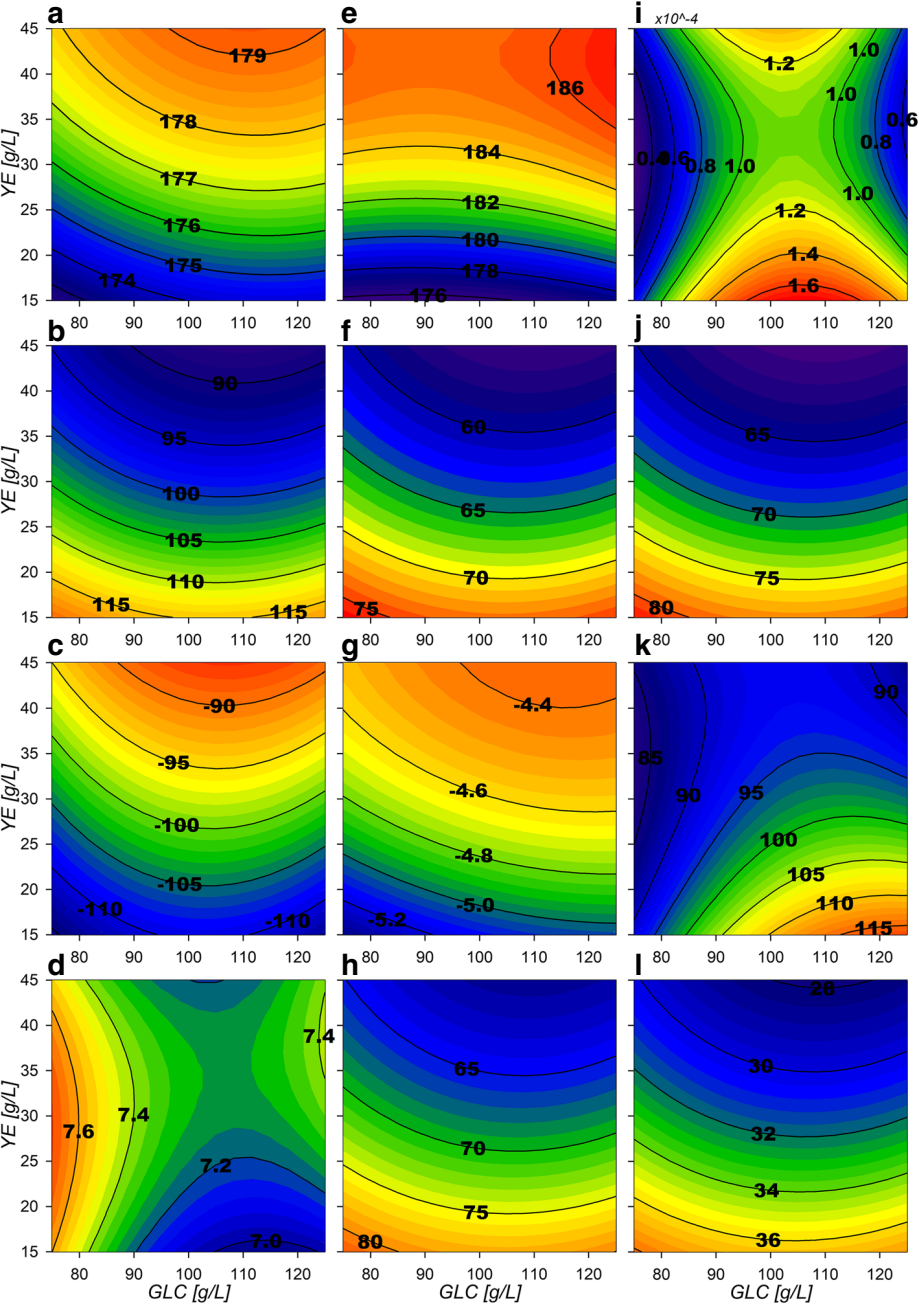
Response surface contour plots for the estimated internal fluxes at IEx pt2 (% Flux relative to GLC consumption). **a** ActPout, **b** ActPin, **c** AckA, **d** Acs, **e** PoxB, **f** Csyn, **g** IcdA, **h** Icl, **i** KGdh, **j** SdhABCD, **k** Mdh, **l** MaeB

Selected CCM genes related to IEx flux response surfaces are presented in Figs. 6 and 7. Glycolytic surfaces under growth conditions show the same morphology from glucose-6-phosphate (G6P) to PEP reactions, a saddle critical point with greater relative fluxes at low GLC initial conditions. Pgi (Fig. 6a), Pfk and Fba flux surfaces describe the same morphological behavior as GalP and Glk flux surfaces, but have smaller values than expected, accounting for only ≈6 to 25% of the flux relative to Glk (Additional file 3). This means that the majority of the flux is predicted to enter the oxidative reactions of the pentose phosphate pathway (PPP) by G6Pdh coded by *zwf* and the 6-phosphogluconolactonase (Pgl). G6Pdh presents relative flux values from ≈75 to 95% (Fig. 6e) and its morphology presents the inverse features than Pgi, which presents greater relative flux values at higher [GLC] and [YE] initial conditions. High relative flux values of 75 to 88% were found for reactions from glyceraldehyde-3-phosphate dehydrogenase-A (GapA) (Fig. 6b) and the following Embden-Meyerhoff-Parnas pathway (EMP) reactions towards PEP. Their surfaces have a similar morphological behavior as the upstream glycolytic fluxes but with higher values, suggesting that even with small Pgi flux distributions, high total conversion rates of glucose to PEP can still be present. Also, the flux distributions calculated on the 6-phosphogluconate dehydrogenase(Gnd)/phosphogluconate dehydratase (PGdh) node, showed relative flux values from ≈75 to 94% going through the Entner-Doudoroff pathway (EDP) (Fig. 6f and g respectively). This suggests that most of the carbon flux going through to the oxidative PPP is redirected towards glycerol-3-phosphate (G3P) and pyruvate (PYR).

Pgl, Gnd, PGdh and 2-Keto-3-deoxy-6-phosphogluconate aldolase (KDPGa) flux surfaces show the same morphology as G6Pdh, a marked tendency of maximization towards higher initial [YE] (Fig. 6e–g). In these conditions, the maximum biomass production zone was found that is in agreement to the previously observed high oxidative PPP flux distribution. They also show the inverse morphology than the EMP fluxes surfaces, as they are expected to compete for carbon skeletons. Regarding the non-oxidative reactions of the PPP, transketolase I (TktA) and transaldolase (Tal) flux surfaces (Fig. 6h) display low relative flux values (≈6 to 8%). Their surface morphology shows a tendency to increase towards lower initial [YE]. TktA surface representing the fraction of carbon being redirected from EMP towards the non-oxidative branch of PPP, shows values ranging from ≈ 12 to 16% (Fig. 6i). These non-oxidative branch reactions are responsible for the E4P formation and present combined flux values from ≈ 18 to 24%, which matches the predicted flux being redirected toward SA production by the 2-dehydro-3-deoxyphosphoheptonate aldolase (DAHPs) during growth (Fig. 6j). An interesting consequence is that up to ≈72 to 90% of flux modeled is going through the pyruvate kinase II (PykA) related reaction (Figure 6c), meaning that the majority of PEP is probably being converted to PYR. Phosphoenolpyruvate carboxylase (Ppc) surface shows the same morphology described by the glycolytic genes (Fig. 6l), indicating that on low [GLC] and low [YE] conditions, glycolytic metabolism is favored. Meanwhile, Phosphoenolpyruvate carboxykinase (PckA) (Fig. 6k) presents a tendency to increase flux towards low initial [YE] conditions. PckA and Ppc fluxes presented values ranging from ≈31 to 41% and ≈0 to 30%, respectively. Their simultaneous flux, suggests the existence of an ATP consuming futile cycle.

The high inflow to PYR is probably caused by the high EDP and PykA relative fluxes and increased even further by a high malic enzyme carbon reincorporation from the Tricarboxylic Acid Cycle (TCA), accounting for ≈28–38% from the NADPH dependent enzyme (MaeB) (Fig. 7l) and ≈0-30% from the NADH dependent (MaeA). In the model, PYR can also be produced from YE-derived ALA conversion by alanine D-amino acid dehydrogenase (DadA) reaction. On the other hand, for PYR conversion to acetyl coenzyme-A (ACCOA), the reaction was attributed to pyruvate dehydrogenase (LpdA). This reaction showed relatively small values, from ≈28–42% of relative flux (Fig. 6d) compared to pyruvate oxidase (PoxB), which presented fluxes towards AC calculated to be between ≈176–186% during growth phase (Fig. 7e). It is noticeable that for AC production, no constraint was imposed for flux preference on either acetate kinase (AckA), acetyl-CoA synthetase (Acs) and PoxB reactions and the model renders consumption over the reversible (AckA) since it is energetically favorable compared to Acs (Fig. 7c–e). Surfaces for extracellular AC export and import fluxes for AR36 (ActPout and ActPin) show greater export rates with higher initial YE concentrations. This could be attributed to the introduction of carbon to the CCM through ALA and GLU consumption (Fig. 7a and b), but can also be extended to other YE-derived amino acids catabolized through TCA not included on the model. On the other hand, the import of AC presents a maximization tendency towards low initial [YE] with relative flux values between ≈ 90 to 115%. To clarify the node distribution around PoxB, an AR36 *ΔpoxB* strain was constructed and cultured under high [GLC] and high [YE], conditions that maximize AC production according to the response surfaces. Interestingly, the initial AC concentration peak observed in all previous experiments was not detected in this case with the mutant strain (Additional file 4). Furthermore, the final AC concentration was significantly lower compared to AR36 on similar fermentation conditions. This suggest that PoxB could be indeed the main contributor to AC production in the AR36 PTS ^−^ strain [2, 33, 36–38]. The AR36 *ΔpoxB* cultures also showed lower growth rates (0.21 h ^−1^) and lower exponential GLC consumption rates (0.61 g/gh) (Additional file 4). This may indicate that its inactivation could be causing PYR accumulation and less ATP generation via the electron-transfer chain [39].

Regarding TCA behavior, the *glta* coded citrate synthase (Csyn) reaction presents relative flux values from ≈56 to 75% (Fig. 7f). As expected aconitase (Acn) reaction (Additional file 3) presents the same behavior as the Csyn reaction and both present the inverse morphological features compared to the AC producing surfaces. Conversely, the following reaction by isocitrate dehydrogenase (IcdA) seems to not be sending carbon flux down TCA. On the contrary, its reversible reaction is found, transporting the small excess of GLU derived from YE consumption towards isocitrate (ICIT) (Fig. 7g). The isocitrate lyase (Icl) and malate synthase (Msn) from this pathway, having relative fluxes values accounting from ≈60 to 82% of relative flux (Fig. 7h), and present the same surface morphology as the TCA carbon uptake Csyn flux surface. On the other hand, 2-ketoglutarate dehydrogenase (KGdh) and succinyl-CoA synthetase (SucCD) complexes seem to be catalyzing very small amounts of flux towards succinate (SUC) (Fig. 7i). Calculations for the glutamate dehydrogenase (Gdh) show relative fluxes between ≈3.1 to 3.7%, suggesting only small input by [YE] components into TCA and apparently processed mainly by IcdA. This means that SUC, is mostly produced by the glyoxylate shunt pathway (GSP) and subsequently catalyzed to malate (MAL) by the succinate dehydrogenase complex (SdhABCD) (Fig. 7j) and the fumarase (Fum). Their surfaces share the morphological characteristics of the Csyn and the GSP surfaces. In consequence, the malate dehydrogenase (Mdh) reaction exhibits higher relative flux values, from ≈85 to 1125% (Fig. 7k) as it also assimilates ACCOA carbon derived from the Msn reaction on GSP.

Surface morphologies suggest the allocation of different predominant extracellular production zones at the exponential growth phase. A SA production predominance flux zone is found at low initial [YE] and as the initial [GLC] diminishes a more balanced production towards biomass and SA is found. This follows up to the predominant region for biomass found at low initial [GLC] conditions. Finally, a clear AC predominant production zone is found at high initial [GLC] and [YE].

#### Central carbon metabolism flux distribution behavior after the growth phase

**Fig. 8:**
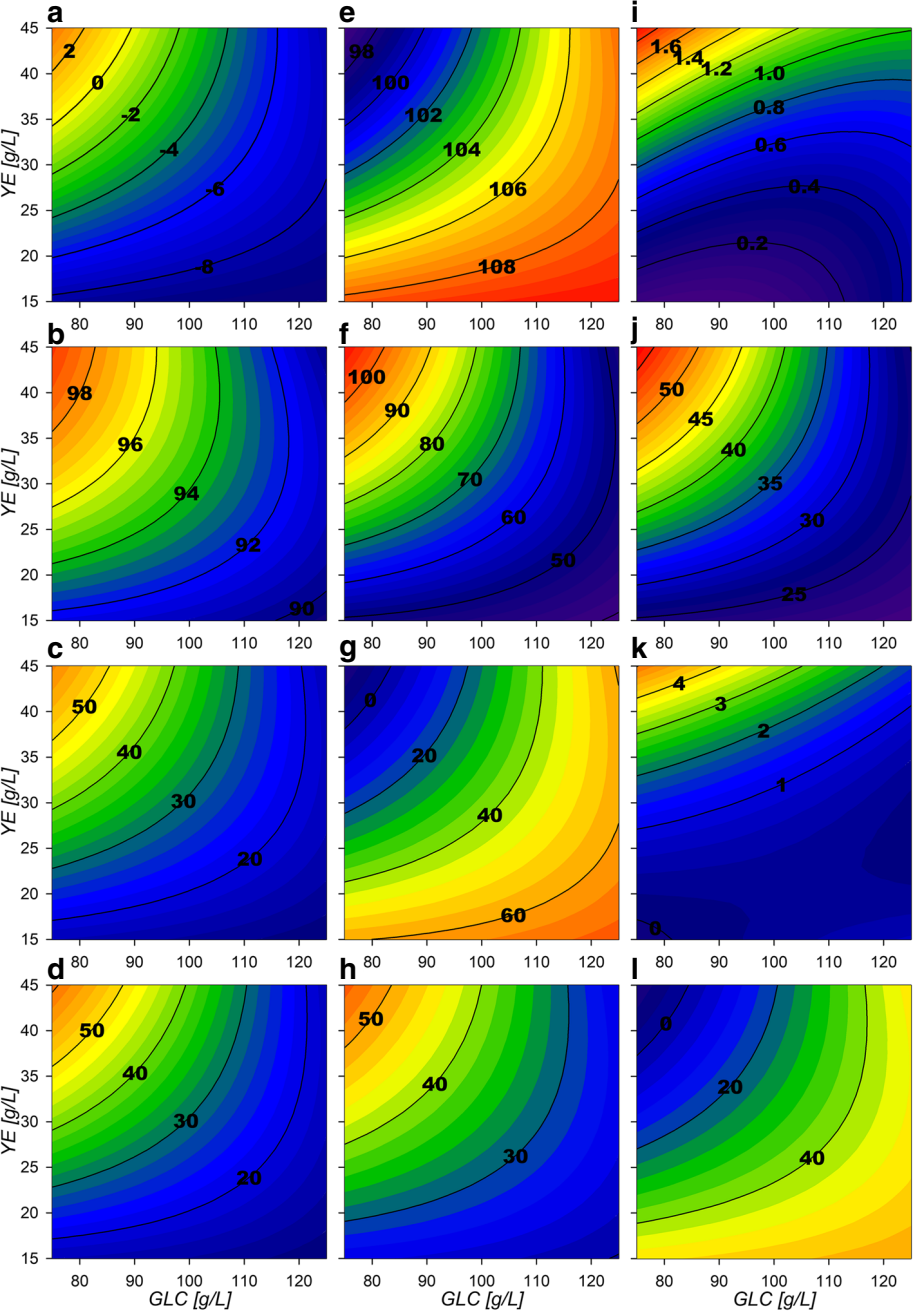
Response surface contour plots for the estimated internal fluxes at MSt (% Flux relative to GLC consumption). **a** Pgi, **b** GapA, **c** PykA, **d** LpdA, **e** G6Pdh, **f** Gnd, **g** PGdh, **h** TktA 1, **i** TktA 2, **j** DAHPs, **k** PckA, **l** Ppc

As in IEx and MEx phases, a high flux distribution towards the PPP was found for the MST phase, presenting about 97–108% relative flux through G6Pdh and Pgl (Fig. 8e). Both oxidative PPP reactions presented a marked minimization morphology towards low initial [GLC] and high [YE] initial concentrations and depicting decreasing ring like border lines. In contrast to the exponential phase, Gnd reaction shows values of relative flux from ≈40 to 100% (Fig. 8f), whereas its competing PGdh accounts for ≈0–70% of relative flux towards EDP. Both morphologies present inverse behavioral surface features, as observed on Fig. 8e and f. Interestingly, PGdh surface has the same max region on its morphology as G6Pdh surface, suggesting that excess flux could be still being processed by the EDP. Gnd surface morphology depicts a ringed type tendency with greater values towards initially low [GLC] and high [YE] experimental conditions (up-left corner of the experimental design). As expected, TktA reaction towards the non-oxidative PPP (Fig. 8h) and Tal reactions, exhibit the same behavior as the flux through Gnd surface and become greater contributors to the production of E4P and F6P. The latter enters the glycolytic EMP and it is mostly redirected down the glycolytic pathway through Pfk and Fba reactions with a relative fluxes of ≈10–55%. They also show a ringed surface morphology that tends to maximize towards low [GLC], high [YE] initial conditions. In fact, this morphology was observed through all following glycolytic reactions towards PEP formation and present values from ≈90 to 99% (Fig. 8b). Interestingly, the model renders a small (≈0–9%) unexpected flux distribution of carbon through Pgi (Fig. 8a), redirecting F6P to G6P, just to be consumed again through G6Pdf on almost all the experimental design area. Consequently, Pgi presents only a small flux in the standard G6P to F6P direction at low [GLC] and high [YE] initial conditions corner, representing only ≈0–2%.

**Fig. 9:**
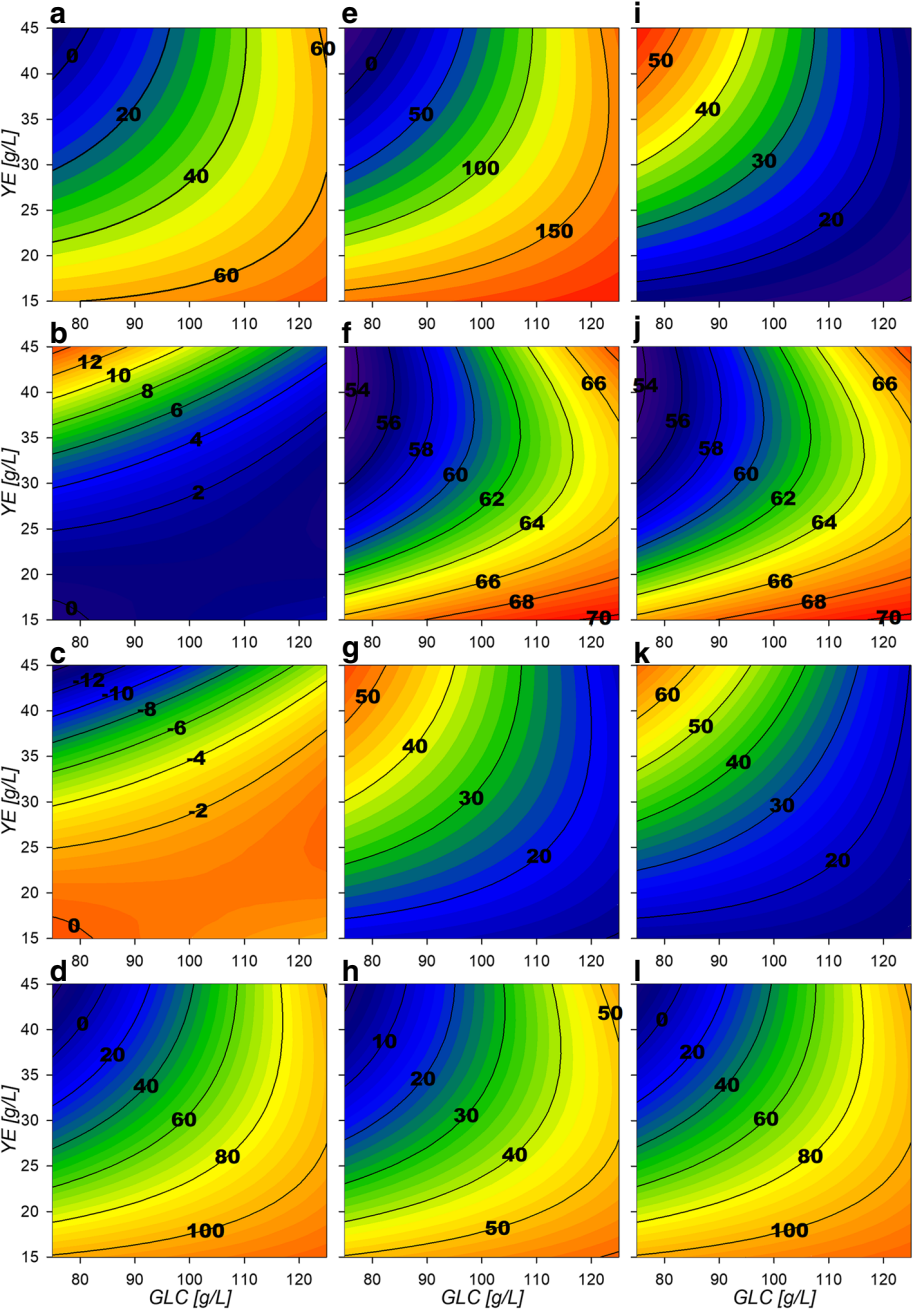
Response surface contour plots for the estimated internal fluxes at MSt pt2 (% Flux relative to GLC consumption). **a** ActPout, **b** ActPin, **c** AckA, **d** Acs, **e** PoxB, **f** Csyn, **g** IcdA, **h** Icl, **i** KGdh, **j** SdhABCD, **k** Mdh, **l** MaeB

On the PEP node reactions, the phosphoenolpyruvate synthase (PpsA) reaction is non-existent, suggesting no gluconeogenic flux from PYR towards PEP is obtained in any condition. To the same extent, PckA reaction catalyzing carbon flux from oxaloacetate (OA) towards PEP is quite low with relative fluxes between ≈0–5% and only being present under high [YE] initial conditions (Fig. 8k). On the other hand, its counterpart reaction Ppc shows ≈0–50% relative flux values, depicting inverse surface morphology features compared to the PckA surface, maximizing towards low initial [YE] conditions and towards higher [GLC] and also with a ringed behavior (Fig. 8l). Subsequently, PEP consumption by PykA and DAHPs presented the same ringed maximization tendency towards low initial [GLC] and high [YE] conditions as observed on the glycolytic surfaces. In contrast to the observations made under growth conditions, both of these fluxes have an equilibrated flux distribution along their surfaces with values between ranging between ≈15–55% (Fig. 8c and j), probably because of the higher E4P production on the PPP. These reactions compete with the AC production reactions, in specific with PoxB, which exhibits higher relative flux values as higher initial [GLC] and lower initial [YE] conditions are set on fermentation (Fig. 9e). Therefore, presenting the inverse surface morphological behavior compared to PykA and DAHPs surfaces. This may be explained as on higher initial concentrations of [YE] more biomass is produced and therefore more [GLC] is consumed by the start of stationary phase, which means that less [GLC] is expected at this time and therefore, metabolic overflow is expected to be lower. The export modeled transport reaction follows PoxB flux surface behavior (Fig. 9a) as it is observed to be again the main AC producing reaction. On the other hand, import reaction presents almost ≈0 flux values on low initial [YE], with consumption of extracellular AC only towards the low [GLC] with high [YE] initial conditions corner (Fig. 9b). These results suggest that the futile carbon cycling on the AC pathways is found on this stage only under high [YE] conditions [2, 33, 36–38, 40].

Regarding TCA and GSP, their surfaces exhibit ringed type maximization or minimization morphologies towards the upper left corner of the experimental design. Specifically, Csyn and Acn reactions present ≈54 to 70% relative fluxes with the minimization morphology behavior towards low [GLC] and high [YE] initial experimental conditions corner (Fig. 9f). The GSP fluxes follow the same morphological behavior along the experimental design (Fig. 9h).

In contrast to the growth phase, IcdA, KGdh and SucCD reactions present flux directions towards GLC oxidation on all the experimental area, with relative fluxes between ≈10 to 55% (Fig. 9g, i, j), and with its surface morphology maximizing towards low [GLC], high [YE] initial conditions. SdhABCD and Fum follow the same behavior of the Csyn surface. In addition, as higher fluxes are pulled through the TCA, higher is the MaeB reaction flux which competes with Mdh (Fig. 9l and k respectively). Their surface morphology suggest carbon skeleton recycling from PYR, flowing through AC pathways and into TCA to PYR again. This behavior is found under high [GLC] substrate conditions with higher metabolic flux saturation zones.

The dynamic cybernetic model on this phase showed two predominant production zones. The observations made by the dynamic flux models allocate a SA production zone above an imaginary diagonal line, cutting the experimental design area from low to high initial substrate concentration, and a predominantly AC production zone was found below the same imaginary diagonal, in accordance to the physiological models surface allocations.

### **Bioprocess design for SA productivity enhancement on strain AR36**

**Fig. 10:**
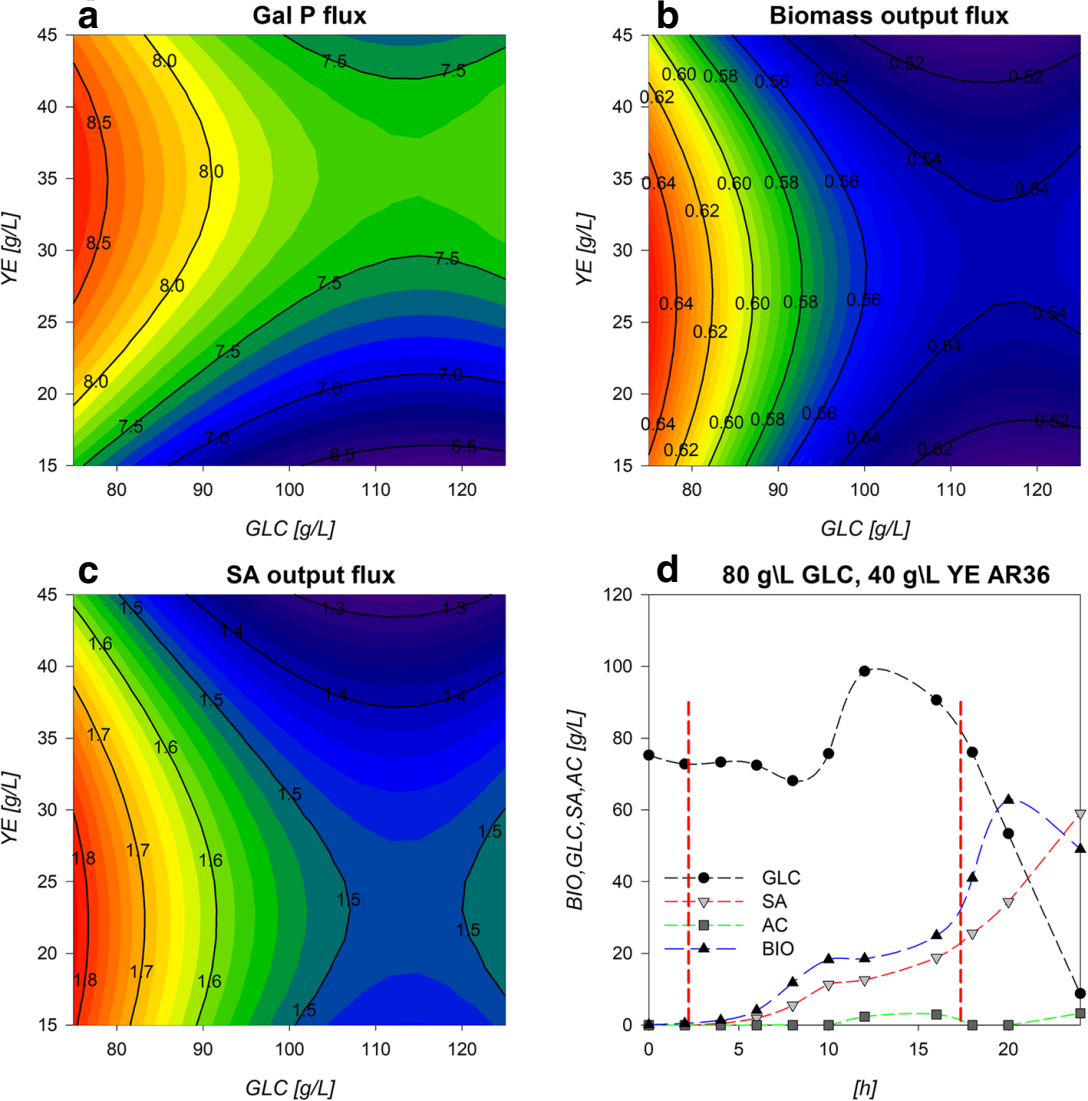
**a** GalP flux surface [mM/h], **b** Biomass flux surface [mM/h], **c** DAHPs flux surface [mM/h], **d** Fed-batch reactor fermentation maintaining initial operation concentration parameters. 80 g/L GLC and 40 g/L YE

To assess the utility of the previously described models and considerations towards SA production enhancement, a fed-batch fermentation process was performed with initial conditions of 80 g/L and 40 g/L initial [GLC] and [YE]. Surfaces revealed higher growth rates have been found under high [YE] and low [GLC] conditions (Fig. 2k). Under these conditions there are also zones with lower final AC production and mid range SA production, and high biomass production (Fig. 2c, d and a) for the final metabolic outputs. Although maximum SA titer was found near 110:40 GLC:YE condition in batch mode, this also results in higher AC production and lower consumption rates and yields on the stationary phase which result on incomplete substrate exhaustion (Fig. 2c, d, h and g). In contrast, higher SA production, higher GLC consumption and lower AC production rates on stationary phase are found (Fig. 2h, g and j) near 80:40 GLC:YE conditions. Therefore, with these initial conditions, biomass n with high rates are expected on the exponential phase without compromising stationary phase SA production and GLC consumption capabilities. This is supported also by the flux surface analysis, on the 80:40 GLC:YE initial conditions on stationary phase where relative fluxes are found to enhance SA acid production and GLC consumption, marked by the maximizing tendency for the reactions Pgi, GapA, PykA, Gnd,TktA, DAHPs and PckA (Fig. 8a, b, c, f, h, i, j and k). Also, in these selected conditions, lower values for relative fluxes were found for the reactions G6Pdh, PGdh and Ppc (Fig. 8e, g and l). This suggests that under the selected conditions less flux is sent towards the EDP and more is redirected to E4P through Gnd and TktA on the stationary phase. In fact the data indicate an ≈ 50% carbon flux redirection towards SA and the rest through lower glycolytic reactions by PykA and LpdA. Furthermore, consumption of AC fluxes will be maximized under this phase as seen on the relative flux surfaces for ActPin and AckA and lower AC production by PoxB (Fig. 9b, c and e). Therefore, higher fluxes are expected for GLC consumption and SA production, along with low AC production for the stationary phase. These conditions where then chosen even with the trade off with the exponential phase which presents higher GLC consumption flux rates for GalP (Fig. 10a) with high biomass production fluxes (Fig. 10b) and where SA production is not maximized. SA production on stationary phase presents higher values for DAHPs relative fluxes at lower initial [GLC] concentrations (Fig. 6j). Nevertheless, flux rates for SA production reactions (DAHPs) presented medium range values within the experimental region (Fig. 10c).

As mentioned, fed-batch operation was designed to favor the biomass preferential production during growth phase and then use the SA production preferential zone during no-growth conditions. The hypothesis was that this would help to stabilize the flux distributions described on the modeled surfaces and therefore maintain yields with higher process productivity as more cells would be present. Upon ending the feed, a stationary or non-growth phase would in theory be expected to show similar physiological and flux distribution behavior as described by the stationary modeled response surfaces and in this way enhance SA production with controlling AC production at high yields and process substrate conversion. This was achieved by designing a pseudo-exponential feeding profile with concentrated solutions of GLC and YE that considered substrate addition from the beginning of the fermentation to maintain the initial concentration conditions as long as possible. It is important to notice that all models consider YE as a unique metabolite so balancing flux for this complex substrate was just an approximation.

Figure 10d shows the fermentation profiles for the fed-batch optimized SA production process. Feed was performed from hour 3 (since calculated feed was previously too small for peristaltic operation) to hour 18 and controlled every 15 min manually, to match calculated growth and consumption parameters. GLC concentration was maintained near ≈75–80 g/L during the first 8–10 h of fermentation where an increase of GLC was observed up to 100 g/L concentration at hour 12. During this process, we also found a lower growth rate that could be responsible for the GLC accumulation, which was attributed to the manual control of the feed rate (that would lead to a significant overestimation of the feed over the time) and to the simultaneous feeding of similar amounts of YE and GLC (since individual component consumption cannot be calculated as it is taken as a simplified metabolite on models). Therefore, imbalances on feed may cause the exhaustion of crucial metabolic intermediates. Then the overestimated feed fluxes during the observed stall may cause an extracellular re-accumulation of these limiting components resulting in the second growth phase seen after 16 h and up to the 20th h of culture with a lower growth rate. Even though, it is still not clear the reason for this particular stall and further improvements on fed-batch operations could further give us insight on the strain behavior and enhance SA production. The first growth rate registered 0.90 h ^−1^, which is in range of the ones predicted by models (0.8–0.85 h ^−1^), while the second growth rate is only about 0.18 h ^−1^, suggesting limitation by an unknown substrate. Regarding the SA production rate, it also responded to this 8–16 h stall. Despite this, high GLC consumption rates on stationary phase were maintained and GLC was completely exhausted after only 24 h. A total of 180.5 g of GLC were consumed and 59.1 g/L of SA were produced. Although this titer is the highest obtained with this strain, it is 30% below the maximum titer reported on *E. coli* by Chandran et al. [5]. Moreover, the AC concentration was never found to be above 5 g/L, proving that process design was successful to limit the AC production even on this atypically high substrate conditions and compared to the batch culture where >15 g/L AC were accumulated. Furthermore, the process presented a global volumetric production rate of 2.45 g SA/L*h representing a 70% increase from the 1.43 gSA/L*h reported by Rodriguez et al. (2013)[2] and which is 20% higher than the previously reported industrial *E. coli* strains (2.04 gSA/L*h Chandran et al.[5]). Crucially, yields calculated by linear regression approximation were 0.40 for *Y*_*p*/*s*_ and 0.744 for *Y*_*p*/*x*_, which means that both yields were maintained along fermentation relative to batch conditions and suggest that carbon distribution along metabolic nodes relevant for SA production were maintained within reason. These yields are also in accordance with previous works with strains lacking the PTS and are still among the highest reported on *E. coli* [2, 14, 33, 36, 38, 41, 42].

## Discussion

Response surfaces showed the capacity to characterize correctly the physiological behavior of the AR36 strain. The observed increase on *X*_*max*_ mainly by [YE] and low increment by [GLC] is related to the fact that it is the only source of aromatic amino acids (Fig. 2a). This indicates that in all the experimental design area, YE can be taken as the limiting substrate for biomass production. This can also be observed on the fermentation profiles shown in Fig. 1, where the stationary phase of fermentations initiates always before limiting GLC concentrations. SA production is expected to follow the GLC consumption as it is the main source of carbon redirection to PEP and E4P, and this trend could be observed on Fig. 2b and c. However the maximum for SA production is found before maximizing consumption. This small difference can be explained by the Final AC ([AC] _*f*_), which tends to increase at higher initial concentrations of GLC along with a maximization tendency at concomitant smaller initial concentrations of YE (Fig. 2d). High [AC] can hinder the H ^+^ balance across the membrane and considering that AR36 uses the *galP* coded galactose-proton symporter for GLC transport, consumption could be compromised [2]. Also, higher [AC] makes ATP production costlier, and in consequence, also the ATP-dependent phosphorylation of GLC by glucokinase towards the glycolytic metabolism [2]. Also, AC production is commonly related to metabolic overflow and considering that AR36 strain lacks the *pykF* gene, higher [GLC] and increasing intracellular [PEP] and [PYR] could be causing the observed higher AC production and lower GLC consumption [36, 38, 40, 42–44]. Therefore, the lower values for GLC consumption and SA production can be explained by the high AC concentrations produced by the strain within this experimental design region (high initial GLC, low initial YE). It is therefore possible to allocate a virtual SA vs AC critical line near 100 g/L GLC concentration, where below this line SA production may be favored and above this line AC production becomes relevant. It is important to notice that the very high GLC concentrations used in all experiments would typically result in higher AC production and slow growth in *E. coli* [45, 46]. Nevertheless, it is known that the AR36 strain can grow and maintain SA production at high GLC concentrations, with relatively low AC production, as a result of the high constitutive expression of SA biosynthetic genes and the lack of carbon repression present in strains lacking PTS [44].

Regarding specific rates a diminution of the GLC consumption rate under increasing [YE] was also found. This could be related to YE components competing for transport energy or amino acid allocation for their transporters. This may be possible since as mentioned, AR36 does not present catabolic repression (as a consequence of *crrHI* operon deletion) and is capable to transport simultaneously various carbon sources in the presence of GLC after synthesis induction of alternative transporters by cell carbon scavenging signals [36, 40, 42, 44]. More over, at higher [YE], PYR consumption reactions could be kinetically saturated since greater alanine (ALA) YE derived concentrations could enter the CCM to PYR saturating this metabolite pool and reducing GLC consumption. In contrast, at the stationary phase $q_{glc}^{sta}$ presented higher values on higher initial concentration of both substrate sources. This behavior is expected for GLC, as higher concentrations of this substrate remain on the stationary phase and could be triggering higher consumption rates. However, it remains unclear why higher concentrations of initial YE could cause greater GLC stationary consumption rates. One possibility is that, higher initial [YE] could result in more oxaloacetate present on this phase increasing TCA activity and GLC consumption. This observation correlates with the behavior found for *Δ*[GLC] where only at high [YE], high [GLC] initial concentrations can be exhausted.

Regarding SA and AC production profiles it seems clear that as they are the main metabolic outputs for this strain they present the inverse maximization zones on experimental design area on both fermentation stages (Fig. 2f, h, i and j). This can be explained by the potential competition for carbon flux. These characteristics along with the biomass and the GLC related surfaces allowed to describe different output behavioral zones as described on the results section. However it is interesting that on the surfaces, it is possible to find similar consumption rates on opposing sides of the experimental design area with greatly different physiological outputs. Furthermore, the response surfaces morphologies have far from linear contours along the different physiological characteristics found on them. This could suggest the existence of metabolic state multiplicity similar to pseudo-stationary ones described by Namjoshi et al. on continuous bioreactors [47].The difference on response surface behaviors should derive from the dynamic properties, which produce different outcomes depending on the extracellular and intracellular metabolite concentrations and to the non-linearity associated with metabolic regulation [36, 42, 43, 47].

For the reasons described above and as the underlying characteristics of the systems were difficult to address only with external behavioral response surfaces, dynamic flux models were constructed. Cybernetic modeling was used mainly because interaction between cellular auto-regulated and inter-regulated subsystems (DNA, RNA, ENZYME) cannot be mechanistically described but some systematical characteristics can be approximately modeled [31, 48, 49]. Also, it is important to note that our media contains a non fully described compound substrate as YE, which was simplified as describe to only few metabolites resulting from its consumption by AR36. This simplification could not only impacts the growth phase, but also means that consumption of other YE components are not fully considered and may modify yields and rates. However, it is also noticeable that this simplification resulted enough to reasonably describe some behavioral characteristics on the fluxes found for AR36, which in consequence described the physiological outputs with reasonable accuracy. The latter observed responses are the results of a matrix made out from the network of the central carbon metabolism to which mathematical reduction and yield analysis from the previously determined parameters resulted on 6 elementary modes (EMs) modes for the exponential phase and 3 EMs for the stationary phase, and their combination across time renders the output described. This means that model is in essence the same for all 9 experiments and the approximation was performed on to the parameters that regulate their combination across time. Results suggest that with this EMs an estimated description of metabolic behavior can be made for the various initial conditions explored. The usage of the experimental design and surface rendering for the relative fluxes helps depict the metabolic behavior of the strain even with the errors described previously on individual points. Flux surfaces where then correlated to the physiological characterization as well as for behaviors known for this strain on the literature as discussed below.

An unusual flux distribution redirecting most of the GLC derived carbon through PPP was found. This may be possible on strain AR36 as it has the *zwf* gene overexpressed by a strong promoter on a high copy number plasmid [2]. Furthermore, FBA models by Chen et al. (2011)[32] have established that G6Pdh is rate limiting for PPP flux. Therefore, the high expression of *zwf* on AR36 strain could in fact be causing this low Pgi flux distribution. Despite this, a high total glycolytic flux is still found as described by Rodriguez et al. 2017 [44] as it was found that the operon-containing plasmid augmented the GLC consumption rate. The latter is in agreement to the high relative flux values of 75 to 88% towards PEP and PYR. On the modeled results this was possible since the high PPP flux was mainly redirected trough EDP to G3P and PYR. In this regard, it has been reported that on *pykF* mutants (such as AR36), fluxes through PPP are increased up to 79% by *pgi*, *pfkA* and *tpiA* down-regulation and *zwf*, *gnd* and *edd* concomitant up-regulations [50, 51]. For AR36, a low intracellular level of fructose-1,6-bisphosphate (FDP) was previously found by comparative metabolomics and explained as a consequence of TktA activity [42, 44]. But with the results here obtained it can be suggested that it could also be influenced by a high flux deviation into the PPP by G6Pdh. Although *pgi* mutants have been reported to have lower growth rates caused by NADPH accumulation redox imbalance, it also has been found that overexpression of NADPH-consuming pathways can recover the growth rate [51]. Therefore, in AR36 the high production of SA, requiring NADPH by *aroE* coded shikimate dehydrogenase, could be alleviating the NADPH imbalance and promoting higher growth rates in the presence of high PPP flux distributions. In this sense, the production of SA in this strain could act as an important driver of its own synthesis when the *zwf* gene is overexpressed alongside the SA biosynthetic genes during growth phase. In contrast, on the stationary phase although a high PPP flux distribution was also found, low EDP flux was also found while high glycolytic PEP producing reactions were maintained. However this could also be possible since higher fluxes through Tal were found and that could cause higher FDP concentrations compared to the growth phase, signaling the up-regulation of the downstream glycolytic genes [43].

These results imply that E4P is the limiting substrate for SA production even with *zwf* overexpression and a high flux redirection towards the PPP, as previously suggested [42–44]. The modeled flux ratio analysis suggest that sole overexpression of *zwf* is not sufficient for alleviating E4P limitation. Therefore, *edd* and/or *eda* genes could be attractive deletion targets to avoid undesired partitioning of PPP fluxes, along with exerting better control under *zwf* and *gnd* overexpression to obtain higher but controlled flux distributions towards E4P and SA. Following on, PpsA presented a near-zero flux within all experiments. Considering the PEP overabundance to E4P, it can be deduced that even when *ppsA* has been previously used as a target to enhance SA production [14, 36, 38, 52, 53], the overexpression of this gene in AR36 may not further increase SA production. Furthermore, it is possible that overexpression of *ppsA* on this genetic background could hinder growth and GLC consumption by reducing carbon flux towards TCA and other PEP derived pathways.

Also, an unusually high PoxB flux was found in this strain as consequence of the increased influx towards PYR. This is supported by the previous findings in other related PTS-deficient strains lacking carbon catabolism repression, where PoxB has been proposed to be the main AC producing enzyme [33, 36, 38]. Furthermore, an *arcA/arcB* mutation has been found for this strain lineage that could be making PoxB available for expression on earlier fermentation phases [33, 34, 36, 38, 40]. Other studies have also proposed PoxB as the main AC synthesizing enzyme under higher growth rates on accelerostats on other *E. coli* strains [54].

Regarding the AckA vs Acs AC consumption flux distribution, a high up-regulation of *acs* and *poxB* genes has been observed to occur as a response to PTS inactivation on this strain, suggesting that carbon cycling on AC occurs through Acs [36, 38, 40]. Therefore, it is probable that the model depicted the incorrect or inverse distribution around these reactions during growth. Moreover, the AR36 lineage strains does not show the expected PTS mutant low cAMP concentrations, probably due to AC cycling through Acs restoring cAMP along with adelynate cylase, which in turn has been also found to be up-regulated on these PTS ^−^ strains during growth [36–38, 40]. It is interesting to find that the EMs used for stationary phase rendered Acs as the principal reaction responsible for redirecting AC to ACCOA (Fig. 9c and d), probably due to less ATP demand on this phase. Furthermore, the combination of the export and import AC surfaces strongly correlates with the $q_{b}^{exp}$ approximated AC production rate on the physiological analysis presented before (Fig. 2i), where high extracellular AC production is found towards high initial [GLC] and towards high initial [YE]. Since both fluxes are present, it can be proposed that an AC production/consumption futile cycle could potentially be relieving part of the metabolic overflow on the CCM [33, 36, 37], as has previously been reported to help adjusting imbalances between glycolysis and the TCA activities [43].

TCA activity on its part, showed the unexpected IcdA reverse reaction. This could be attributed to the YE derived GLU entering the TCA through *α*-ketoglutarate (AKG) by Gdh, but since this compound is used also for biomass precursor formation, only if it is consumed on excess it will enter the CCM. This effect could also be increased by other YE derived amino acids entering CCM. On that regard, reports on complex media have shown a tendency to favor extracellular amino acid consumption and catabolism through AKG with the concomitant up-regulation of biomass producing pathways [55]. Also, the high GSP and malic enzyme activities suggest that this strain counteracts metabolic saturation by trying to relieve the PYR saturation by assimilating more ACCOA through lower CCM pathways. This may be the result of a selective pressure to the high osmotic pressure on this media to recover high substrate consumption rates and consume the highly concentrated substrates faster. In fact, high osmotic stress conditions have been found to increment the GSP activity and to reduce the *icdA*/*aceA* coded enzymes ratio, favoring the production of biomass building blocks [56]. Therefore, the high anaplerotic reactions (APR) fluxes along with GSP found also may contribute to maintain high biomass and SA production since this could help to minimize toxic AC production by ACCOA fast consumption,along with a response to osmotic pressure [43, 56]. This behavior may be expected as with the higher [GLC] more stress upregulating GSP could be used to relieve carbon flux from the PYR and ACCOA nodes [33, 36–38, 40, 43]. Furthermore, the excess carbon arriving to MAL then seems to be redirected to PYR and PEP by the previously detailed MaeB, MaeA and PckA reactions. MaeB presents greater fluxes possibly due to consuming the excess NADPH produced by the high PPP flux conditions [51].

Overall, the dynamic cybernetic model approach seems to unveil behaviors that are in accordance to the physiological observations and to the knowledge available for this laboratory evolved strain lacking the mayor GLC transport and therefore catabolite repression. The behavior of the calculated fluxes surfaces during the growth phase is in agreement with the results obtained with the physiological surface analysis. Particularly, on many of the IE and ME surfaces, critical surface saddle points between 110–115 g/L of initial [GLC] and between 35–40 g/L of initial [YE] have been found. It is interesting to notice that 9 EMs were enough to describe all the patterns conformed by the surfaces by only modifying the parameters which alter their combination across time. These changes, although cannot be used to describe regulatory mechanisms, unveil relevant systems characteristics and interestingly also suggest the existence of metabolic state multiplicity derived from changing extracellular conditions [47].

## Conclusion

In this report we describe a modeling approach for a PTS ^−^ laboratory evolved *E. coli* engineered strain for SA overproduction [2, 33, 36, 38, 40] to study and characterize its physiological and metabolic responses to variant complex substrate concentration. The constructed models were able to describe in good agreement the individual experimental fermentations performed with this strain. Three-dimensional response surfaces were constructed with polynomial equations allowing to morphologically describe the cell output behavior under the experimental conditions. It was found that the production strain responds differently to initial substrate concentrations, allocating resources in different ways. This was inferred since regulation along variations from complex media substrate conditions did not affect linearly the performance of the strain, but showed refined nonlinear borders between predominant outcomes. For these reasons a dynamic cybernetic model was constructed and their flux distributions studied and compared to the physiological models. The constructed dynamic metabolic model was able to follow the extracellular experimental behaviors and three-dimensional response surfaces for relative flux distributions were used to unveil insights into the strain metabolism.

Flux distributions helped to explain the previously observed low intracellular level of fructose-1, 6-bisphosphate (FDP) reports by unveiling a high PPP flux during all fermentation processes. MaeB high relative flux, potentially helps alleviate the NADPH redox imbalance caused by NADPH-dependent SA production and contributes to growth rate recovery [2]. Flux distributions allocated AC production, GSP and APR with high fluxes to contend with the metabolic stress produced by the concentrated substrates on media helping to relieve PYR and ACCOA overflow. PoxB was found to be the predominant AC production enzymatic reaction on this strain under high substrate conditions. An AR36 *ΔpoxB* strain was constructed and cultured showing the loss of growth phase AC peak and along with lower growth, GLC consumption rates probably by greater PYR accumulation on this derivative [33, 36–38, 44]. Model analysis also found that *edd* and/or *eda* could be targeted for deletion, along with a better control under *zwf* overexpression and *gnd* expression, to obtain better flux distributions towards E4P and SA production. Also, *ppsA* overexpression and other modifications involving higher PEP accumulation may not improve SA production until E4P limitation is resolved on this strain. Dynamic models were found to be in accordance to the physiological observations and the knowledge available for AR36 a PTS ^−^ strain lacking catabolite repression, and were useful to allocate preferential metabolism output zones within the experimental design area that correlated in good agreement with the zones observed during the physiological model characterization.

Finally, to assess the utility for SA production enhancement with all the previously described models, a fed-batch fermentation regime was designed. An unusual operation was employed to maintain initial media conditions which would in theory help maintain metabolic an physiological conditions. The fed-batch fermentation resulted in a 40% titer and 70% volumetric productivity increases while preserving product and biomass yields. Process presented yield values among the highest yields reported and presented the highest productivity reported on *E. coli* AR36. Although reports concerning other strains have shown higher titers [57], this report was centered on the mathematical approach to further extend *E. coli* production capabilities. On that matter, the model implemented in this report is the first approximation to render flux distributions for this *E. coli* PTS ^−^ strain under high-substrate production conditions and one of the first approaches towards modeling *E. coli* metabolism in complex media containing high concentrations of GLC and YE.

## Methods

### Strain, cultivation and analytical procedures

*E. coli* AR36 strain constructed by Rodríguez et al. (2013) [2] was used for all experiments and calculations. AR36 is an *E. coli* PB12 laboratory evolved derivative lacking the phosphoenolpryruvate:carbohydrate phosphotransferase system (PTS) [33, 36, 38, 40]. AR36 carries additional inactivations in *aroK*, *aroL*, *pykF* and *lacI* genes, and contains a high copy number plasmid with the strong *trc* promoter controlling transcription of a six-gene operon composed of genes: *aroB*, *tktA*, *aroG*, *aroE*, *aroD* and *zwf* [2]. This strain is an aromatic amino acid auxotroph and therefore it must be cultured on supplemented media. Yeast extract (YE) (BD Bacto) and GLC (Fermentas) were used as nitrogen and carbon sources [2]. All cultures were performed on 0.5 *L* working volume bioreactors with AD 1010 controllers (Applikon). Bioreactors were operated as batch processes at 37 ^∘^*C* and 1 *vvm* aeration. Dissolved oxygen tension (DOT) was maintained above 20% by an agitation cascade control between 500 and 1200 *rpm*. pH was maintained at 7 by means of *NH*_4_*OH* and *H*_3_*PO*_4_ addition. Other media compounds, salts, buffer and antibiotics were used as previously described [2].

Physiological behavior characterization was performed with a central composite design experimental matrix with 3 levels for each substrate source. Experimental condition levels were: 75, 100 and 125 *g*/*L* for GLC and 15, 30 and 45 *g*/*L* for YE. Nine experiments were conducted with the central point 100 *g*/*L* GLC and 30 *g*/*L* YE, tested by triplicate to approximate the experimental design standard deviation. Fermentations were sampled every 2 h during the first 12 h, and every 4 to 6 h after this point. Each sample was used to determine biomass, GLC, SA and AC. Biomass was determined by optical density measurements at 600 *nm* with a DU700 Beckman spectrophotometer. GLC, SA and AC were determined by HPLC with a Waters equipment(600E quaternary pump, 717 automatic injector, 2410 refraction index an 966 photodiode array detectors) and an aminex HPX-87H column (300 x 7.8 *mm*; 9 *μm*), using 5 *mM* *H*_2_*SO*_4_ as mobile phase at 50 ^∘^*C*; either UV or refractive index detectors were used for qualitative and quantitative determination. All measured parameters were volumetrically corrected for the acid or base added by pH control pumps.

### Calculation of fermentation parameters

For fermentation data parametrization and analysis, a set of modeling approaches was constructed. The maximum growth rate *μ*_*max*_ and maximum biomass *X*_*max*_ were obtained by adjusting a logistic growth model to experimental data. Since the fermentation processes use complex media, calculation of yields and production/consumption rates by classical calculations were difficult to address. Therefore, to provide a more accurate parametrization, GLC consumption and SA production integrated models were constructed. The integrated model equations used were:

1 $$\begin{array}{*{20}l} X_{(t)}&=\frac{X_{0}e^{\mu_{max}t}}{1-\left(\frac{X_{0}}{X_{max}}\left(1-e^{\mu_{max}t}\right)\right)}  \end{array} $$

2 $$\begin{array}{*{20}l} S_{(t)} &= S_{(t-1)} -\left[\left(q_{glc}^{exp}X_{(t)}(\Delta t)\right)\left(1-\frac{X_{(t)}}{X_{max}}\right)\right]\\ &\quad-\left[\left(q_{glc}^{sta}X_{(t)}(\Delta t)\right)\left(\frac{X_{(t)}}{X_{max}}\right)\right]  \end{array} $$

3 $$\begin{array}{*{20}l} P_{(t)} &= P_{(t-1)} +\left[\left(q_{sa}^{exp}X_{(t)}(\Delta t)\right) \left(1-\frac{X_{(t)}}{X_{max}}\right)\right]\\ & \quad+\left[\left(q_{sa}^{sta}X_{(t)}(\Delta t)\right)\left(\frac{X_{(t)}}{X_{max}}\right)\right] \end{array} $$

where *S* refers to substrate, in this case GLC, and *P* refers to product, SA on this experimental design. *X*_(*t*)_ is the biomass calculated at time *t* by the logistic growth model and *X*_*max*_ is the maximum biomass parameter. $q_{glc}^{exp}$, and $q_{sa}^{exp}$ are the specific exponential rates for GLC consumption and SA production, respectively. $q_{glc}^{sta}$, and $q_{sa}^{sta}$ are the specific stationary rates for GLC consumption and SA production, respectively. The participation of each exponential or stationary rates across time is regulated by the terminus describing the biomass and maximum biomass ratio correlated to the biomass logistic model. The production and consumption rate parameters were approximated by the sum of the square error (SSE) minimization against experimental data using MATLAB programming. Product/substrate and product/biomass yields were estimated from the obtained specific rates.

Models constructed were tested for their experimental data approximation by an error estimation calculated by the relation between the sum of the square error (SSE) and the sum of the square of the experimental points (SSEP). Model approximation was then mathematically described by the linear regression between experimental points and model points. A percentile deviation from the expected slope (1 for experimental and model equality) descriptive indicator constructed from regression (SDP) as well as Pearson regression coefficient (describing dispersion) and the regression significance proved by *p*-value statistics were used to qualify the acceptance of models as descriptors for the experimentally observed behavior.

AC presented a dynamic behavior (simultaneous production and consumption) in all fermentations that could not be described by any of the previously described equations. Nevertheless, initial exponential $\left (q_{b}^{exp}\right)$ and initial stationary $\left (q_{b}^{sta}\right)$ approximated production rates were calculated with the following equations:

4 $$\begin{array}{*{20}l} q_{b}^{exp}&=\mu_{max} \times Y^{exp}_{ac/x} \end{array} $$

5 $$\begin{array}{*{20}l}  q_{b}^{sta}&=Q^{sta}_{b}/X_{max} \end{array} $$

where yield was calculated by linear regression for AC vs biomass and volumetric rate was calculated by linear regression for AC vs time on the first experimental data points for each phase.

Parameters describing the physiological behavior were then used to construct individual three-dimensional surfaces. A second-order bivariate polynomial equation was used for surface construction and its approximation was addressed and qualified by regression coefficients, *p*-values and square sums of error and percentile error. Surfaces were validated by prediction of parameters for three fermentations not contained on the set of the experimental design (75:20, 80:40 and 115:45 GLC:YE initial conditions). Surface calculated parameters were introduced to the logistic biomass, consumption and production models and compared to the experimental data sets. Surface calculated parameters were also compared to the ones calculated directly from experimental data by error percentage, estimated by the ratio between standard deviation between each calculation and experimental parameter. A two-tailed *t*-student test using the experimental design standard deviation, calculated from central point, was used to determine if the experimental parameters and surface predicted parameters were significantly different.

### Dynamic metabolic flux model construction

Metabolic flux distribution was constructed with the use of a dynamic cybernetic model approach developed by Ramkrishna et al. [31, 48, 49], which has proved to be useful to address dynamic changes on fluxes when information on mechanistic details of regulatory processes is scarce or suboptimal [58]. The cybernetic modeling introduces regulation by the use of two vectors *u*≡[*u*_1_,*u*_2_,*u*_3_,...,*u*_*m*_] and *v*≡[*v*_1_,*v*_2_,*v*_3_,...,*v*_*m*_] referring to them as cybernetic variables, associated with fractional allocations of resources for enzyme synthesis and activity, respectively [31, 48, 49]. These variables are calculated along the fermentation and modify the participation of each elementary mode obtained from the stoichiometric matrix analysis. These elementary modes (EMs) are sets of non-decomposable pathways consisting of minimal sets of reactions that describe all the cellular metabolic routes. A subset of EMs must then be extracted to describe metabolic behavior on a parametrically achievable scale. Therefore, elementary mode analysis (EMA) must be performed to find the minimal set of EMs that can describe the behavior expected from the specific constraints imposed by either the strain or experimental conditions. Cybernetic models then calculate flux rates for each EMs described as sets of Michaelis-Menten type equations where a relative enzyme concentration and biomass conform the maximum rate, modified at each time by the cybernetic variable *v*. The relative enzyme concentration is calculated by another Michaelis-Menten type equation that considers a maximum enzymatic production rate and a disappearance rate, regulated by the cybernetic variable *u*. The cybernetic variables are regulated by an objective function, evaluating the outputs at any given time *t* between all EMs, increasing priority on the next time step *t*+*Δt* to the better performing EM by a matching law strategy. In this way, the cybernetic models can take into account dynamic regulation with respect to a specific cell metabolic objective, such as growth rate maximization or carbon uptake maximization, even with little information on the mechanistic particularities to its function, allowing for dynamic flux distribution modeling [31, 48, 49].

In this work, a CCM network was constructed from 60 reactions, 44 internal metabolites and 6 external metabolites, accounting for the Embden-Meyerhoff-Parnas pathway (EMP), the Pentose Phosphate pathway (PPP), Tricarboxylic Acid Cycle (TCA), Pyruvate Metabolism, Anaplerotic Reactions, respiration and energetic reactions, YE components uptake reactions, SA biosynthesis reactions and biomass generation reactions. External metabolites defined were AC, GLC, SA, YE, biomass, and maintenance. YE consumption was introduced to the network reaction as a metabolite and its consumption derived into biomass precursor (BIOMp), aromatic amino acids (taken as a unique metabolite), alanine (ALA), and glutamic acid (GLU). The stoichiometric values for YE conversion to these metabolites were estimated from the average composition described by the manufacturer, where BIOMp was taken as the rest of amino acids that account to produce proteins contained on biomass. EMs computation was made with efmtool protocol [59] embedded on MATLAB [60]. For EMA two EMs families were constructed, the first family contained the exponentially preferred EMs by only selecting the ones that contained simultaneous GLC and YE consumption and constrained to produce SA. The second family of EMs was selected from the ones containing GLC consumption and simultaneous production of SA and constrained to not consume YE, which will be preferred on the stationary phase of fermentations. Yield analysis reduction by convex hull volume was performed as described as by Song et. al. [60] to find the minimal subset of EMs. Experimental design central point yields were used for this analysis. Yield analysis around the Phosphoglucose isomerase (Pgi)/ Glucose 6-phosphate-1-dehydrogenase (G6Pdh) node was studied with values stated as: non-constrained, 0.25, 0.5, 0.75 and 0.90 Pgi/GalP yield or flux normalized to GLC uptake. This generated 5 flux distributions sets. All model sets were evaluated by Pearson linear regression coefficients between experimental points and model points, slope deviation and their significance was proved by p-value statistics and error. The set with better behavior to experimental data was used for further analysis. Reduced EMs reaction rates were described by sets of Michaelis-Menten equations modified to couple families to each fermentation phase (exponential or stationary) by a terminus similar to the physiological consumption/production models described before, and with an added terminus to represent AC inhibition. Model rate equations were constructed as follow:

6 $$\begin{array}{*{20}l}  r_{i}^{M}&=\left(\frac{k_{max,i}^{M} [GLC]_{(t)}}{K_{m,i}^{M}+[GLC]_{(t)}}\right) \left(1+\frac{[AC]_{(t)}}{K_{I}^{ac}}\right)^{-1} \left(1-\frac{X_{(t)}}{X_{max}} \right) \end{array} $$

7 $$\begin{array}{*{20}l}  r_{i}^{G}&=\left(\frac{k_{max,i}^{G} [GLC]_{(t)}}{K_{m,i}^{G}+[GLC]_{(t)}}\right) \left(1+\frac{[AC]_{(t)}}{K_{I}^{ac}}\right)^{-1} \left(\frac{X_{(t)}}{X_{max}} \right) \end{array} $$

where indexes *M* and *G* refer to the exponential (mixed consumption) family and stationary (glucose only consumption) family, respectively. Index *i* refers to each *i*^*th*^ EM of each family, *K*_*max*_ and *K*_*m*_ are the Michaelis-Menten parameters, $K_{I}^{ac}$ is the inhibition coefficient and *X*_*max*_ refers to the maximum biomass calculated from the previously calculated logistic growth model for each experimental point. [*GLC*]_(*t*)_, [*AC*]_(*t*)_, *X*_(*t*)_ refer to the GLC, AC and biomass concentrations at each time *t* in *mmol*/*L* for the first two and *g*/*L* for the biomass. Initial relative enzyme concentration ratio was set to 0.95 for the first EM, 0.5 for the EMs remaining of the exponential family, and 0.1 for the EMs of the stationary family. These values were set in this way as the first EM was the one that comprised AC production and was inferred to be the initially preferred one, due to the observed rapid increase in extracellular AC acid at the initial phases for all fermentations. In addition, EMs of the second family were chosen to be smaller as they are expected to be more relevant at later stages of fermentation. All other cybernetic model parameters for enzyme production and decay rates were set as described by Ramkrishna et al. [31, 48, 58, 60]. Flux rate equation parameters *K*_*max*_ and *K*_*m*_ were approximated with a genetic algorithm (Additional file 2). Briefly, the Matlab algorithm started with assigning *K*_*max*_ and *Km* initial values of 1 and 10, respectively for every EM. Then, by perturbation of one parameter at a time by a random numeric factor, 18 parameter sets were obtained. Subsequently, the sets were used for 200 step SSE driven nonlinear numeric minimization algorithms to generate new daughter *K*_*max*_ and *K*_*m*_ parameter model sets. From these daughter models, the set with the lowest SSE was extracted and crossed with the second lowest SSE set by acquiring the value of its perturbed parameter (*K*_*max*_ or *K*_*m*_). This inter-crossed set passed onto the next generation where another round of individual parameter perturbations was made. The algorithm was cycled until SSE was found constant (less than 20 cycles in all cases). Finally, these parameters were subjected to a final SSE nonlinear numeric minimization to model the flux rates of each EM and the final metabolic dynamic flux model for each fermentation.

Flux distributions were used to construct three-dimensional behavioral surfaces with the second order two variable polynomial equation at three fermentation times: initial exponential (IEx), mid exponential (MEx) and mid stationary (MSt). Calculation of MEx time was made by obtaining the maximum point of the second derivative vs. time for each biomass model, IEx was set as the mid time between *t*=0 and MEx time, and MSt as the middle point between the end of the fermentation and the initial time of stationary phase. Constructed surfaces were statistically qualified and used to analyze and study the behavior of strain metabolism and SA production.

### Bioprocess design for SA productivity enhancement on strain AR36

To assess the utility of the metabolic models developed, a fermentation process was designed with the information acquired by physiological models and flux distribution surfaces to optimize SA productivity. Process was designed to maintain constant the initial GLC:YE conditions. This would in theory, maintain constant the cybernetic variables (which are a representation of the regulation parameters) and therefore the internal flux distributions accordingly. Initial conditions around 80:40 g/L GLC:YE were used. A pseudo-exponential flux was operated and regulated by a peristaltic pump manually set every 15 min to calculated exponential feeding needs, with measured GLC and calculated growth and consumption rates. Feed consisted on two simultaneously added solutions, one containing mineral media with GLC 400 g/L and the second containing phosphate buffer solution with YE at 400 g/L. After 12 h, the feed was stopped and the fermentation was allowed to enter stationary phase to consume the remaining GLC. Oxygen was added when needed to maintain dissolved oxygen tension (DOT) over 20% along with an agitation cascade. Other media compounds, salts, buffer and antibiotics were used as described by Rodriguez et. al. [2] and added through the feeding solutions to avoid dilution.

## Additional files

Additional file 1 Model and validation data, parameters and statistical values. Description: Parameters and statistical value tables for physiological models. Response surface parameters and statistical values for the polynomial approximation. Response surface prediction validation data. Response surface critical points calculations. Dynamic Flux models parameters and statistics. (PDF 186 kb)

Additional file 2 Metabolic network definition for dynamic model. Description: Definition of reactions, internal and external metabolites comprehending the metabolic network. Matlab program section for the genetic algorithm used for parameter approximation. (PDF 173 kb)

Additional file 3 Response surfaces and contour plots for all fluxes. Description: Response surfaces and contour plots for all reactions detailed on the metabolic network for initial exponential, mid exponential and mid stationary fermentation stages. (PDF 25,629 kb)

Additional file 4 Strains, plasmids and oligonucleotides used on this work, AR36 *poxB*^−^ figures. Description: Table for all plasmids and oligonucleotides used. AR36 *Δ*poxB strain construction. Fermentation profiles for AR36 *Δ*poxB and AR36 on highly concentrated substrate media. (PDF 215 kb)
