# Supplementary material for: Metabolic modeling and response surface analysis of an Escherichia coli strain engineered for shikimic acid production
Source: BMC Syst Biol. 2018 Nov 12;12:102. doi: 10.1186/s12918-018-0632-4 (PMC6233605; doi:10.1186/s12918-018-0632-4)
Supplement: Supplementary file 1 — Model and validation data, parameters and statistical values. Description: Parameters and statistical value tables for physiological models. Response surface parameters and statistical values for the polynomial approximation. Response surface prediction validation data. Response surface critical points calculations. Dynamic Flux models parameters and statistics. (PDF 186 kb) [file 12918_2018_632_MOESM1_ESM.pdf]

SUPPLEMENTARY MATERIAL 1

Metabolic modeling and response surface analysis for an engineered Escherichia coli for shikimic acid production

Juan A. Martínez, Alberto Rodriguez, Fabian Moreno, Noemí Flores, Alvaro R. Lara, Octavio T. Ramírez, Guillermo Gosset and Francisco Bolivar

Model, and validation data, parameters and statistical values

List of Tables

1 Experimental design parameters obtained by model calculations and their statistical values . . . 3
2 Response surface parameters obtained and their statistical values . . 4
3 Critical points calculated on surfaces under the limitations of the experimental design . . . 5
4 Validation design parameters obtained by model calculations and their statistical values to experimental data . . . 5
5 Experimental vs Response surface predictions and statistical values calculated for validation fermentations . . . 6
6 Dynamic Flux Model their statistical parameters to experimental data 6
7 Dynamic Flux Model Response Surfaces parameter values for Initial Exponential point . . . 7
8 Dynamic Flux Model Response Surfaces parameter values for Mid Exponential point . . . 8
9 Dynamic Flux Model Response Surfaces parameter values for Mid Stationary point . . . 9
10 Dynamic Flux Model Response Surfaces critical points for Initial Exponential time point . . . 10

11 Dynamic Flux Model Response Surfaces critical points for Mid Exponential time point 11
12 Dynamic Flux Model Response Surfaces critical points for Mid Stationary time point 12

Table 1 Experimental design parameters obtained by model calculations and their statistical values

GLC	75	75	75	100	100	100	125	125	125
YE	15	30	45	15	30	45	15	30	45
Max Biomass	6.45	12.11	17.48	6.30	12.80	18.16	6.90	13.84	17.82
Consumed GLC	69.6	70.0	71.1	71.0	99.9	97.3	67.3	89.8	121.7
Final SA	20.9	24.1	22.0	21.3	32.8	33.2	20.5	28.9	31.2
Final AC	8.2	0.3	0.1	15.3	9.2	2.3	10.9	13.0	10.7
μ_{max}	0.679	0.791	0.894	0.616	0.579	0.604	0.637	0.600	0.738
q_s^{exp}	1.211	0.882	1.000	1.960	1.240	0.912	0.600	0.445	0.762
q_p^{exp}	0.444	0.331	0.225	0.517	0.453	0.216	0.520	0.348	0.234
q_s^{sta}	0.324	0.394	0.399	0.275	0.351	0.447	0.467	0.301	0.445
q_p^{sta}	0.125	0.161	0.213	0.125	0.135	0.167	0.122	0.085	0.147
$Y_{p/s}$	0.366	0.376	0.225	0.264	0.362	0.237	0.866	0.783	0.307
$Y_{p/x}$	0.654	0.419	0.252	0.839	0.762	0.358	0.815	0.580	0.317
$Y_{x/s}$	0.561	0.897	0.894	0.314	0.487	0.662	1.061	1.349	0.969
Biomass %ERR	0.10	0.10	0.14	0.07	1.88	0.31	0.34	0.28	1.27
GLC %ERR	0.02	0.23	0.02	0.07	0.72	0.03	0.14	0.66	0.12
SA %ERR	0.26	0.09	0.19	0.41	3.58	0.02	0.43	0.08	0.91
Biomass %SPD	0.02	0.74	1.85	0.53	2.48	0.81	1.05	0.62	1.64
GLC %SPD	0.00	0.00	0.00	0.00	1.63	0.00	4.70	0.00	1.05
SA %SPD	0.63	0.26	0.00	0.93	0.75	0.00	3.42	0.00	6.33
Biomass R^2	0.996	0.997	0.998	0.997	0.950	0.993	0.988	0.992	0.962
GLC R^2	0.998	0.993	0.999	0.993	0.977	0.999	0.983	0.962	0.997
SA R^2	0.995	0.998	0.996	0.991	0.932	1.000	0.992	0.998	0.986
X $p-value$	3E-11	5E-09	3E-09	9E-12	1E-20	5E-10	3E-09	8E-09	2E-09
GLC $p-value$	8E-14	6E-09	1E-11	2E-12	2E-26	6E-13	2E-11	6E-08	4E-15
SA $p-value$	5E-12	7E-11	6E-10	1E-11	3E-19	4E-15	3E-13	1E-13	1.0E-11

Table 2 Response surface parameters obtained and their statistical values

	a	b	c	d	e	f	R2	pval	SSE	%ERR
Max Biomass	-2.974	1.5E-02	5.8E-01	2.2E-05	-3.3E-03	-7.2E-05	0.9977	2E-09	0.879	0.06
Consumed GLC	-14.491	1.9E+00	-1.7E+00	-1.2E-02	-1.6E-02	3.5E-02	0.9733	1E-05	152.702	0.23
Final SA	-53.429	1.3E+00	6.4E-01	-7.2E-03	-1.7E-02	6.3E-03	0.9668	2E-05	15.107	0.24
Final AC	-12.406	5.8E-01	-8.7E-01	-2.8E-03	1.7E-03	5.3E-03	0.9195	5E-04	38.682	4.85
μ_{max}	2.632	-4.0E-02	8.3E-04	2.0E-04	1.7E-04	-7.6E-05	0.9272	3E-04	0.013	0.29
q_s^{exp}	-4.805	1.6E-01	-9.5E-02	-8.9E-04	9.7E-04	2.5E-04	0.8818	2E-03	0.349	3.30
q_p^{exp}	-0.319	1.6E-02	4.6E-04	-7.2E-05	-8.2E-05	-4.5E-05	0.9765	6E-06	0.006	0.43
q_s^{sta}	0.663	-7.2E-03	-2.9E-03	4.9E-05	2.0E-04	-6.4E-05	0.6899	4E-02	0.020	1.50
q_p^{sta}	0.143	1.9E-04	-2.1E-04	4.8E-07	1.0E-04	-4.2E-05	0.9614	4E-05	0.001	0.41
$Y_{p/s}$	1.797	-4.9E-02	5.4E-02	3.2E-04	-5.8E-04	-2.8E-04	0.9546	6E-05	0.040	1.95
$Y_{p/x}$	-1.847	5.1E-02	4.0E-03	-2.3E-04	-2.1E-04	-6.5E-05	0.9822	2E-06	0.014	0.45
$Y_{x/s}$	5.679	-1.3E-01	8.0E-02	7.5E-04	-7.4E-04	-2.8E-04	0.9665	2E-05	0.055	0.83

Table 3 Critical points calculated on surfaces under the limitations of the experimental design

	X	Y	Z	H	D	Type
Max Biomass	N.A.	N.A.	N.A.	N.A.	N.A.	N.A.
Consumed GLC	N.A.	N.A.	N.A.	N.A.	N.A.	N.A.
Final SA	110.63	39.85	33.41	4.4E-04	-1.4E-02	Maximum
Final AC	N.A.	N.A.	N.A.	N.A.	N.A.	N.A.
μ_{max}	104.82	21.09	0.553	1.3E-07	4.0E-04	Miminum
q_s^{exp}	96.12	36.77	1.200	-3.5E-06	-1.8E-03	Saddle point
q_p^{exp}	N.A.	N.A.	N.A.	N.A.	N.A.	N.A.
q_s^{sta}	88.01	21.66	0.314	3.4E-08	9.8E-05	Minimum
q_p^{sta}	N.A.	N.A.	N.A.	N.A.	N.A.	N.A.
$Y_{p/s}$	87.90	25.92	0.351	-8.1E-07	6.4E-04	Saddle point
$Y_{p/x}$	N.A.	N.A.	N.A.	N.A.	N.A.	N.A.
$Y_{x/s}$	96.38	35.08	0.604	-2.3E-06	1.5E-03	Saddle point

Table 4 Validation design parameters obtained by model calculations and their statistical values to experimental data

GLC	75	80	115
YE	20	40	45
Max Biomass	8.4	16.0	18.1
Consumed GLC	70.7	78.2	113.8
Final SA	22.1	26.6	33.0
Final AC	6.4	0.9	8.7
μ_{max}	0.726	0.771	0.651
q_s^{exp}	1.166	0.967	0.989
q_p^{exp}	0.416	0.276	0.239
q_s^{sta}	0.322	0.393	0.416
q_p^{sta}	0.134	0.182	0.152
$Y_{p/s}$	0.363	0.287	0.231
$Y_{p/x}$	0.589	0.377	0.378
$Y_{x/s}$	0.685	0.809	0.737
Biomass %ERR	0.91	0.39	0.14
GLC %ERR	0.04	0.08	0.05
SA %ERR	0.26	0.04	0.20
Biomass %SPD	0.35	0.39	0.34
GLC %SPD	0.00	0.00	0.00
SA %SPD	0.87	0.14	0.19
Biomass R^2	0.963	0.985	0.996
GLC R^2	0.998	0.997	0.998
SA R^2	0.994	0.999	0.996
X $p-value$	1E-08	1E-09	3E-12
GLC $p-value$	2E-15	5E-13	1E-13
SA $p-value$	1E-12	6E-15	4E-12

Table 5 Experimental vs Response surface predictions and statistical values calculated for validation fermentations

	75:20				80:40				115:45			
	Exp.	Model	%Err	$p-val$	Exp.	Model	%Err	$p-val$	Exp.	Model	%Err	$p-val$
Max Biomass	9.30	8.40	6.86	0.91	14.30	16.03	8.55	0.03	19.76	18.05	6.12	0.97
Consumed GLC	70.03	70.70	0.68	0.41	80.11	78.19	1.70	0.72	115.76	113.84	1.17	0.72
Final SA	24.62	22.13	7.15	0.68	33.19	26.62	13.99	0.85	31.84	32.96	2.49	0.42
Final AC	5.25	6.45	16.20	0.08	0.00	0.85	N.A.	0.13	4.90	8.73	55.21	0.01
μ_{max}	0.62	0.73	12.58	0.13	0.78	0.77	0.79	0.54	0.55	0.65	12.30	0.15
q_s^{exp}	0.89	1.17	22.30	0.21	0.93	0.97	3.16	0.45	0.73	0.99	25.64	0.22
q_p^{exp}	0.36	0.42	9.81	0.22	0.32	0.28	10.55	0.78	0.24	0.24	1.67	0.54
q_s^{sta}	0.33	0.32	2.33	0.54	0.38	0.39	2.74	0.45	0.41	0.42	1.15	0.48
q_p^{sta}	0.15	0.13	5.90	0.59	0.18	0.18	0.50	0.51	0.15	0.15	2.25	0.46
$Y_{p/s}$	0.41	0.36	8.46	0.95	0.35	0.29	12.83	0.97	0.34	0.23	22.19	0.99
$Y_{p/x}$	0.59	0.59	0.27	0.51	0.42	0.38	6.71	0.64	0.44	0.38	10.06	0.72
$Y_{x/s}$	0.70	0.68	1.13	0.57	0.84	0.81	2.75	0.70	0.76	0.74	2.55	0.68

Table 6 Dynamic Flux Model their statistical parameters to experimental data

GLC	75	75	75	100	100	100	125	125	125
YE	15	30	45	15	30	45	15	30	45
Biomass %ERR	0.15	0.44	0.65	0.34	2.28	1.02	0.54	0.30	1.78
GLC %ERR	0.56	2.13	1.51	1.42	0.93	1.47	0.30	1.14	1.03
SA %ERR	1.21	1.37	3.88	1.57	3.48	2.46	1.76	0.25	4.19
AC %ERR	0.71	0.35	1.11	1.29	10.53	8.75	3.57	4.18	5.33
Biomass %slope	0.16	5.88	3.89	0.69	1.57	8.10	3.21	2.68	11.00
GLC %slope	1.07	15.95	19.56	21.39	10.94	16.11	3.42	3.00	6.33
SA %slope	3.47	3.79	4.69	9.93	12.28	2.30	5.76	1.13	13.13
AC %slope	2.42	2.19	3.62	4.95	21.55	42.75	9.00	5.22	1.31
Biomass R^2	0.997	0.995	0.995	0.995	0.972	0.992	0.992	0.996	0.977
GLC R^2	0.993	0.986	0.980	0.968	0.988	0.980	0.987	0.974	0.989
SA R^2	0.990	0.988	0.968	0.990	0.969	0.982	0.985	0.997	0.980
ACR^2	0.996	0.997	0.988	0.992	0.900	0.923	0.973	0.964	0.933
X $p-val$	1E-11	3E-08	3E-08	2E-11	2E-20	2E-08	2E-11	1E-10	9E-09
GLC $p-val$	1E-09	1E-06	4E-06	2E-07	5E-26	6E-07	4E-10	4E-07	2E-10
SA $p-val$	6E-09	6E-07	2E-05	1E-09	8E-20	4E-07	1E-09	2E-11	4E-09
AC $p-val$	1E-10	4E-09	6E-07	3E-10	3E-12	1E-04	2E-08	2E-06	3E-06

Table 7 Dynamic Flux Model Response Surfaces parameter values for Initial Exponential point

	a	b	c	d	e	f	R2	pval	SSE
Maint	-3.70E-01	5.07E-02	-1.08E-01	-2.72E-04	1.31E-03	7.73E-05	0.9383	2E-04	0.116
Biomass P	3.65E-01	-4.23E-03	1.61E-03	1.72E-05	-4.09E-05	7.20E-06	0.9300	3E-04	0.000
Biomass	1.39E+00	-1.61E-02	6.11E-03	6.54E-05	-1.56E-04	2.77E-05	0.9300	3E-04	0.004
YEa	2.00E+00	-2.32E-02	8.82E-03	9.44E-05	-2.25E-04	3.99E-05	0.9300	3E-04	0.008
aceA	1.42E+01	-1.74E-01	6.77E-02	7.57E-04	-1.62E-03	8.85E-05	0.9593	4E-05	0.238
aceB	1.42E+01	-1.74E-01	6.77E-02	7.57E-04	-1.62E-03	8.85E-05	0.9593	4E-05	0.238
ackA	-2.10E+01	2.80E-01	-1.51E-01	-1.25E-03	2.99E-03	-7.47E-05	0.9727	1E-05	0.325
acn	1.33E+01	-1.64E-01	6.38E-02	7.16E-04	-1.52E-03	7.10E-05	0.9611	4E-05	0.202
acs	1.38E+00	-1.98E-02	1.60E-02	8.35E-05	-2.75E-04	3.25E-05	0.9668	2E-05	0.003
actPin	2.08E+01	-2.56E-01	9.99E-02	1.12E-03	-2.38E-03	1.11E-04	0.9611	4E-05	0.495
actPout	2.27E+01	-2.77E-01	3.68E-01	1.17E-03	-5.42E-03	2.56E-04	0.9396	2E-04	1.991
aroG	4.26E+00	-5.25E-02	1.79E-02	2.28E-04	-4.62E-04	3.17E-05	0.9639	3E-05	0.020
atpABCD	5.48E+01	-6.81E-01	2.01E-02	2.84E-03	-3.92E-03	1.08E-03	0.9770	6E-06	2.323
cyo	8.63E+01	-1.05E+00	9.37E-01	4.52E-03	-1.56E-02	7.58E-04	0.9290	3E-04	17.449
dadA	1.24E+00	-1.44E-02	5.47E-03	5.85E-05	-1.39E-04	2.47E-05	0.9300	3E-04	0.003
eda	2.42E+00	1.60E-02	2.11E-01	-4.25E-05	-2.15E-03	-3.58E-04	0.8903	1E-03	0.890
edd	2.42E+00	1.60E-02	2.11E-01	-4.25E-05	-2.15E-03	-3.58E-04	0.8903	1E-03	0.890
eno	1.80E+01	-2.65E-01	1.44E-01	1.08E-03	-2.98E-03	6.25E-04	0.9675	2E-05	0.422
fba	9.55E+00	-1.62E-01	-2.65E-02	6.56E-04	-6.05E-04	5.10E-04	0.9491	9E-05	0.176
fum	1.42E+01	-1.74E-01	6.77E-02	7.57E-04	-1.62E-03	8.85E-05	0.9593	4E-05	0.238
galP	1.37E+01	-1.67E-01	1.92E-01	7.05E-04	-2.96E-03	1.67E-04	0.9347	2E-04	0.626
gapA	1.85E+01	-2.71E-01	1.46E-01	1.11E-03	-3.05E-03	6.36E-04	0.9672	2E-05	0.444
gdhA	6.20E-01	-7.18E-03	2.73E-03	2.92E-05	-6.96E-05	1.23E-05	0.9300	3E-04	0.001
glk	1.37E+01	-1.67E-01	1.92E-01	7.05E-04	-2.96E-03	1.67E-04	0.9347	2E-04	0.626
gltA	1.33E+01	-1.64E-01	6.38E-02	7.16E-04	-1.52E-03	7.10E-05	0.9611	4E-05	0.202
gnd	3.29E-02	4.87E-05	3.24E-03	3.47E-07	-3.37E-05	-5.20E-06	0.9244	4E-04	0.000
icdA	-8.80E-01	1.02E-02	-3.88E-03	-4.15E-05	9.88E-05	-1.75E-05	0.9300	3E-04	0.002
lpdA	6.50E+00	-5.37E-02	-2.89E-02	2.06E-04	-2.21E-05	7.96E-05	0.9317	3E-04	0.165
maeA	1.92E+01	-3.22E-01	-3.77E-02	1.31E-03	-1.36E-03	9.91E-04	0.9520	8E-05	0.639
maeB	6.62E+00	-8.14E-02	3.18E-02	3.56E-04	-7.56E-04	3.53E-05	0.9612	4E-05	0.050
mdh	2.60E+00	5.56E-02	1.41E-01	-1.48E-04	-1.12E-03	-8.49E-04	0.5396	1E-01	1.723
ndh	1.71E+01	-2.89E-01	-4.73E-02	1.17E-03	-1.08E-03	9.11E-04	0.9491	9E-05	0.560
nuo	2.96E+01	-2.57E-01	4.77E-01	1.15E-03	-6.42E-03	-5.17E-04	0.8078	8E-03	7.452
pckA	7.12E+00	-8.53E-02	2.91E-02	3.71E-04	-7.52E-04	4.17E-05	0.9578	5E-05	0.065
pfk	9.55E+00	-1.62E-01	-2.65E-02	6.56E-04	-6.05E-04	5.10E-04	0.9491	9E-05	0.176
pgi	1.12E+01	-1.82E-01	-2.16E-02	7.43E-04	-7.64E-04	5.29E-04	0.9638	3E-05	0.162
pgk	1.85E+01	-2.71E-01	1.46E-01	1.11E-03	-3.05E-03	6.36E-04	0.9672	2E-05	0.444
pgl	2.45E+00	1.60E-02	2.14E-01	-4.22E-05	-2.18E-03	-3.63E-04	0.8908	1E-03	0.912
pgm	1.80E+01	-2.65E-01	1.44E-01	1.08E-03	-2.98E-03	6.25E-04	0.9675	2E-05	0.422
pntA	1.92E+01	1.24E-02	1.83E+00	2.63E-04	-1.92E-02	-2.89E-03	0.9234	4E-04	43.741
pntB	2.06E+01	-4.73E-02	1.63E+00	4.72E-04	-1.74E-02	-2.40E-03	0.9247	4E-04	33.849
poxB	2.42E+01	-3.20E-01	4.34E-01	1.38E-03	-6.31E-03	2.52E-04	0.9550	6E-05	1.876
ppc	1.85E+01	-3.12E-01	-4.55E-02	1.27E-03	-1.23E-03	9.75E-04	0.9502	9E-05	0.632
ppsA	-6.11E-04	1.07E-05	8.89E-06	-5.33E-08	-1.48E-07	2.04E-13	0.7071	3E-02	0.000
pta	-2.10E+01	2.80E-01	-1.51E-01	-1.25E-03	2.99E-03	-7.47E-05	0.9727	1E-05	0.325
pykA	2.30E+00	1.55E-02	2.00E-01	-4.18E-05	-2.04E-03	-3.41E-04	0.8896	1E-03	0.810
rpe	-1.62E+00	2.01E-02	-4.77E-03	-8.62E-05	1.56E-04	-1.84E-05	0.9700	1E-05	0.002
rpi	1.65E+00	-2.00E-02	7.98E-03	8.65E-05	-1.90E-04	1.33E-05	0.9539	7E-05	0.004
sdh	1.54E+01	-1.88E-01	7.32E-02	8.16E-04	-1.76E-03	1.13E-04	0.9570	5E-05	0.294
sdh	1.42E+01	-1.74E-01	6.77E-02	7.57E-04	-1.62E-03	8.85E-05	0.9593	4E-05	0.238
sucAB	-7.89E-04	2.13E-05	-1.11E-05	-1.07E-07	1.48E-07	-1.86E-13	0.8944	1E-03	0.000
sucCD	-7.89E-04	2.13E-05	-1.11E-05	-1.07E-07	1.48E-07	-1.86E-13	0.8944	1E-03	0.000
talA	1.32E+00	-1.62E-02	6.54E-03	7.10E-05	-1.53E-04	6.67E-06	0.9598	4E-05	0.002
tktA1	1.32E+00	-1.62E-02	6.54E-03	7.10E-05	-1.53E-04	6.67E-06	0.9598	4E-05	0.002
tktA2	2.94E+00	-3.63E-02	1.13E-02	1.57E-04	-3.09E-04	2.51E-05	0.9657	2E-05	0.009
tpiA	9.55E+00	-1.62E-01	-2.65E-02	6.56E-04	-6.05E-04	5.10E-04	0.9491	9E-05	0.176
zwf	2.45E+00	1.60E-02	2.14E-01	-4.22E-05	-2.18E-03	-3.63E-04	0.8908	1E-03	0.912

Table 8 Dynamic Flux Model Response Surfaces parameter values for Mid Exponential point

	a	b	c	d	e	f	R2	pval	SSE
Maint	-8.24E-01	4.97E-02	-8.57E-02	-2.65E-04	1.09E-03	8.30E-05	0.9639	3E-05	0.0292
Biomass P	2.06E-01	-3.70E-03	6.29E-03	1.91E-05	-8.93E-05	-1.37E-05	0.9246	4E-04	0.0003
Biomass	7.80E-01	-1.40E-02	2.39E-02	7.24E-05	-3.39E-04	-5.19E-05	0.9243	4E-04	0.0043
YEa	1.13E+00	-2.02E-02	3.45E-02	1.05E-04	-4.90E-04	-7.49E-05	0.9243	4E-04	0.0090
aceA	9.72E+00	-1.69E-01	2.76E-01	9.02E-04	-3.69E-03	-7.96E-04	0.9452	1E-04	0.4514
aceB	9.72E+00	-1.69E-01	2.76E-01	9.02E-04	-3.69E-03	-7.96E-04	0.9452	1E-04	0.4514
ackA	-1.46E+01	2.66E-01	-4.41E-01	-1.42E-03	5.84E-03	1.22E-03	0.9555	6E-05	0.7964
acn	9.19E+00	-1.59E-01	2.60E-01	8.50E-04	-3.46E-03	-7.61E-04	0.9469	1E-04	0.3886
acs	6.70E-01	-1.50E-02	2.71E-02	7.63E-05	-3.87E-04	-3.63E-05	0.9506	8E-05	0.0025
actPin	1.44E+01	-2.50E-01	4.09E-01	1.34E-03	-5.45E-03	-1.19E-03	0.9462	1E-04	0.9736
actPout	9.92E+00	-2.45E-01	6.49E-01	1.35E-03	-8.71E-03	-1.20E-03	0.9054	8E-04	2.2965
aroG	2.89E+00	-4.93E-02	7.83E-02	2.62E-04	-1.05E-03	-2.27E-04	0.9500	9E-05	0.0350
atpABCD	3.57E+01	-5.75E-01	6.87E-01	2.90E-03	-1.01E-02	-1.83E-03	0.9605	4E-05	3.4814
cyo	4.75E+01	-9.66E-01	2.09E+00	5.24E-03	-2.80E-02	-4.69E-03	0.9223	4E-04	22.8413
dadA	6.99E-01	-1.25E-02	2.14E-02	6.49E-05	-3.04E-04	-4.64E-05	0.9244	4E-04	0.0035
eda	-1.78E-01	-3.44E-02	3.41E-01	3.21E-04	-4.04E-03	-8.98E-04	0.8337	5E-03	1.0200
edd	-1.78E-01	-3.44E-02	3.41E-01	3.21E-04	-4.04E-03	-8.98E-04	0.8337	5E-03	1.0200
eno	8.31E+00	-1.78E-01	2.54E-01	8.52E-04	-3.98E-03	-1.31E-04	0.9694	2E-05	0.1755
fba	5.42E+00	-9.23E-02	-1.17E-02	3.72E-04	-4.05E-04	2.96E-04	0.9405	2E-04	0.0695
fum	9.68E+00	-1.68E-01	2.75E-01	8.96E-04	-3.68E-03	-7.94E-04	0.9458	1E-04	0.4415
galP	6.36E+00	-1.46E-01	3.62E-01	7.94E-04	-4.88E-03	-6.92E-04	0.9100	7E-04	0.7040
gapA	8.61E+00	-1.84E-01	2.64E-01	8.80E-04	-4.11E-03	-1.52E-04	0.9682	2E-05	0.1950
gdhA	3.48E-01	-6.25E-03	1.07E-02	3.23E-05	-1.51E-04	-2.32E-05	0.9246	4E-04	0.0009
glk	6.36E+00	-1.46E-01	3.62E-01	7.94E-04	-4.88E-03	-6.92E-04	0.9100	7E-04	0.7040
gltA	9.19E+00	-1.59E-01	2.60E-01	8.50E-04	-3.46E-03	-7.61E-04	0.9469	1E-04	0.3886
gnd	-8.03E-02	1.86E-03	2.19E-03	-7.95E-06	-1.92E-05	-7.00E-06	0.9586	5E-05	0.0000
icdA	-5.35E-01	1.01E-02	-1.64E-02	-5.25E-05	2.34E-04	3.55E-05	0.9110	6E-04	0.0024
lpdA	4.45E+00	-6.05E-02	9.16E-02	3.24E-04	-1.26E-03	-3.55E-04	0.9175	5E-04	0.1845
maeA	1.08E+01	-1.89E-01	1.82E-03	7.77E-04	-1.11E-03	5.26E-04	0.9543	6E-05	0.2084
maeB	4.60E+00	-7.96E-02	1.30E-01	4.26E-04	-1.73E-03	-3.80E-04	0.9461	1E-04	0.0985
mdh	3.96E+00	-6.81E-02	4.20E-01	5.95E-04	-4.53E-03	-1.74E-03	0.8491	4E-03	1.9161
ndh	9.76E+00	-1.67E-01	-1.97E-02	6.76E-04	-7.41E-04	5.24E-04	0.9432	1E-04	0.2122
nuo	1.66E+01	-3.43E-01	1.11E+00	2.10E-03	-1.38E-02	-3.11E-03	0.8772	2E-03	9.1633
pckA	4.93E+00	-8.42E-02	1.37E-01	4.51E-04	-1.83E-03	-4.07E-04	0.9435	1E-04	0.1209
pfk	5.42E+00	-9.23E-02	-1.17E-02	3.72E-04	-4.05E-04	2.96E-04	0.9405	2E-04	0.0695
pgi	6.57E+00	-1.12E-01	1.72E-02	4.78E-04	-8.03E-04	2.16E-04	0.9722	1E-05	0.0464
pgk	8.61E+00	-1.84E-01	2.64E-01	8.80E-04	-4.11E-03	-1.52E-04	0.9682	2E-05	0.1950
pgl	-2.58E-01	-3.25E-02	3.43E-01	3.13E-04	-4.06E-03	-9.05E-04	0.8358	5E-03	1.0153
pgm	8.31E+00	-1.78E-01	2.54E-01	8.52E-04	-3.98E-03	-1.31E-04	0.9694	2E-05	0.1755
pntA	-9.14E-01	-3.29E-01	2.74E+00	2.80E-03	-3.25E-02	-6.93E-03	0.8612	3E-03	53.9907
pntB	1.28E+00	-3.32E-01	2.45E+00	2.66E-03	-2.93E-02	-6.10E-03	0.8634	3E-03	42.4312
poxB	1.07E+01	-2.76E-01	7.08E-01	1.51E-03	-9.49E-03	-1.26E-03	0.9208	4E-04	2.2479
ppc	1.05E+01	-1.81E-01	-1.19E-02	7.39E-04	-9.13E-04	5.45E-04	0.9477	1E-04	0.2264
ppsA	8.06E-10	-1.58E-11	-3.48E-12	8.24E-14	8.88E-16	4.88E-15			0.0000
pta	-1.46E+01	2.66E-01	-4.41E-01	-1.42E-03	5.84E-03	1.22E-03	0.9555	6E-05	0.7964
pykA	-2.15E-01	-3.14E-02	3.24E-01	2.98E-04	-3.83E-03	-8.53E-04	0.8343	5E-03	0.9129
rpe	-1.14E+00	1.99E-02	-2.84E-02	-1.04E-04	3.91E-04	7.92E-05	0.9457	1E-04	0.0056
rpi	1.06E+00	-1.80E-02	3.06E-02	9.59E-05	-4.10E-04	-8.61E-05	0.9482	1E-04	0.0051
sdh	1.04E+01	-1.80E-01	2.96E-01	9.61E-04	-3.98E-03	-8.40E-04	0.9445	1E-04	0.5221
sdh	9.68E+00	-1.68E-01	2.75E-01	8.96E-04	-3.68E-03	-7.94E-04	0.9458	1E-04	0.4415
sucAB	-3.97E-02	1.23E-03	-1.31E-03	-6.48E-06	1.89E-05	2.67E-06	0.7538	2E-02	0.0001
sucCD	-3.97E-02	1.23E-03	-1.31E-03	-6.48E-06	1.89E-05	2.67E-06	0.7538	2E-02	0.0001
talA	8.77E-01	-1.47E-02	2.49E-02	7.89E-05	-3.30E-04	-7.39E-05	0.9526	7E-05	0.0031
tktA1	8.77E-01	-1.47E-02	2.49E-02	7.89E-05	-3.30E-04	-7.39E-05	0.9526	7E-05	0.0031
tktA2	2.02E+00	-3.46E-02	5.33E-02	1.83E-04	-7.22E-04	-1.53E-04	0.9488	1E-04	0.0172
tpiA	5.42E+00	-9.23E-02	-1.17E-02	3.72E-04	-4.05E-04	2.96E-04	0.9405	2E-04	0.0695
zwf	-2.58E-01	-3.25E-02	3.43E-01	3.13E-04	-4.06E-03	-9.05E-04	0.8358	5E-03	1.0153

Table 9 Dynamic Flux Model Response Surfaces parameter values for Mid Stationary point

	a	b	c	d	e	f	R2	pval	SSE
Maint	-3.30E+01	5.60E-01	1.02E+00	-2.64E-03	-7.09E-03	-4.33E-03	0.7329	2E-02	79.9998
Biomass P	-5.36E-03	1.05E-04	3.89E-05	-4.27E-07	1.70E-06	-1.00E-06	0.9805	3E-06	0.0000
Biomass	-2.00E-02	3.85E-04	1.60E-04	-1.55E-06	6.37E-06	-3.87E-06	0.9727	1E-05	0.0000
YEa	-2.80E-02	5.37E-04	2.33E-04	-2.13E-06	9.19E-06	-5.60E-06	0.9730	1E-05	0.0000
aceA	1.51E+00	-6.69E-03	-6.74E-02	4.15E-05	4.94E-04	2.47E-04	0.8530	3E-03	0.2219
aceB	1.51E+00	-6.69E-03	-6.74E-02	4.15E-05	4.94E-04	2.47E-04	0.8530	3E-03	0.2219
ackA	6.02E-01	-1.18E-02	-3.43E-03	4.99E-05	-1.72E-04	9.33E-05	0.9673	2E-05	0.0027
acn	-9.47E-01	3.49E-02	8.84E-03	-1.55E-04	-4.01E-05	-7.37E-05	0.2656	5E-01	0.4491
acs	3.80E+00	-2.88E-02	-1.39E-01	1.48E-04	7.62E-04	6.18E-04	0.8791	2E-03	0.8943
actPin	-6.01E-01	1.18E-02	3.44E-03	-4.97E-05	1.73E-04	-9.41E-05	0.9675	2E-05	0.0027
actPout	2.23E+00	-1.80E-02	-7.74E-02	9.55E-05	4.55E-04	3.31E-04	0.8705	2E-03	0.2985
aroG	-2.22E+00	4.07E-02	6.58E-02	-1.90E-04	-4.47E-04	-2.86E-04	0.6858	4E-02	0.4149
atpABCD	-2.37E+01	4.81E-01	6.61E-01	-2.23E-03	-4.64E-03	-2.90E-03	0.5922	9E-02	60.7247
cyo	-8.65E+00	3.09E-01	1.50E-01	-1.38E-03	-1.53E-03	-6.87E-04	0.2431	5E-01	41.3889
dadA	-1.77E-02	3.39E-04	1.52E-04	-1.36E-06	5.56E-06	-3.47E-06	0.9726	1E-05	0.0000
eda	2.25E+00	-1.80E-02	-7.93E-02	9.32E-05	4.39E-04	3.52E-04	0.8767	2E-03	0.2991
edd	2.25E+00	-1.80E-02	-7.93E-02	9.32E-05	4.39E-04	3.52E-04	0.8767	2E-03	0.2991
eno	-2.60E+00	6.41E-02	7.32E-02	-2.94E-04	-6.39E-04	-2.89E-04	0.3764	3E-01	1.5047
fba	-2.45E+00	4.16E-02	7.66E-02	-1.96E-04	-5.31E-04	-3.26E-04	0.7347	2E-02	0.4463
fum	-9.59E-01	3.52E-02	8.94E-03	-1.56E-04	-3.61E-05	-7.61E-05	0.2649	5E-01	0.4497
galP	-2.38E+00	6.36E-02	6.25E-02	-2.90E-04	-5.50E-04	-2.52E-04	0.3177	4E-01	1.5204
gapA	-2.60E+00	6.42E-02	7.33E-02	-2.95E-04	-6.37E-04	-2.91E-04	0.3776	3E-01	1.5054
gdhA	-8.08E-03	1.57E-04	6.44E-05	-6.13E-07	2.96E-06	-1.73E-06	0.9758	7E-06	0.0000
glk	-2.38E+00	6.36E-02	6.25E-02	-2.90E-04	-5.50E-04	-2.52E-04	0.3177	4E-01	1.5204
gltA	-9.47E-01	3.49E-02	8.84E-03	-1.55E-04	-4.01E-05	-7.37E-05	0.2656	5E-01	0.4491
gnd	-4.30E+00	7.90E-02	1.29E-01	-3.71E-04	-9.33E-04	-5.44E-04	0.6693	5E-02	1.6542
icdA	-2.45E+00	4.16E-02	7.62E-02	-1.97E-04	-5.34E-04	-3.21E-04	0.7313	3E-02	0.4468
lpdA	-2.66E+00	4.56E-02	7.74E-02	-2.13E-04	-4.74E-04	-3.55E-04	0.7525	2E-02	0.4568
maeA	2.03E-02	-5.40E-04	7.68E-04	3.17E-06	3.70E-07	-6.73E-06	0.9829	2E-06	0.0000
maeB	3.61E+00	-2.52E-02	-1.38E-01	1.33E-04	8.15E-04	5.90E-04	0.8737	2E-03	0.8917
mdh	-3.09E+00	5.42E-02	7.90E-02	-2.50E-04	-3.57E-04	-4.12E-04	0.7850	1E-02	0.4820
ndh	9.38E+00	-7.44E-02	-3.34E-01	3.80E-04	1.78E-03	1.51E-03	0.8800	2E-03	5.2007
nuo	-2.31E+01	3.94E-01	6.92E-01	-1.85E-03	-4.50E-03	-3.06E-03	0.7438	2E-02	36.5822
pckA	-2.06E-01	4.05E-03	1.19E-03	-1.70E-05	5.92E-05	-3.23E-05	0.9676	2E-05	0.0003
pfk	-2.45E+00	4.16E-02	7.66E-02	-1.96E-04	-5.31E-04	-3.26E-04	0.7347	2E-02	0.4463
pgi	-3.31E-01	2.56E-03	1.24E-02	-1.24E-05	-5.66E-05	-5.97E-05	0.8959	1E-03	0.0063
pgk	-2.60E+00	6.42E-02	7.33E-02	-2.95E-04	-6.37E-04	-2.91E-04	0.3776	3E-01	1.5054
pgl	-2.05E+00	6.10E-02	5.01E-02	-2.77E-04	-4.94E-04	-1.92E-04	0.2554	5E-01	1.5458
pgm	-2.60E+00	6.41E-02	7.32E-02	-2.94E-04	-6.39E-04	-2.89E-04	0.3764	3E-01	1.5047
pntA	-3.24E+00	1.20E-01	5.91E-02	-5.32E-04	-5.82E-04	-2.83E-04	0.2444	5E-01	6.5013
pntB	-3.55E-01	5.54E-03	8.05E-03	-1.54E-05	1.44E-04	-1.17E-04	0.9300	3E-04	0.0075
poxB	6.03E+00	-4.68E-02	-2.17E-01	2.43E-04	1.22E-03	9.49E-04	0.8762	2E-03	2.2223
ppc	1.93E+00	-1.51E-02	-6.89E-02	7.75E-05	3.79E-04	3.04E-04	0.8772	2E-03	0.2248
ppsA	8.06E-10	-1.58E-11	-3.48E-12	8.24E-14	8.88E-16	4.88E-15			0.0000
pta	6.02E-01	-1.18E-02	-3.43E-03	4.99E-05	-1.72E-04	9.33E-05	0.9673	2E-05	0.0027
pykA	-2.51E+00	4.25E-02	7.75E-02	-1.99E-04	-5.12E-04	-3.39E-04	0.7451	2E-02	0.4442
rpe	-2.12E+00	3.90E-02	6.43E-02	-1.83E-04	-4.74E-04	-2.66E-04	0.6631	5E-02	0.4131
rpi	-2.18E+00	4.00E-02	6.51E-02	-1.87E-04	-4.59E-04	-2.77E-04	0.6755	5E-02	0.4141
sdh	-9.76E-01	3.55E-02	9.08E-03	-1.57E-04	-3.04E-05	-7.95E-05	0.2641	5E-01	0.4504
sdh	-9.59E-01	3.52E-02	8.94E-03	-1.56E-04	-3.61E-05	-7.61E-05	0.2649	5E-01	0.4497
sucAB	-2.46E+00	4.19E-02	7.63E-02	-1.98E-04	-5.30E-04	-3.23E-04	0.7329	2E-02	0.4472
sucCD	-2.46E+00	4.19E-02	7.63E-02	-1.98E-04	-5.30E-04	-3.23E-04	0.7329	2E-02	0.4472
talA	-2.17E+00	3.99E-02	6.50E-02	-1.87E-04	-4.61E-04	-2.76E-04	0.6747	5E-02	0.4139
tktA1	-2.17E+00	3.99E-02	6.50E-02	-1.87E-04	-4.61E-04	-2.76E-04	0.6747	5E-02	0.4139
tktA2	-5.03E-02	8.79E-04	7.57E-04	-3.33E-06	1.39E-05	-9.87E-06	0.9399	2E-04	0.0001
tpiA	-2.45E+00	4.16E-02	7.66E-02	-1.96E-04	-5.31E-04	-3.26E-04	0.7347	2E-02	0.4463
zwf	-2.05E+00	6.10E-02	5.01E-02	-2.77E-04	-4.94E-04	-1.92E-04	0.2554	5E-01	1.5458

Table 10 Dynamic Flux Model Response Surfaces critical points for Initial Exponential time point

	X	Y	Z	H	D	STAT
Maint	98.52	38.16	0.07	-1.4E-06	-5.4E-04	Saddle Point
Biomass Precursor	116.62	29.94	0.14	-2.9E-09	3.4E-05	Saddle Point
Biomass	116.58	29.95	0.54	-4.2E-08	1.3E-04	Saddle Point
YEa	116.58	29.95	0.78	-8.7E-08	1.9E-04	Saddle Point
aceA	113.37	24.02	5.15	-4.9E-06	1.5E-03	Saddle Point
aceB	113.37	24.02	5.15	-4.9E-06	1.5E-03	Saddle Point
ackA	111.34	26.53	-7.41	-1.5E-05	-2.5E-03	Saddle Point
acn	113.14	23.65	4.81	-4.4E-06	1.4E-03	Saddle Point
acs	111.63	35.54	0.56	-9.3E-08	1.7E-04	Saddle Point
actPin	113.13	23.65	7.52	-1.1E-05	2.2E-03	Saddle Point
actPout	114.27	36.65	13.58	-2.5E-05	2.3E-03	Saddle Point
aroG	113.50	23.22	1.49	-4.2E-07	4.6E-04	Saddle Point
atpABCD	116.51	18.58	15.32	-4.6E-05	5.7E-03	Saddle Point
cyo	113.82	32.88	41.67	-2.8E-04	9.0E-03	Saddle Point
dadA	116.59	29.95	0.49	-3.3E-08	1.2E-04	Saddle Point
eda	N.A.	N.A.	N.A.	N.A.	N.A.	
edd	N.A.	N.A.	N.A.	N.A.	N.A.	
eno	111.71	35.76	5.78	-1.3E-05	2.2E-03	Saddle Point
fba	113.34	25.88	0.04	-1.8E-06	1.3E-03	Saddle Point
fum	113.37	24.02	5.15	-4.9E-06	1.5E-03	Saddle Point
galP	114.32	35.78	7.61	-8.4E-06	1.4E-03	Saddle Point
gapA	111.83	35.65	5.99	-1.4E-05	2.2E-03	Saddle Point
gdhA	116.57	29.96	0.24	-8.3E-09	5.8E-05	Saddle Point
glk	114.32	35.78	7.61	-8.4E-06	1.4E-03	Saddle Point
gltA	113.14	23.65	4.81	-4.4E-06	1.4E-03	Saddle Point
gnd	N.A.	N.A.	N.A.	N.A.	N.A.	
icdA	116.55	29.95	-0.34	-1.7E-08	-8.3E-05	Saddle Point
lpdA	N.A.	N.A.	N.A.	N.A.	N.A.	
maeA	112.83	27.24	0.49	-8.1E-06	2.6E-03	Saddle Point
maeB	113.13	23.65	2.39	-1.1E-06	7.1E-04	Saddle Point
mdh	N.A.	N.A.	N.A.	N.A.	N.A.	
ndh	113.33	25.88	0.07	-5.9E-06	2.3E-03	Saddle Point
nuo	118.50	32.37	22.09	-3.0E-05	2.3E-03	Saddle Point
pckA	113.78	22.50	2.59	-1.1E-06	7.4E-04	Saddle Point
pfk	113.34	25.88	0.04	-1.8E-06	1.3E-03	Saddle Point
pgi	113.64	25.19	0.57	-2.6E-06	1.5E-03	Saddle Point
pgk	111.83	35.65	5.99	-1.4E-05	2.2E-03	Saddle Point
pgl	N.A.	N.A.	N.A.	N.A.	N.A.	
pgm	111.71	35.76	5.78	-1.3E-05	2.2E-03	Saddle Point
pntA	N.A.	N.A.	N.A.	N.A.	N.A.	
pntB	N.A.	N.A.	N.A.	N.A.	N.A.	
poxB	112.62	36.68	14.13	-3.5E-05	2.8E-03	Saddle Point
ppc	113.12	26.43	0.23	-7.2E-06	2.5E-03	Saddle Point
ppsA	100.00	30.00	0.00	3.2E-14	-1.1E-07	Maximum
pta	111.34	26.53	-7.41	-1.5E-05	-2.5E-03	Saddle Point
pykA	N.A.	N.A.	N.A.	N.A.	N.A.	
rpe	114.19	21.98	-0.52	-5.4E-08	-1.7E-04	Saddle Point
rpi	113.89	25.02	0.61	-6.6E-08	1.7E-04	Saddle Point
sdh	113.66	24.48	5.63	-5.7E-06	1.6E-03	Saddle Point
sdh	113.37	24.02	5.15	-4.9E-06	1.5E-03	Saddle Point
sucAB	100.00	37.50	0.00	-6.3E-14	-2.1E-07	Saddle Point
sucCD	100.00	37.50	0.00	-6.3E-14	-2.1E-07	Saddle Point
talA	113.14	23.86	0.48	-4.3E-08	1.4E-04	Saddle Point
tktA1	113.14	23.86	0.48	-4.3E-08	1.4E-04	Saddle Point
tktA2	113.69	22.91	1.01	-2.0E-07	3.1E-04	Saddle Point
tpiA	113.34	25.88	0.04	-1.8E-06	1.3E-03	Saddle Point
zwf	N.A.	N.A.	N.A.	N.A.	N.A.	

Table 11 **Dynamic Flux Model Response Surfaces critical points for Mid Exponential time point**

	X	Y	Z	H	D	STATUS
Maint	99.45	35.67	0.12	-1.2E-06	-5.3E-04	SaddlePoint
Biomass Precursor	106.39	27.06	0.09	-7.0E-09	3.8E-05	SaddlePoint
Biomass	106.38	27.06	0.36	-1.0E-07	1.4E-04	SaddlePoint
YEa	106.38	27.06	0.52	-2.1E-07	2.1E-04	SaddlePoint
aceA	105.10	26.08	4.45	-1.4E-05	1.8E-03	SaddlePoint
aceB	105.10	26.08	4.45	-1.4E-05	1.8E-03	SaddlePoint
ackA	104.91	26.84	-6.54	-3.5E-05	-2.8E-03	SaddlePoint
acn	105.06	26.01	4.23	-1.2E-05	1.7E-03	SaddlePoint
acs	105.48	29.98	0.28	-1.2E-07	1.5E-04	SaddlePoint
actPin	105.04	26.03	6.61	-3.1E-05	2.7E-03	SaddlePoint
actPout	104.24	30.08	6.90	-4.8E-05	2.7E-03	SaddlePoint
aroG	105.35	25.84	1.31	-1.2E-06	5.2E-04	SaddlePoint
atpABCD	106.93	24.44	13.37	-1.2E-04	5.8E-03	SaddlePoint
cyo	104.89	28.56	26.66	-6.1E-04	1.0E-02	SaddlePoint
dadA	106.38	27.06	0.32	-8.1E-08	1.3E-04	SaddlePoint
eda	97.55	31.39	3.50	-6.0E-06	6.4E-04	SaddlePoint
edd	97.55	31.39	3.50	-6.0E-06	6.4E-04	SaddlePoint
eno	107.10	30.16	2.58	-1.4E-05	1.7E-03	SaddlePoint
fba	113.28	26.94	0.03	-6.9E-07	7.4E-04	SaddlePoint
fum	105.13	26.07	4.45	-1.4E-05	1.8E-03	SaddlePoint
galP	104.65	29.65	4.09	-1.6E-05	1.6E-03	SaddlePoint
gapA	107.11	30.06	2.72	-1.5E-05	1.8E-03	SaddlePoint
gdhA	106.37	27.06	0.16	-2.0E-08	6.5E-05	SaddlePoint
glk	104.65	29.65	4.09	-1.6E-05	1.6E-03	SaddlePoint
gltA	105.06	26.01	4.23	-1.2E-05	1.7E-03	SaddlePoint
gnd	100.21	38.85	0.06	5.6E-10	-1.6E-05	Maximum
icdA	105.63	27.13	-0.22	-5.0E-08	-1.0E-04	SaddlePoint
lpdA	105.19	21.52	2.25	-1.8E-06	6.5E-04	SaddlePoint
maeA	112.26	27.45	0.28	-3.7E-06	1.6E-03	SaddlePoint
maeB	105.00	26.01	2.10	-3.1E-06	8.5E-04	SaddlePoint
mdh	97.54	27.64	6.44	-1.4E-05	1.2E-03	SaddlePoint
ndh	113.08	26.74	0.05	-2.3E-06	1.4E-03	SaddlePoint
nuo	102.92	28.49	14.70	-1.3E-04	4.2E-03	SaddlePoint
pckA	105.07	25.77	2.27	-3.5E-06	9.0E-04	SaddlePoint
pfk	113.28	26.94	0.03	-6.9E-07	7.4E-04	SaddlePoint
pgi	111.92	25.72	0.50	-1.6E-06	9.6E-04	SaddlePoint
pgk	107.11	30.06	2.72	-1.5E-05	1.8E-03	SaddlePoint
pgl	97.46	31.44	3.55	-5.9E-06	6.3E-04	SaddlePoint
pgm	107.10	30.16	2.58	-1.4E-05	1.7E-03	SaddlePoint
pntA	97.96	31.73	26.46	-4.1E-04	5.6E-03	SaddlePoint
pntB	98.54	31.61	23.68	-3.5E-04	5.3E-03	SaddlePoint
poxB	104.07	30.42	7.14	-5.9E-05	3.0E-03	SaddlePoint
ppc	112.75	27.10	0.14	-3.0E-06	1.5E-03	SaddlePoint
ppsA	N.A.	N.A.	N.A.	N.A.	N.A.	N.A.
pta	104.91	26.84	-6.54	-3.5E-05	-2.8E-03	SaddlePoint
pykA	97.52	31.41	3.34	-5.3E-06	6.0E-04	SaddlePoint
rpe	105.37	25.62	-0.46	-1.7E-07	-2.1E-04	SaddlePoint
rpi	105.56	26.17	0.51	-1.6E-07	1.9E-04	SaddlePoint
sdh	105.23	26.14	4.78	-1.6E-05	1.9E-03	SaddlePoint
sdh	105.13	26.07	4.45	-1.4E-05	1.8E-03	SaddlePoint
sucAB	100.29	27.66	0.00	-5.0E-10	-1.3E-05	SaddlePoint
sucCD	100.29	27.66	0.00	-5.0E-10	-1.3E-05	SaddlePoint
talA	105.35	25.97	0.43	-1.1E-07	1.6E-04	SaddlePoint
tktA1	105.35	25.97	0.43	-1.1E-07	1.6E-04	SaddlePoint
tktA2	105.35	25.78	0.88	-5.5E-07	3.7E-04	SaddlePoint
tpiA	113.28	26.94	0.03	-6.9E-07	7.4E-04	SaddlePoint
zwf	97.46	31.44	3.55	-5.9E-06	6.3E-04	SaddlePoint

Table 12 Dynamic Flux Model Response Surfaces critical points for Mid Stationary time point

	X	Y	Z	H	D	STATUS
Maint	N.A.	N.A.	N.A.	N.A.	N.A.	N.A.
Biomass P	101.2195138	18.29268088	0.00	-3.9E-12	-8.5E-07	Saddle Point
Biomass	101.6896182	18.30347797	0.00	-5.4E-11	-3.1E-06	Saddle Point
YEa	101.8557854	18.34797328	0.00	-1.1E-10	-4.3E-06	Saddle Point
aceA	N.A.	N.A.	N.A.	N.A.	N.A.	N.A.
aceB	N.A.	N.A.	N.A.	N.A.	N.A.	N.A.
ackA	102.0297453	17.74535786	-0.03	-4.3E-08	1.0E-04	Saddle Point
acn	N.A.	N.A.	N.A.	N.A.	N.A.	N.A.
acs	N.A.	N.A.	N.A.	N.A.	N.A.	N.A.
actPin	102.0142513	17.82987939	0.03	-4.3E-08	-9.9E-05	Saddle Point
actPout	N.A.	N.A.	N.A.	N.A.	N.A.	N.A.
aroG	N.A.	N.A.	N.A.	N.A.	N.A.	N.A.
atpABCD	N.A.	N.A.	N.A.	N.A.	N.A.	N.A.
cyo	105.103926	25.55564988	9.50	8.0E-06	-2.8E-03	Maximum
dadA	101.7536473	18.04713822	0.00	-4.2E-11	-2.7E-06	Saddle Point
eda	N.A.	N.A.	N.A.	N.A.	N.A.	N.A.
edd	N.A.	N.A.	N.A.	N.A.	N.A.	N.A.
eno	90.82887713	36.71046191	1.66	6.7E-07	-5.9E-04	Maximum
fba	N.A.	N.A.	N.A.	N.A.	N.A.	N.A.
fum	N.A.	N.A.	N.A.	N.A.	N.A.	N.A.
galP	94.39187781	35.14130059	1.72	5.7E-07	-5.8E-04	Maximum
gapA	90.74991582	36.81328411	1.66	6.7E-07	-5.9E-04	Maximum
gdhA	101.2401474	18.73774204	0.00	-1.0E-11	-1.2E-06	Saddle Point
glk	94.39187781	35.14130059	1.72	5.7E-07	-5.8E-04	Maximum
gltA	N.A.	N.A.	N.A.	N.A.	N.A.	N.A.
gnd	N.A.	N.A.	N.A.	N.A.	N.A.	N.A.
icdA	N.A.	N.A.	N.A.	N.A.	N.A.	N.A.
lpdA	N.A.	N.A.	N.A.	N.A.	N.A.	N.A.
maeA	117.3747398	30.43638656	0.00	-4.1E-11	6.3E-06	Saddle Point
maeB	N.A.	N.A.	N.A.	N.A.	N.A.	N.A.
mdh	N.A.	N.A.	N.A.	N.A.	N.A.	N.A.
ndh	N.A.	N.A.	N.A.	N.A.	N.A.	N.A.
nuo	N.A.	N.A.	N.A.	N.A.	N.A.	N.A.
pckA	102.0215625	17.85194739	0.01	-5.1E-09	-3.4E-05	Saddle Point
pfk	N.A.	N.A.	N.A.	N.A.	N.A.	N.A.
pgi	N.A.	N.A.	N.A.	N.A.	N.A.	N.A.
pgk	90.74991582	36.81328411	1.66	6.7E-07	-5.9E-04	Maximum
pgl	99.05067118	31.43545467	1.76	5.1E-07	-5.5E-04	Maximum
pgm	90.82887713	36.71046191	1.66	6.7E-07	-5.9E-04	Maximum
pntA	105.9433197	25.06627442	3.84	1.2E-06	-1.1E-03	Maximum
pntB	112.6823372	17.68623652	0.03	-2.2E-08	-3.1E-05	Saddle Point
poxB	N.A.	N.A.	N.A.	N.A.	N.A.	N.A.
ppc	N.A.	N.A.	N.A.	N.A.	N.A.	N.A.
ppsA	N.A.	N.A.	N.A.	N.A.	N.A.	N.A.
pta	102.0297453	17.74535786	-0.03	-4.3E-08	1.0E-04	Saddle Point
pykA	N.A.	N.A.	N.A.	N.A.	N.A.	N.A.
rpe	N.A.	N.A.	N.A.	N.A.	N.A.	N.A.
rpi	N.A.	N.A.	N.A.	N.A.	N.A.	N.A.
sdh	N.A.	N.A.	N.A.	N.A.	N.A.	N.A.
sdh	N.A.	N.A.	N.A.	N.A.	N.A.	N.A.
sucAB	N.A.	N.A.	N.A.	N.A.	N.A.	N.A.
sucCD	N.A.	N.A.	N.A.	N.A.	N.A.	N.A.
talA	N.A.	N.A.	N.A.	N.A.	N.A.	N.A.
tktA1	N.A.	N.A.	N.A.	N.A.	N.A.	N.A.
tktA2	N.A.	N.A.	N.A.	N.A.	N.A.	N.A.
tpiA	N.A.	N.A.	N.A.	N.A.	N.A.	N.A.
zwf	99.05067118	31.43545467	1.76	5.1E-07	-5.5E-04	Maximum
