# Supplementary material for: Metabolic modeling and response surface analysis of an Escherichia coli strain engineered for shikimic acid production
Source: BMC Syst Biol. 2018 Nov 12;12:102. doi: 10.1186/s12918-018-0632-4 (PMC6233605; doi:10.1186/s12918-018-0632-4)
Supplement: Supplementary file 2 — Metabolic network definition for dynamic model. Description: Definition of reactions, internal and external metabolites comprehending the metabolic network. Matlab program section for the genetic algorithm used for parameter approximation. (PDF 173 kb) [file 12918_2018_632_MOESM2_ESM.pdf]

SUPPLEMENTARY MATERIAL 2

Metabolic modeling and response surface analysis for an engineered Escherichia coli for shikimic acid production

Juan A. Martínez, Alberto Rodriguez, Fabian Moreno, Noemí Flores, Alvaro R. Lara, Octavio T. Ramírez, Guillermo Gosset and Francisco Bolivar

Metabolic Network definition for Dynamic model:

Reaction definitions:

EMP Pathway

GalP : GLC + HEXT = GLCi

Glk : GLCi + ATP = G6P

Pgi : G6P = F6P

Pfk : F6P + ATP = FDP

Fba : FDP = T3P1 + T3P2

TpiA : T3P2 = T3P1

GapA : T3P1 = NADH + 13PDG

Pgk : 13PDG = 3PG + ATP

Pgm : 3PG = 2PG

Eno : 2PG = PEP

PykA : PEP = PYR + ATP

PpsA : PYR + 2 ATP = PEP

LpdA : PYR = ACCOA + NADH

Pentose Phosphate Pathwway

G6Pdh : G6P = D6PGL + NADPH

Pgl : D6PGL = D6PGC

Gnd : D6PGC = NADPH + RL5P

Rpi : RL5P = R5P

Rpe : RL5P = X5P

TktA1 : R5P + X5P = T3P1 + S7P

TktA2 : F6P + T3P1 = X5P + E4P

TalA : T3P1 + S7P = E4P + F6P

PGdh : D6PGC = 2KD6PG

KDPGa : 2KD6PG = T3P1 + PYR

Tricarboxylic Acid Cylce

Csyn : ACCOA + OA = CIT

Acn : CIT = ICIT

IcdA : ICIT = NADPH + AKG

KGdh : AKG = NADH + SUCCOA

SucCD : SUCCOA = ATP + SUCC

SdhABCD : SUCC = FADH + FUM

Fum : FUM = MAL

Mdh : MAL = NADH + OA

Pyruvate Metabolism

Pta : ACCOA = ACTP

AckA : ACTP = ATP + ACin

Acs : 2 ATP + ACin = ACCOA

ActPin : AC = ACin

ActPout : ACin = AC

Anaplerotic Reactions

PckA : OA + ATP = PEP

Ppc : PEP = OA

MaeB : MAL = NADPH + PYR

MaeA : MAL = NADH + PYR

Icl : ICIT = GLX + SUCC

Msn : ACCOA + GLX = MAL

Respiration and energetics ———————————————

Ndh : NADH = QH2

Nuo : NADH = QH2 + 3.5 HEXT

PoxB : PYR = ACin + QH2

Cyo : QH2 = 2 HEXT

Sdh : FADH = QH2

PntA : NADPH = NADH

PntB : NADH + 2 HEXT = NADPH

AtpABCD : 4 HEXT = ATP

Top YE Amino Acid Biosynthesis & Degraddation ———————————————

YEa : YE + HEXT = 0.51 BIOMp + 0.25 AAA + 0.63 GLU + 0.62 ALA

Gdh : AKG + NADPH = GLU

DadA : ALA = PYR + FADH

Aromatic Amino Acids Biosythesis ———————————————

DAHPs : E4P + PEP = DAHP

DHQd : DAHP = DHQ

DHSs : DHQ = DHS

SAdh : DHS + NADPH = SA

Biomass ———————————————

BIOMSp : 1.496 3PG + 3.7478 ACCOA + 59.8100 ATP + 0.0709 F6P + 0.1290 T3P1 + 0.2050 G6P + 5.1464 GLU + 13.0279 NADPH + 1.7867 OA + 0.1581 PEP + 2.8328 PYR + 0.8977 R5P = BIOMp + 4.1 AKG + 3.5 NADH BIOMS : BIOMp + 0.3610 AAA = BIOM

ATPdrain : ATP = MAINT

-Reversible reactions:

Pgi : Phosphoglucose isomerase

Fba : Fructose-1,6-biphosphate aldolase

Tpi : Triosphophate Isomerase

GapA : Glyceraldehyde-3-phosphate dehydrogenase-A

Pgk : Phosphoglycerate kinase

Pgm : Phosphoglycerate mutase

Eno : Enolase

G6Pdh : Glucose 6-phosphate-1-dehydrogenase

Rpi : Ribose-5-phosphate isomerase

Rpe : Ribulose phosphate 3-epimerase

TktA1 : Transketolase I 1

TktA2 : Transketolase I 2

Tal : Transaldolase

Acn : Aconitase

Icd : Isocitrate dehydrogenase

SucCD : Succinyl-CoA synthetase

Fum : Fumarase

Mdh : Malate dehydrogenase

Pta : Phosphotransacetylase Ack : Acetate kinase

Sdh : Succinate dehydrogenase complex FADH reaction

ATPase : F0F1-ATPase

Gdh : Glutamate dehydrogenase

-Irreversible reactions:

GalP : Galactose-proton symporter

Glk : Glucokinase

Pfk : Phosphofructokinase

PykA : Pyruvate Kinase II

PpsA : Phosphoenolpyruvate synthase

LpdA : Pyruvate dehydrogenase

Pgl : 6-Phosphogluconolactonase

Gnd : 6-Phosphogluconate dehydrogenase (decarboxylating)

PGdh : Phosphogluconate dehydratase

KDPGa : 2-Keto-3-deoxy-6-phosphogluconate aldolase

Csyn : Citrate synthase

KGdh : 2-Ketoglutarate dehyrogenase

SdhABCD : Succinate dehydrogenase complex

Acs : Acetyl-CoA synthetase

ActPin : Acetate transport in

ActPout : Acetate transport out

Pck : Phosphoenolpyruvate carboxykinase

Ppc : Phosphoenolpyruvate carboxylase

MaeB : Malic enzyme (NADP)

MaeA : Malic enzyme (NAD)

Icl : Isocitrate lyase

Msn : Malate synthase A

Ndh : NADH dehydrogenase II

Nuo : NADH dehydrogenase I

PoxB : Pyruvate oxidase

cyo : Cytochrome oxidase

PntA : Pyridine nucleotide transhydrogenase A

PntB : Pyridine nucleotide transhydrogenase B

YEa : Yeast extract consumption reaction

DadA : Alanine D-Amino acid dehydrogenase

DAHPs : 2-Dehydro-3-deoxyphosphoheptonate aldolase G DHSs : 3-Dehydroquinate synthase DHQd : 3-Dehydroquinate dehydratase SAdh : Shikimate dehydrogenase

BIOMp : Biomass P

BIOM : Biomass

ATPdrain : Maintenance

-Internal metabolites:

13PDG : 1,3-Biosphosphoglyceric acid

2KD6PG : 2-keto-3-deoxy-6-phospho-gluconate

2PG : 2-Phosphoglycerate

3PG : 3-Phosphoglycerate

AAA : Aromatic amino acids (Phe, Tyr, Trp)

ACCOA : Acetyl-CoA

ACin : Acetic acid (internal)

ACTP: Acetyl-phosphate

AKG: α-ketoglutarate

ALA : Alanine

ATP : Adenosine triphosphate

BIOMp : Biomass precursor

CIT : Citrate

D6PGC : D-6-Phosphate-gluconate

D6PGL : D-6-Phosphate-glucono-delta-lactone

DAHP : 3-Deoxy-D-arabino-heptulosonic acid

DHQ : Dehydroquinate

DHS : Dehydroshikimate

E4P : Erythrose 4-phosphate

F6P : Fructose 6-phosphate

FADH : Flavin adenine dinucleotide reduced

FDP : Fructose 1,6-diphosphate

FUM : Fumarate

G6P: Glucose 6-phosphate

GLCi: Glucose (internal)

GLU : Glutamate

GLX : Glyoxylate

ICIT : Isocitrate

MAL : Malate

NADH : Nicotinamide adenine dinucleotide reduced

NADPH : Dihydronicotinamide adenine dinucleotide phosphate reduced

OA : Oxaloacetate

PEP : Phosphoenolpyruvate

PYR : Pyruvate

QH2 : Ubiquinol

R5P : Ribose 5-phosphate

RL5P : D-Ribulose 5-phosphate

S7P : sedo-Heptulose 7-phosphate

SUCC : Succinate

SUCCOA : Succinate-CoA

T3P1 : Glyceraldehyde 3-phosphate

T3P2: Dihydroxyacetone phosphate or glycerone phosphate

X5P : D-Xylulose-5-phosphate

HEXT : H^+ on periplasma

-External metabolites

AC : Acetic acid (External)

GLC : Glucose

SA : Shikimic Acid

YE : Yeast Extract

BIOM : Biomass

MAINT: Maintenance ATP drain

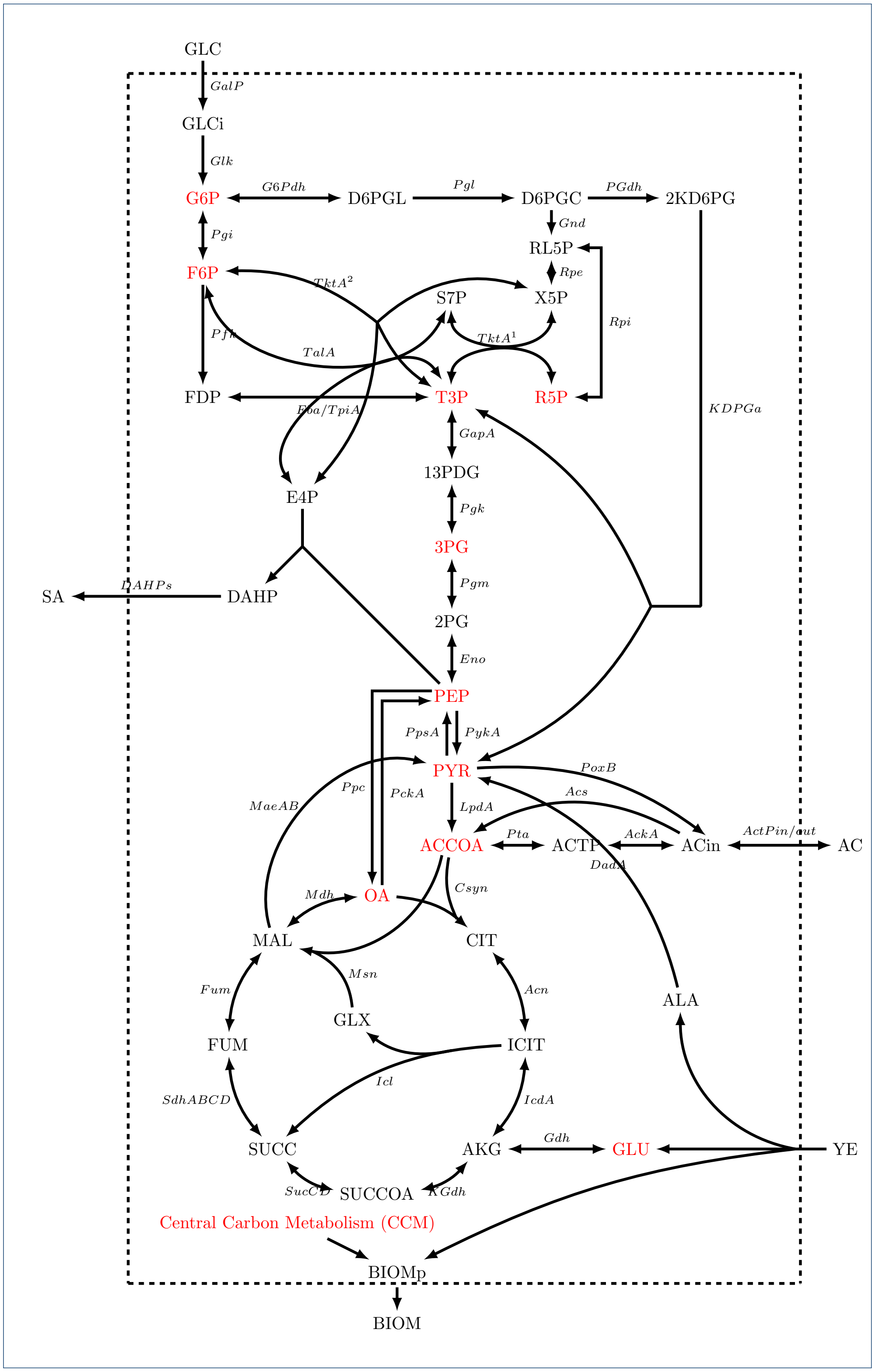
GLC
GalP
GLCi
Glk
G6P
G6Pdh
D6PGL
Pgl
D6PGC
PGdh
2KD6PG
Gnd
Pgi
RL5P
Rpe
F6P
TktA2
S7P
X5P
Rpi
Pfk
TalA
TktA1
FDP
T3P
R5P
Fba/TpiA
KDPGa
GapA
13PDG
E4P
Pgk
3PG
Pgm
SA
DAHPs
DAHP
2PG
Eno
PEP
PpsA
PykA
PYR
PoxB
MaeAB
Ppc
PckA
LpdA
Acs
ACCOA
Pta
ACTP
AckA
ACin
ActPin/out
AC
DadA
Mdh
OA
Csyn
MAL
CIT
Msn
Fum
Acn
ALA
GLX
FUM
ICIT
Icl
SdhABCD
IcdA
SUCC
AKG
Gdh
GLU
YE
SucCD
SUCCOA
KGdh
Central Carbon Metabolism (CCM)
BIOMp
BIOM

Matlab programming section for the Genetic algorithm

```
%%%i%%%% 1: Initiation remarks on equation variables and plotting
datafit.displayFrequency = 200;
kmax=kinetic.kmax(:,2);
kmax=cell2mat(kmax);
kmaxinitial=kmax;
K=kinetic.K(:,2);
K=cell2mat(K);
Kinitial=K;
%%%%%%%% 2: Initial Numeric SSE approximation 200 steps
[paraopt,ffval]=modelfitt(model,kinetic,cybernetic,datafit);
minimo(1)=ffval;
minimo(2)=Inf;
minimo2(1)=ffval;
Tkmax=zeros(numel(kmax),numel(kmax)+numel(K));
TK=zeros(numel(K),numel(kmax)+numel(K));
Tparaopt=zeros(numel(K)+numel(kmax),numel(K)+numel(kmax));
prompt = 'Max value for kmax area of study from 0 to n: ';
n=input(prompt);
% n=1;
prompt = 'Max value for kmax area of study from 0 to m: ';
m=input(prompt);
% m=10;
Log(1)=datetime('now');
z=3;
Evo=zeros(numel(K)+numel(kmax),numel(K)+numel(kmax));
C=2;
%%%%%%%% 3: First perturbation round to individual parameters
for ip=1:numel(paraopt)
       if ip<=numel(kmax)
           kinetic.kmax{ip,2}=n*abs(rand);
           Tkmax(:,ip)=cell2mat(kinetic.kmax(:,2));
           TK(:,ip)=cell2mat(kinetic.K(:,2));
```

```
        [paraopt,ffval]=modelfitt(model,kinetic,cybernetic,datafit);
        Tffval(ip)=ffval;
        Tparaopt(:,ip)=paraopt(:);
        kinetic.kmax(:,2)=num2cell(kmax(:));
        kinetic.K(:,2)=num2cell(K(:));
    else
        kinetic.K{(ip-numel(kmax)),2}=m*abs(rand);
        TK(:,ip)=cell2mat(kinetic.K(:,2));
        Tkmax(:,ip)=cell2mat(kinetic.kmax(:,2));
        [paraopt,ffval]=modelfitt(model,kinetic,cybernetic,datafit);
        Tffval(ip)=ffval;
        Tparaopt(:,ip)=paraopt(:);
        kinetic.kmax(:,2)=num2cell(kmax(:));
        kinetic.K(:,2)=num2cell(K(:));
    end
end
%%%%Minimum and second minimum SSE find
minimo(C)=min(Tffval);
x=find(Tffval==minimo(C));
Tffval2=sort(Tffval);
minimo2(C)=Tffval(2);
y=find(Tffval==minimo2(C));
%%%%Perturbed parameter crossing between 1st and 2nd minimum SSE daughters
Tparaopt(y,x)=Tparaopt(y,y);
Tparaopt(x,y)=Tparaopt(x,x);
Evo(:,C)=Tparaopt(:,x);
%%%%Construction of new Mother parameter set
kinetic.kmax(:,2)=num2cell(Tparaopt(1:numel(kmax),x));
kinetic.K(:,2)=num2cell(Tparaopt((numel(kmax)+1):(numel(kmax)+numel(K)),x));
Log(C)=datetime('now');
figure()
plot(Log,minimo,'--o')
pause(3)
```

```
    close all
%%%%%%%% 4: Consecutive generations of  numeric SSE approximation 200 steps

while (minimo(C)>=0.1)&&(abs(minimo(C-1)-minimo(C))>0.1)&&(C<25) % generations end point
    C=C+1;
    for ip=1:numel(paraopt)
       if ip<=numel(kmax)
           kinetic.kmax{ip,2}=n*abs(rand);
           Tkmax(:,ip)=cell2mat(kinetic.kmax(:,2));
           TK(:,ip)=cell2mat(kinetic.K(:,2));
           [paraopt,ffval]=modelfitt(model,kinetic,cybernetic,datafit);
           Tffval(ip)=ffval;
           Tparaopt(:,ip)=paraopt(:);
           kinetic.kmax(:,2)=num2cell(kmax(:));
           kinetic.K(:,2)=num2cell(K(:));
       else
           kinetic.K{(ip-numel(kmax)),2}=m*abs(rand);
           TK(:,ip)=cell2mat(kinetic.K(:,2));
           Tkmax(:,ip)=cell2mat(kinetic.kmax(:,2));
           [paraopt,ffval]=modelfitt(model,kinetic,cybernetic,datafit);
           Tffval(ip)=ffval;
           Tparaopt(:,ip)=paraopt(:);
           kinetic.kmax(:,2)=num2cell(kmax(:));
           kinetic.K(:,2)=num2cell(K(:));
       end
    end
  %%%%Minimum and second minimum SSE find
    minimo(C)=min(Tffval);
    x=find(Tffval==minimo(C));
    Tffval2=sort(Tffval);
    minimo2(C)=Tffval(2);
    y=find(Tffval==minimo2(C));
   %%%%Perturbed parameter crossing between 1st and 2nd minimum SSE daughters
```

```
        Tparaopt(y,x)=Tparaopt(y,y);
        Tparaopt(x,y)=Tparaopt(x,x);
        %%%%Construction of new Mother parameter set
        Evo(:,C)=Tparaopt(:,x);
        kinetic.kmax(:,2)=num2cell(Tparaopt(1:numel(kmax),x));
        kinetic.K(:,2)=num2cell(Tparaopt((numel(kmax)+1):(numel(kmax)+numel(K)),x));
        Log(C)=datetime('now');
        figure()
        plot(Log,minimo,'--o')
        pause('on')
        pause(3)
        pause('off')
    end
    %%%%%%%% 5: Final numeric SSE approximation
    paraopt = modelfit(model,kinetic,cybernetic,datafit);
         kinetic = updateParameters(kinetic,datafit.para,paraopt);
         [result,model] = simulate(model,initial,tspan,kinetic,cybernetic);
         figure()
         plot(Log,minimo,'--o')
```
