# Supplementary material for: Metabolic modeling and response surface analysis of an Escherichia coli strain engineered for shikimic acid production
Source: BMC Syst Biol. 2018 Nov 12;12:102. doi: 10.1186/s12918-018-0632-4 (PMC6233605; doi:10.1186/s12918-018-0632-4)
Supplement: Supplementary file 3 — Response surfaces and contour plots for all fluxes. Description: Response surfaces and contour plots for all reactions detailed on the metabolic network for initial exponential, mid exponential and mid stationary fermentation stages. (PDF 25,629 kb) [file 12918_2018_632_MOESM3_ESM.pdf]

SUPPLEMENTARY MATERIAL 3

# Metabolic modeling and response surface analysis for an engineered Escherichia coli for shikimic acid production

Juan A. Martínez, Alberto Rodriguez, Fabian Moreno, Noemí Flores, Alvaro R. Lara, Octavio T. Ramírez, Guillermo Gosset and Francisco Bolivar

Full list of author information is available at the end of the article

## List of Figures

1 EMP pathway calculated flux IEContours pt.1 . . . . . . . . . . . . . 4
2 EMP pathway calculated flux IEContours pt.2 . . . . . . . . . . . . . 5
3 PPP pathway calculated flux IEContours . . . . . . . . . . . . . . . . 6
4 TCA pathway calculated flux IEContours pt.1 . . . . . . . . . . . . . . 7
5 TCA pathway calculated flux IEContours pt.2 . . . . . . . . . . . . . . 8
6 PEP metabolism calculated flux IEContours . . . . . . . . . . . . . . . 9
7 PYR metabolism calculated flux IEContours . . . . . . . . . . . . . . 10
8 Anaplerotic genes calculated flux IEContours . . . . . . . . . . . . . . 11
9 Syntetic operon genes calculated flux IEContours . . . . . . . . . . . 12
10 EMP pathway calculated flux IESurfaces pt.1 . . . . . . . . . . . . . . 13
11 EMP pathway calculated flux IESurfaces pt.2 . . . . . . . . . . . . . . 14
12 PPP pathway calculated flux IESurfaces . . . . . . . . . . . . . . . . . 15
13 TCA pathway calculated flux IESurfaces pt.1 . . . . . . . . . . . . . . 16
14 TCA pathway calculated flux IESurfaces pt.2 . . . . . . . . . . . . . . 17
15 PEP metabolism calculated flux IESurfaces . . . . . . . . . . . . . . . 18
16 PYR metabolism calculated flux IESurfaces . . . . . . . . . . . . . . . 19
17 Anaplerotic genes calculated flux IESurfaces . . . . . . . . . . . . . . 20
18 Syntetic operon genes calculated flux IESurfaces . . . . . . . . . . . . 21
19 EMP pathway calculated flux MEContours pt.1 . . . . . . . . . . . . . 22

20 EMP pathway calculated flux MEContours pt.2 . . . . . . . . . . . . 23
21 PPP pathway calculated flux MEContours . . . . . . . . . . . . . . . 24
22 TCA pathway calculated flux MEContours pt.1 . . . . . . . . . . . . 25
23 TCA pathway calculated flux MEContours pt.2 . . . . . . . . . . . . 26
24 PEP metabolism calculated flux MEContours . . . . . . . . . . . . . 27
25 PYR metabolism calculated flux MEContours . . . . . . . . . . . . . 28
26 Anaplerotic genes calculated flux MEContours . . . . . . . . . . . . 29
27 Syntetic operon genes calculated flux MEContours . . . . . . . . . . 30
28 EMP pathway calculated flux MESurfaces pt.1 . . . . . . . . . . . . 31
29 EMP pathway calculated flux MESurfaces pt.2 . . . . . . . . . . . . 32
30 PPP pathway calculated flux MESurfaces . . . . . . . . . . . . . . . 33
31 TCA pathway calculated flux MESurfaces pt.1 . . . . . . . . . . . . 34
32 TCA pathway calculated flux MESurfaces pt.2 . . . . . . . . . . . . 35
33 PEP metabolism calculated flux MESurfaces . . . . . . . . . . . . . 36
34 PYR metabolism calculated flux MESurfaces . . . . . . . . . . . . . 37
35 Anaplerotic genes calculated flux MESurfaces . . . . . . . . . . . . 38
36 Syntetic operon genes calculated flux MESurfaces . . . . . . . . . . 39
37 EMP pathway calculated flux MSContours pt.1 . . . . . . . . . . . . 40
38 EMP pathway calculated flux MSContours pt.2 . . . . . . . . . . . . 41
39 PPP pathway calculated flux MSContours . . . . . . . . . . . . . . . 42
40 TCA pathway calculated flux MSContours pt.1 . . . . . . . . . . . . 43
41 TCA pathway calculated flux MSContours pt.2 . . . . . . . . . . . . 44
42 PEP metabolism calculated flux MSContours . . . . . . . . . . . . . 45
43 PYR metabolism calculated flux MSContours . . . . . . . . . . . . . 46
44 Anaplerotic genes calculated flux MSContours . . . . . . . . . . . . 47
45 Syntetic operon genes calculated flux MSContours . . . . . . . . . . 48
46 EMP pathway calculated flux MSSurfaces pt.1 . . . . . . . . . . . . 49
47 EMP pathway calculated flux MSSurfaces pt.2 . . . . . . . . . . . . 50
48 PPP pathway calculated flux MSSurfaces . . . . . . . . . . . . . . . 51
49 TCA pathway calculated flux MSSurfaces pt.1 . . . . . . . . . . . . 52
50 TCA pathway calculated flux MSSurfaces pt.2 . . . . . . . . . . . . 53
51 PEP metabolism calculated flux MSSurfaces . . . . . . . . . . . . . 54
52 PYR metabolism calculated flux MSSurfaces . . . . . . . . . . . . . 55

53 Anaplerotic genes calculated flux MSSurfaces . . . . . . . . . . . . . . 56

54 Syntetic operon genes calculated flux MSSurfaces . . . . . . . . . . . 57

# Initial Exponential Flux Response Surfaces

(a) galP

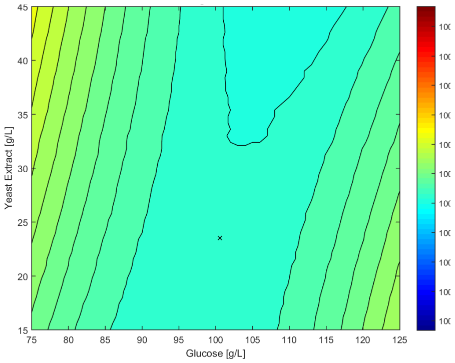


(b) glk

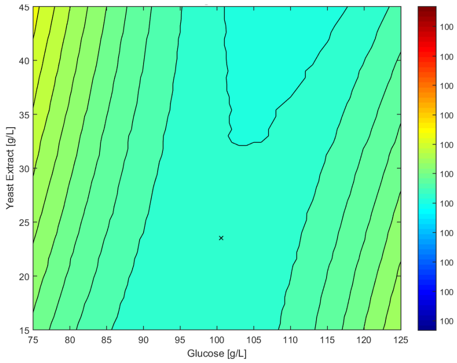


(c) pgi

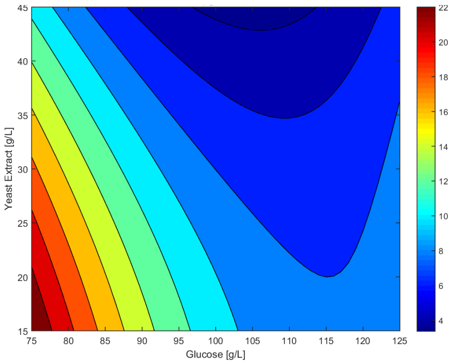


(d) pfk

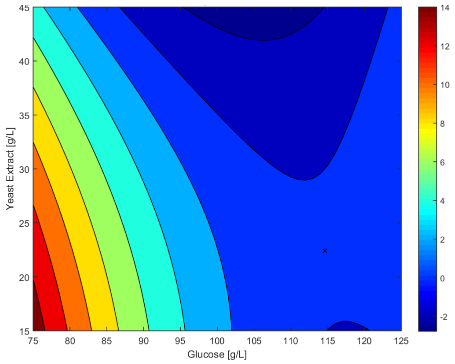


(e) fba

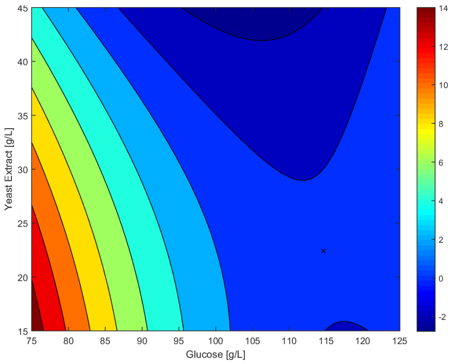


(f) gapA

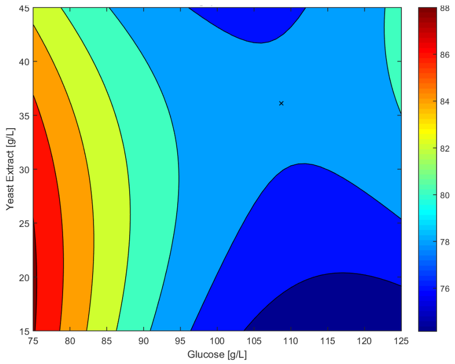


Figure 1: EMP pathway calculated flux IEContours pt.1

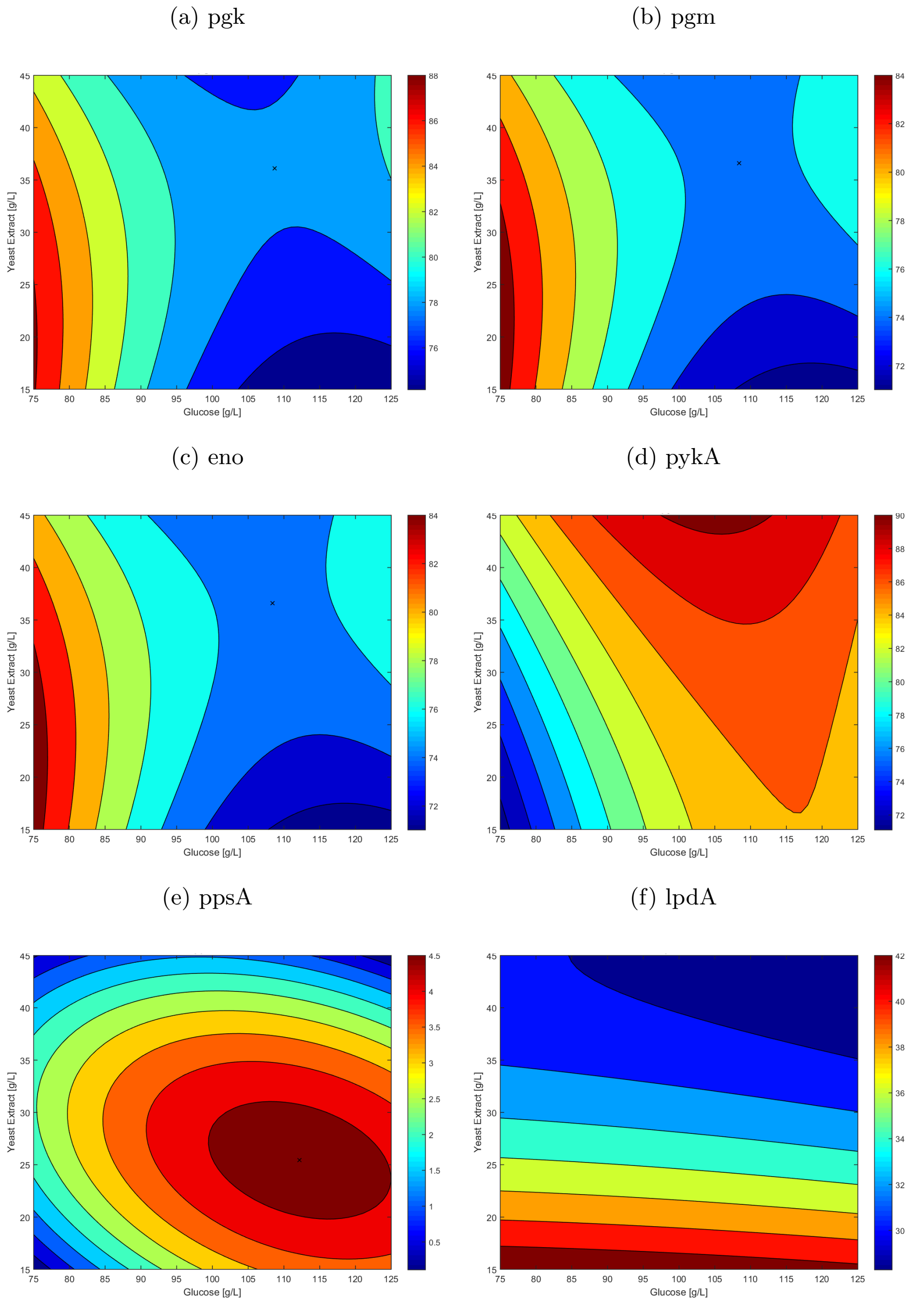


Figure 2: EMP pathway calculated flux IEContours pt.2

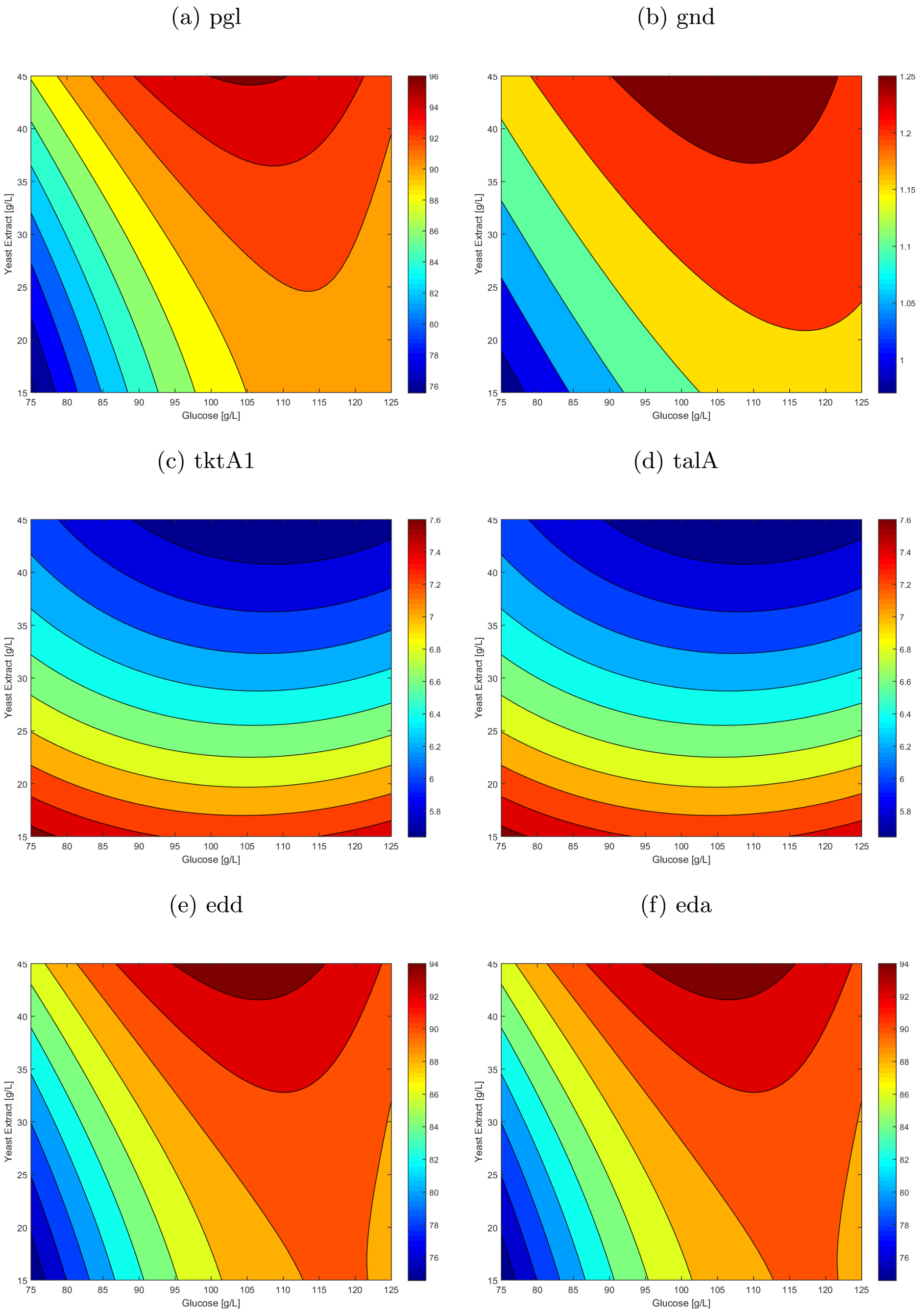


Figure 3: PPP pathway calculated flux IEContours

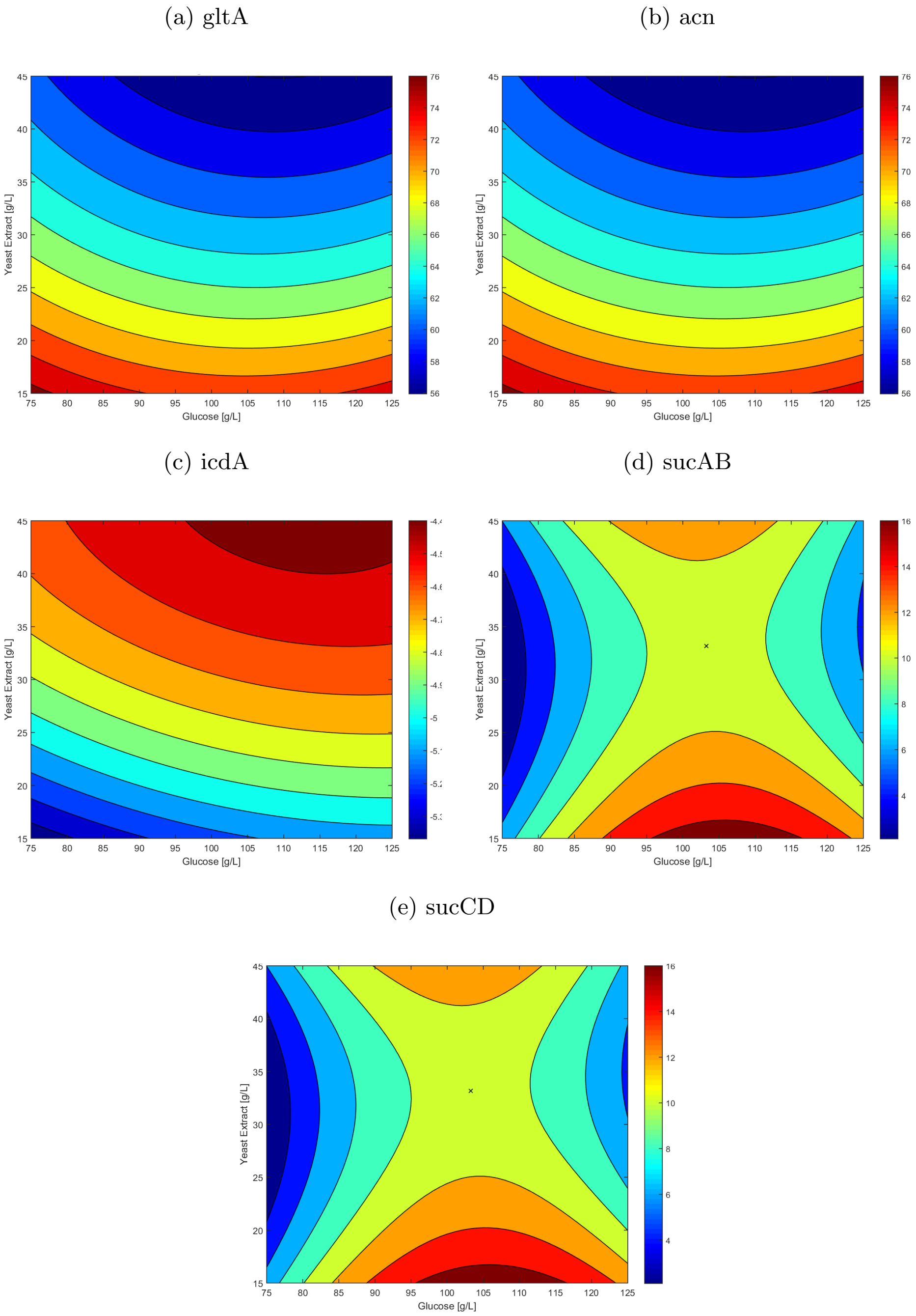


(a) gltA

(b) acn

(c) icdA

(d) sucAB

(e) sucCD

Figure 4: TCA pathway calculated flux IEContours pt.1

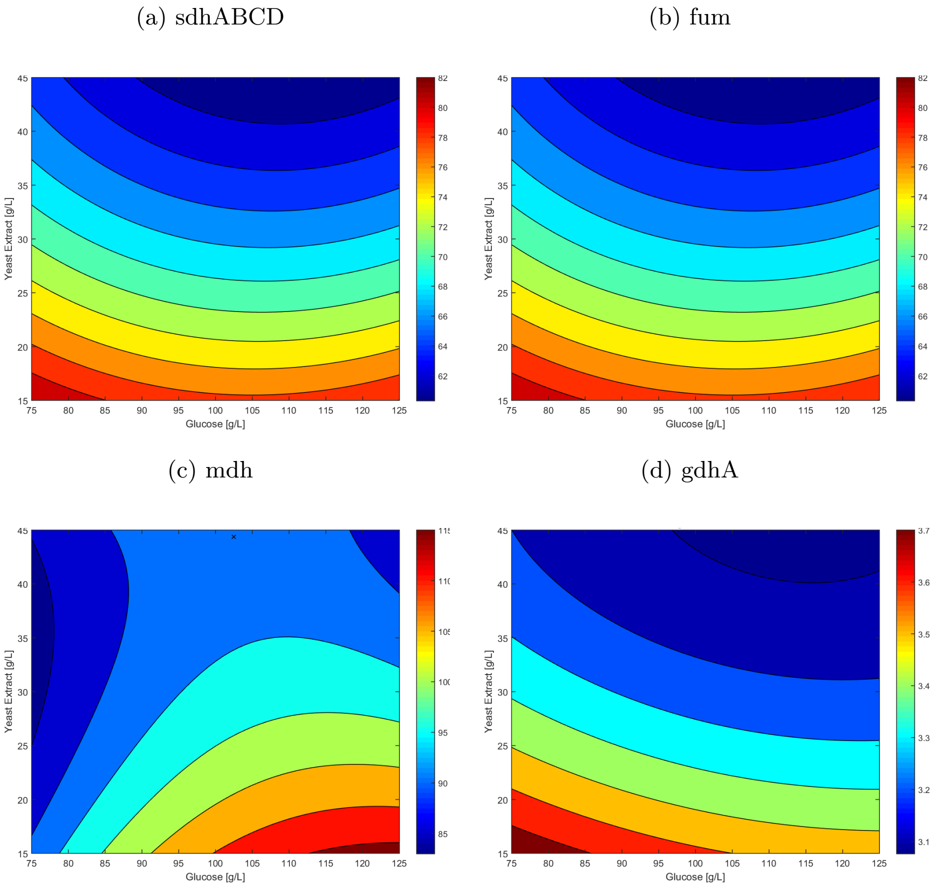


Figure 5: TCA pathway calculated flux IEContours pt.2

(a) eno (b) ppsA (c) pckA (d) pykA (e) ppc (f) aroG

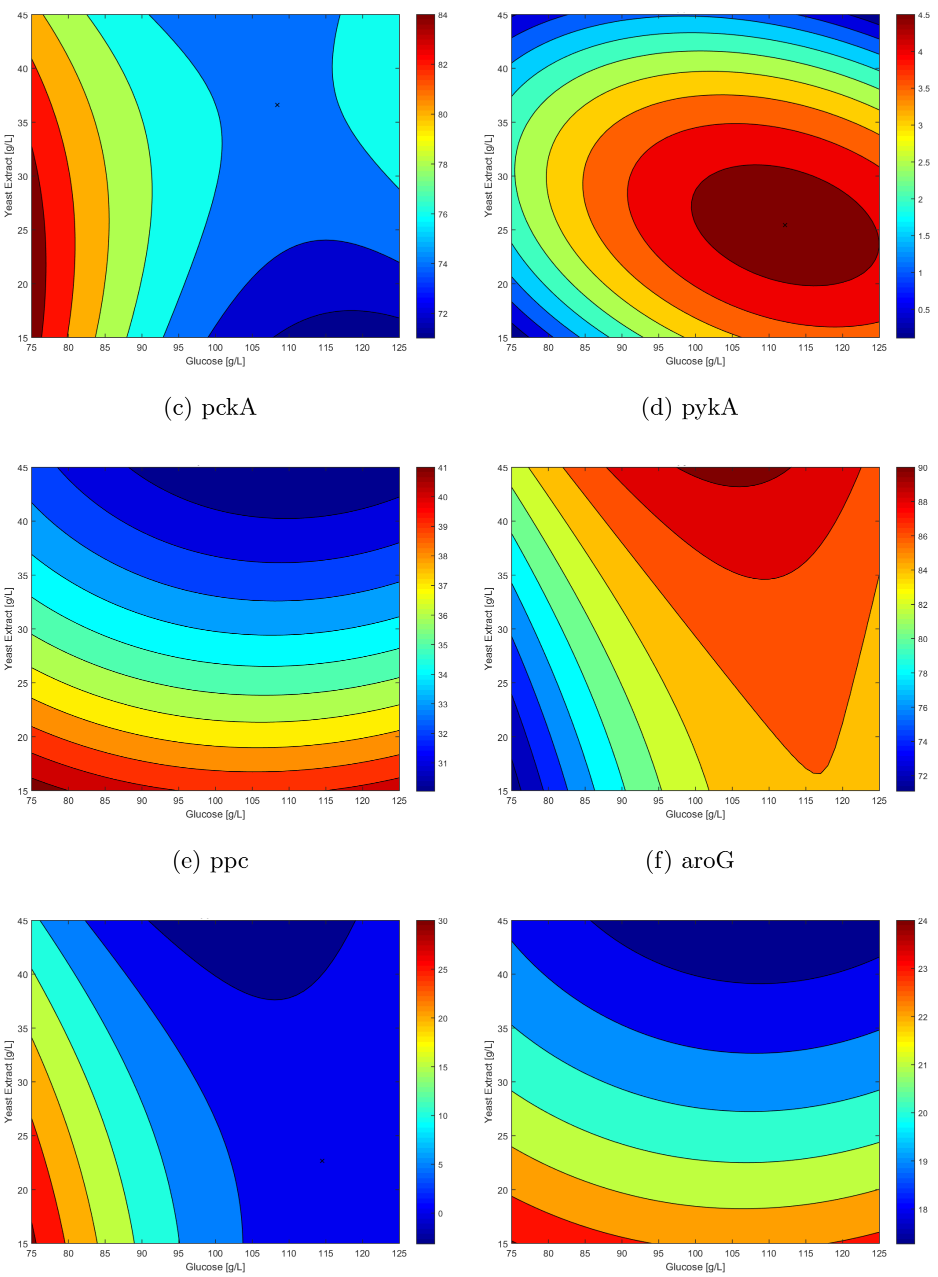


Figure 6: PEP metabolism calculated flux IEContours

(a) pta

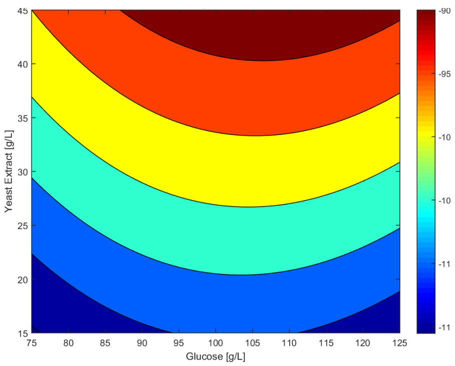


(b) ackA

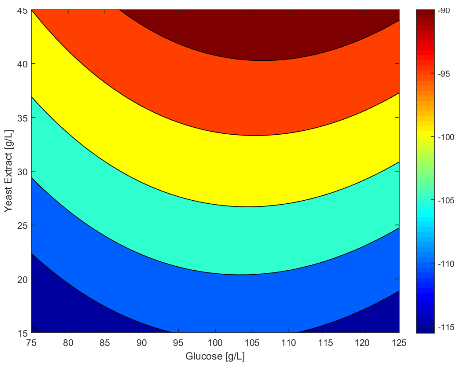


(c) acs

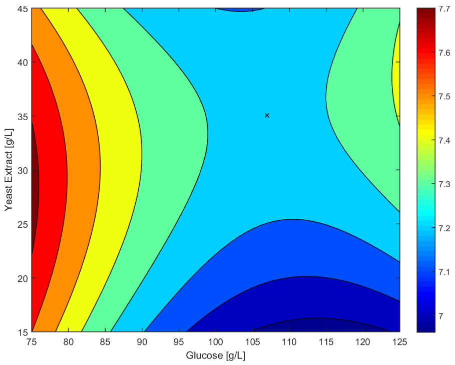


(d) poxB

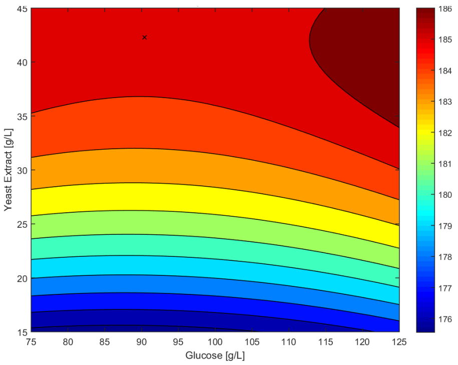


(e) actPout

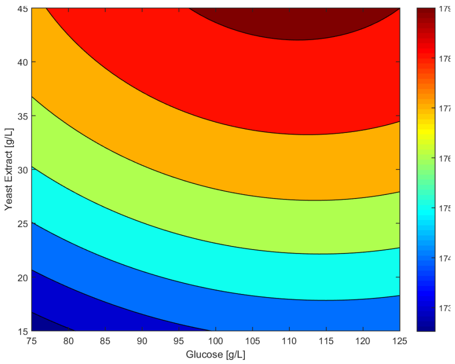


(f) actPin

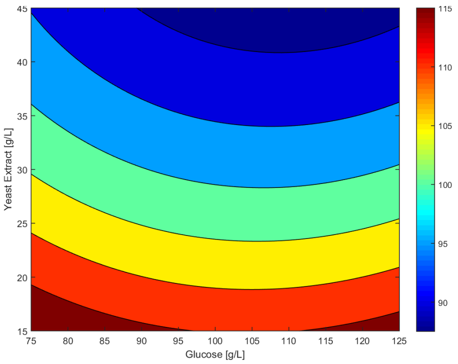


Figure 7: PYR metabolism calculated flux IEContours

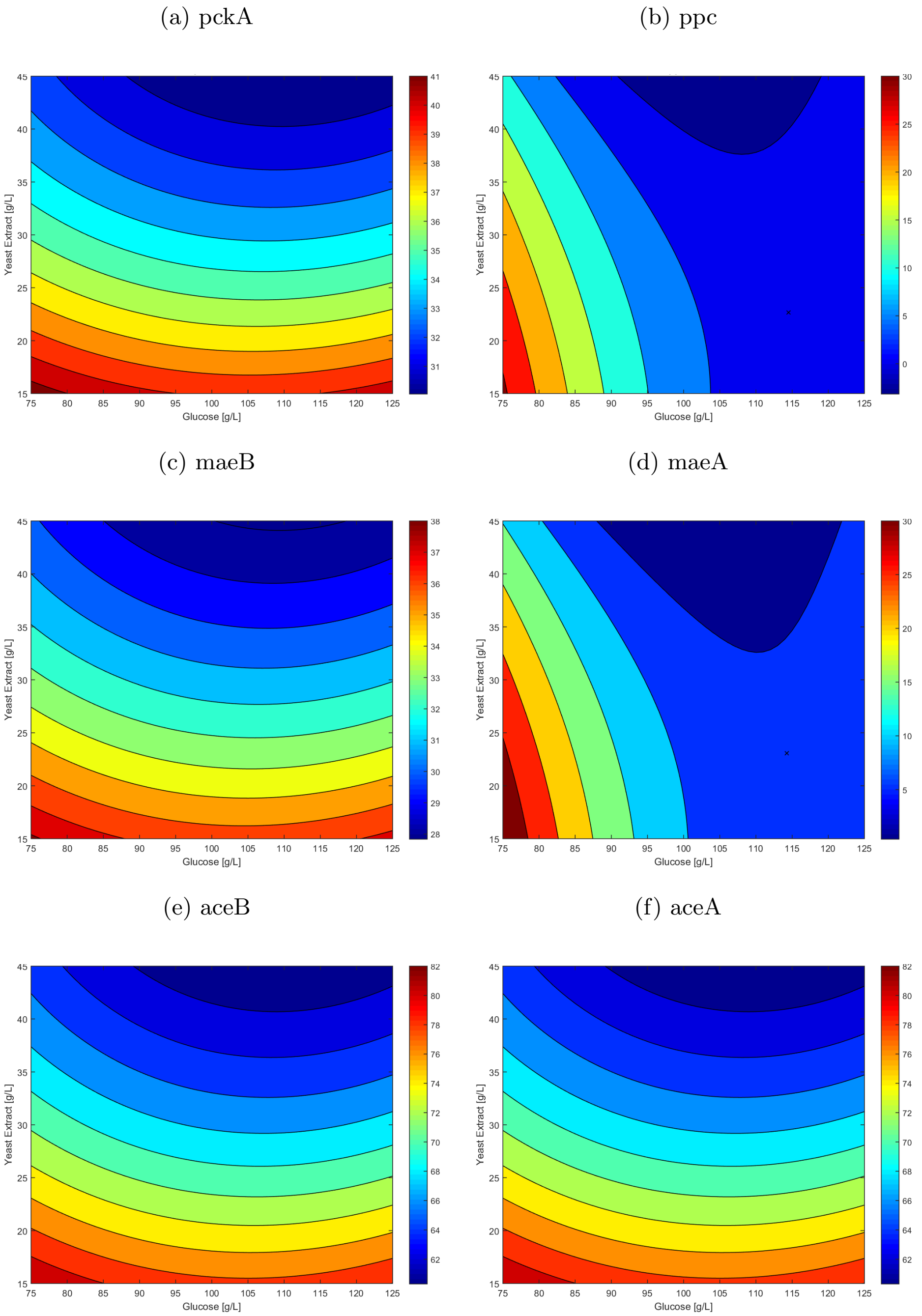


Figure 8: Anaplerotic genes calculated flux IEContours

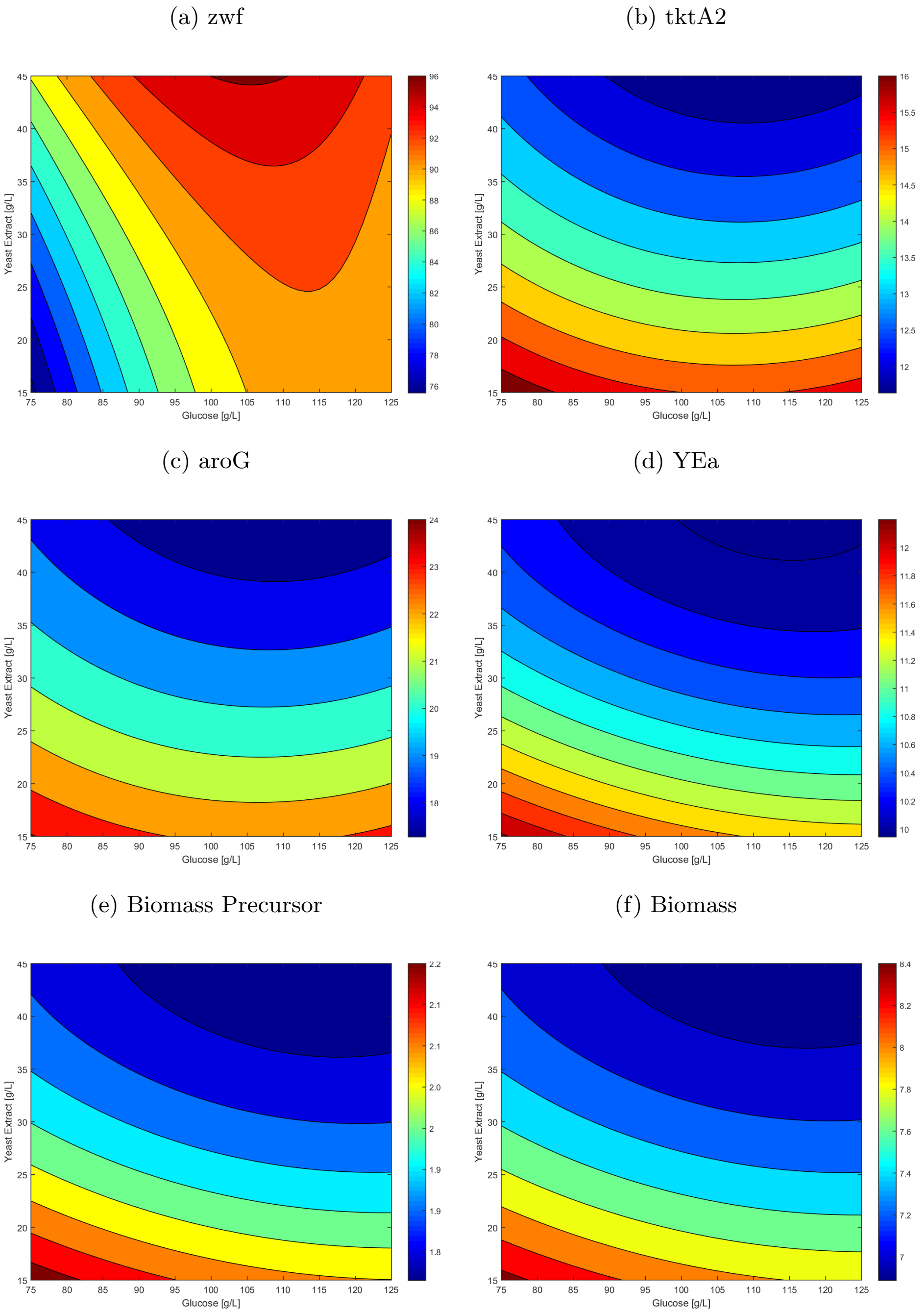


Figure 9: Syntetic operon genes calculated flux IEContours

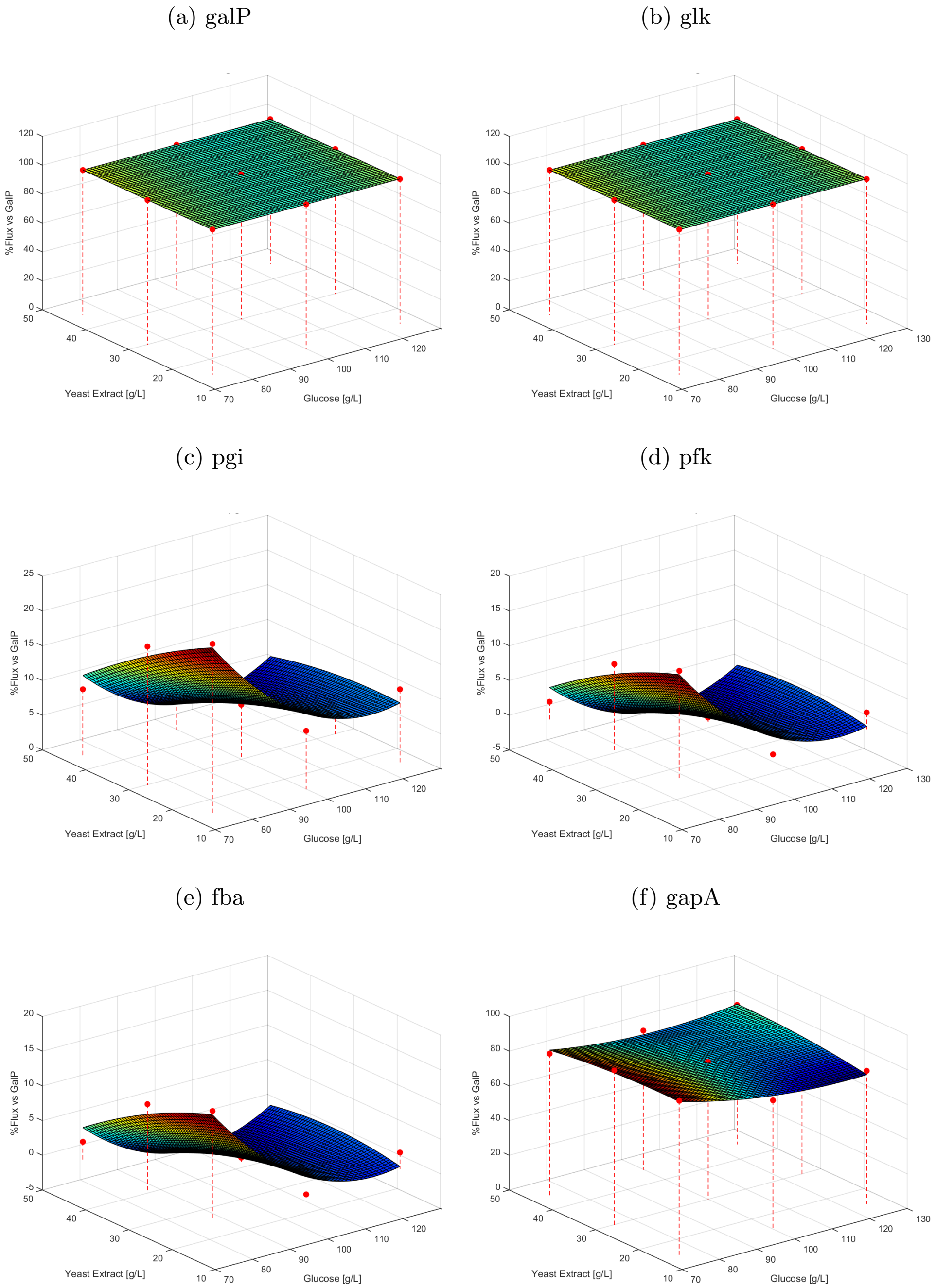


Figure 10: EMP pathway calculated flux IESurfaces pt.1

(a) pgk

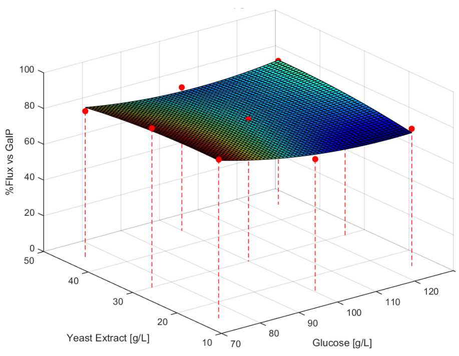


(b) pgm

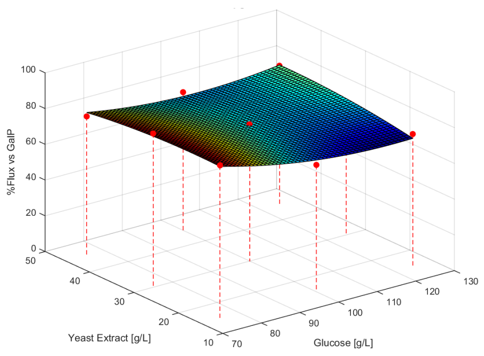


(c) eno

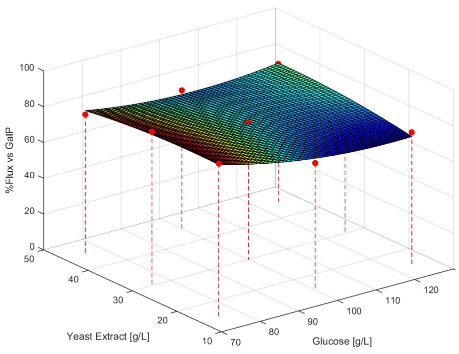


(d) pykA

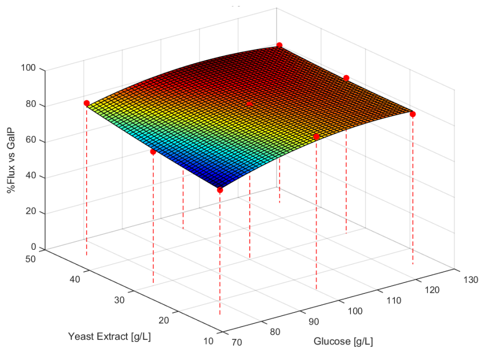


(e) ppsA

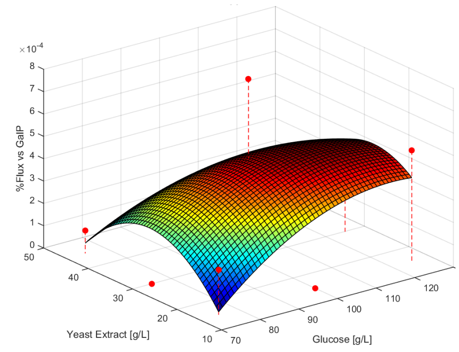


(f) lpdA

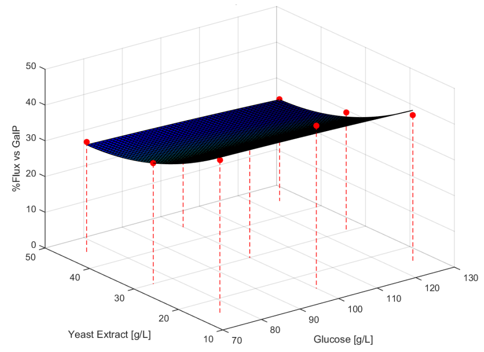


Figure 11: EMP pathway calculated flux IESurfaces pt.2

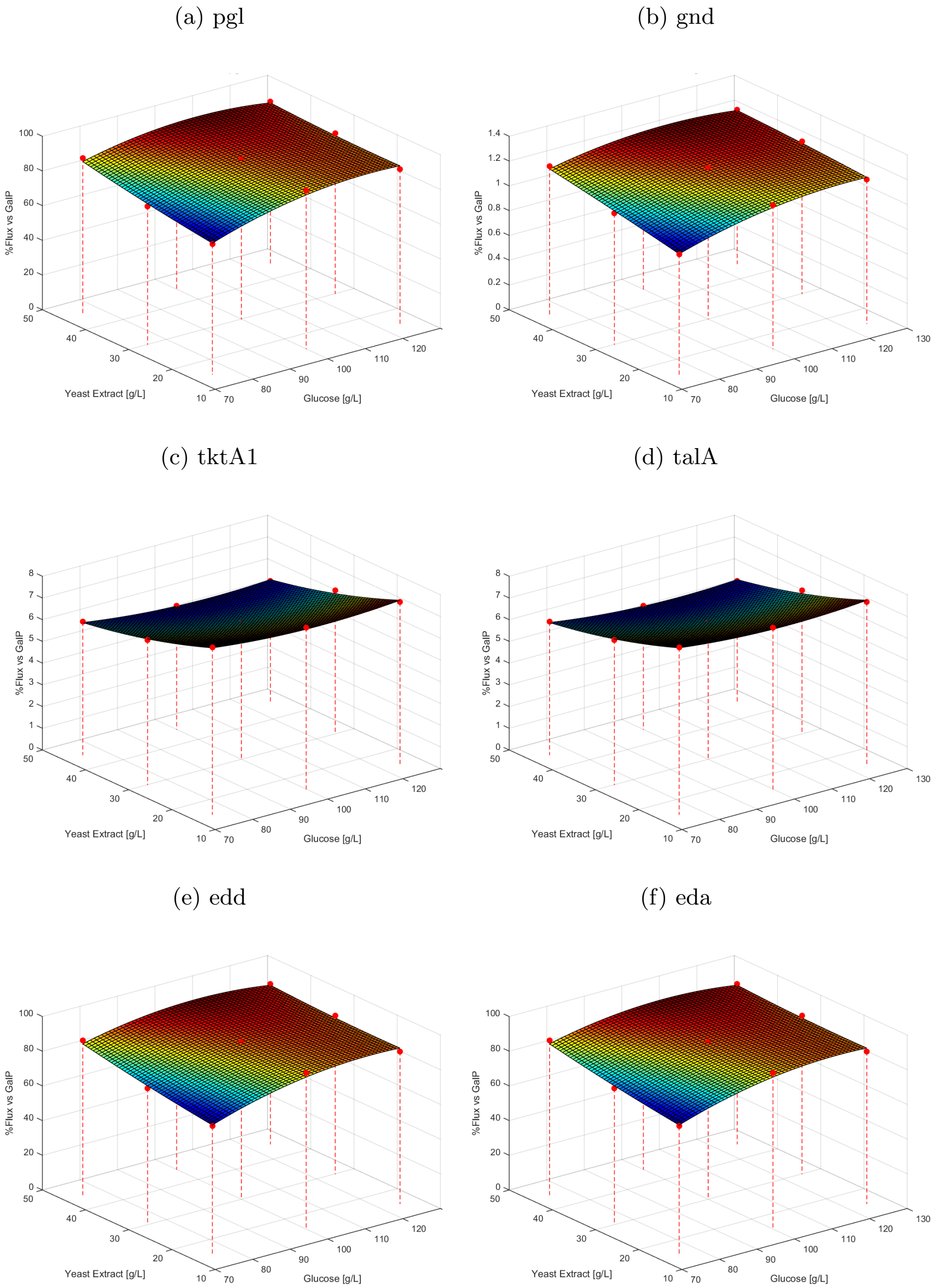


Figure 12: PPP pathway calculated flux IESurfaces

(a) gltA

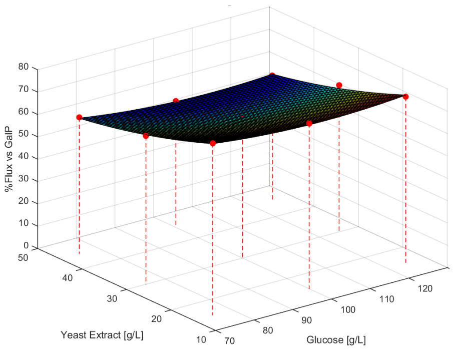


(b) acn

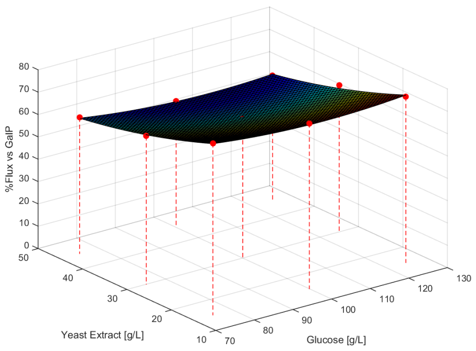


(c) icdA

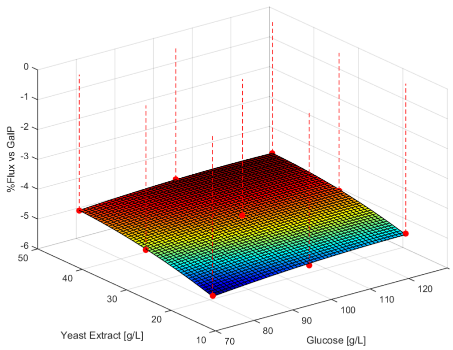


(d) sucAB

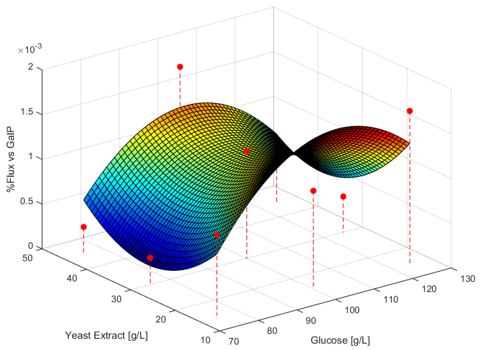


(e) sucCD

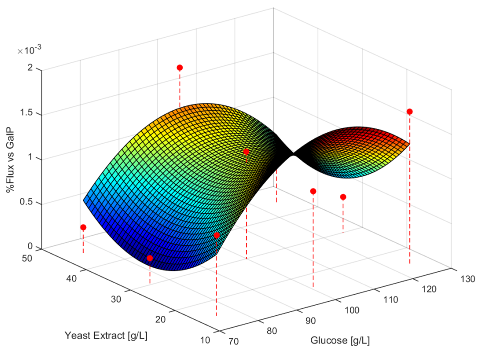


Figure 13: TCA pathway calculated flux IESurfaces pt.1

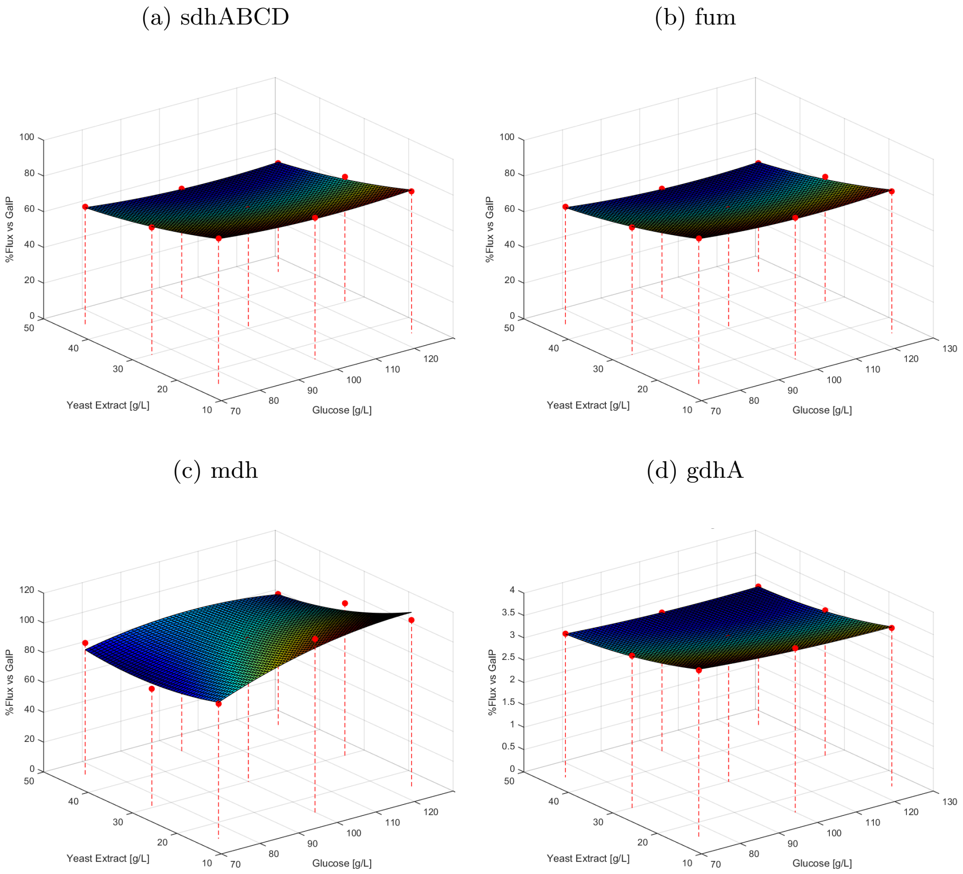


Figure 14: TCA pathway calculated flux IESurfaces pt.2

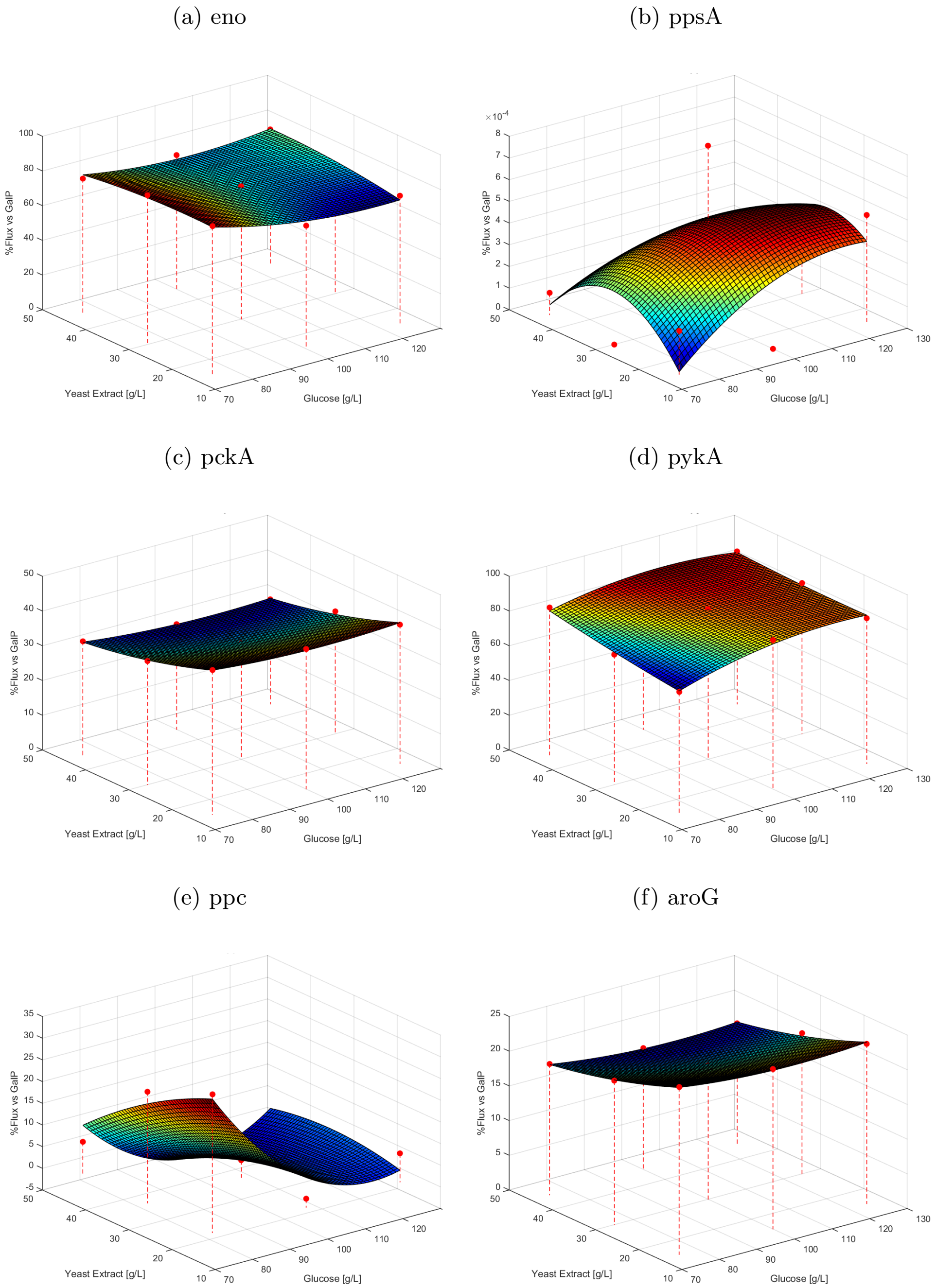


Figure 15: PEP metabolism calculated flux IESurfaces

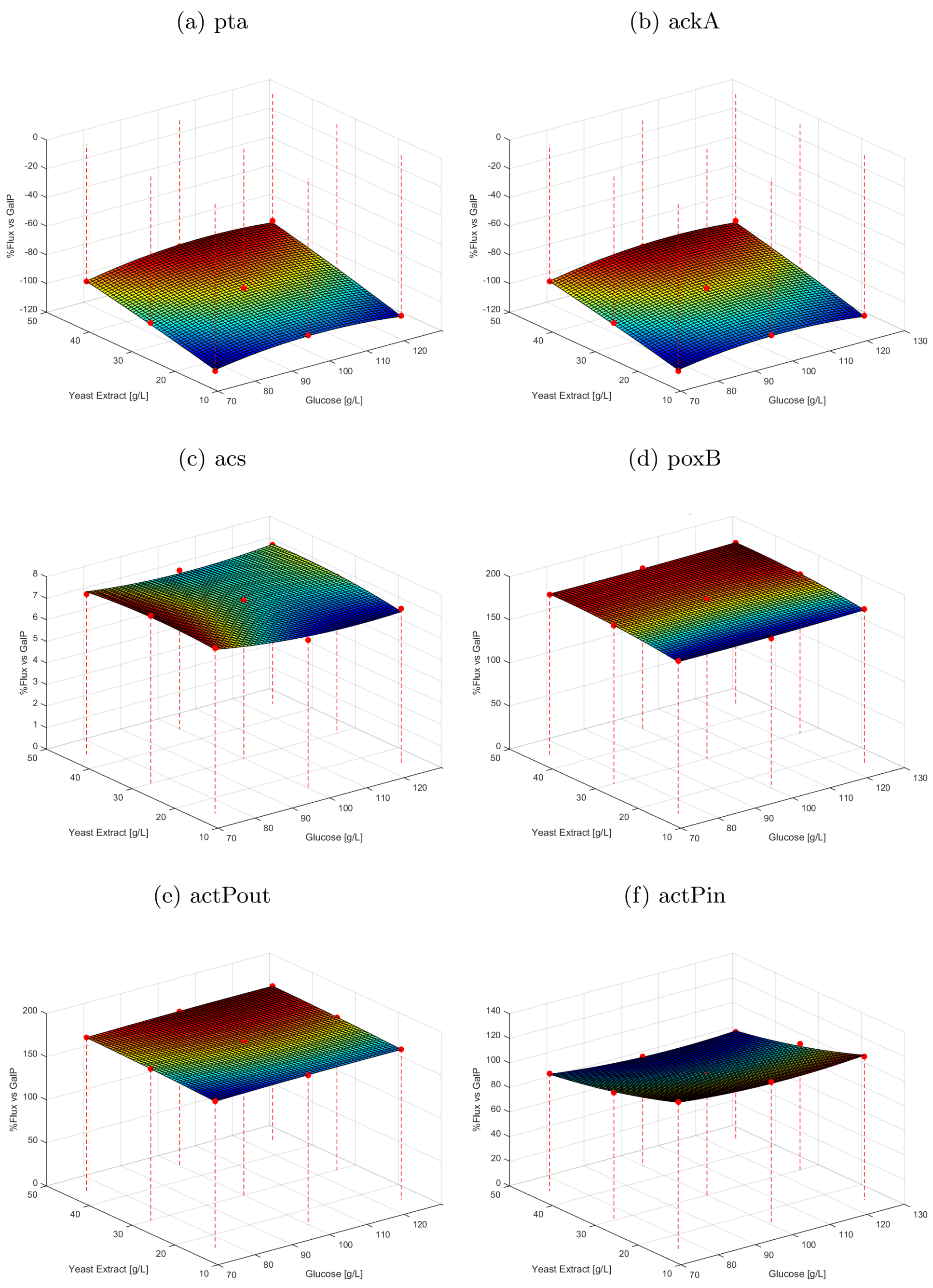


Figure 16: PYR metabolism calculated flux IESurfaces

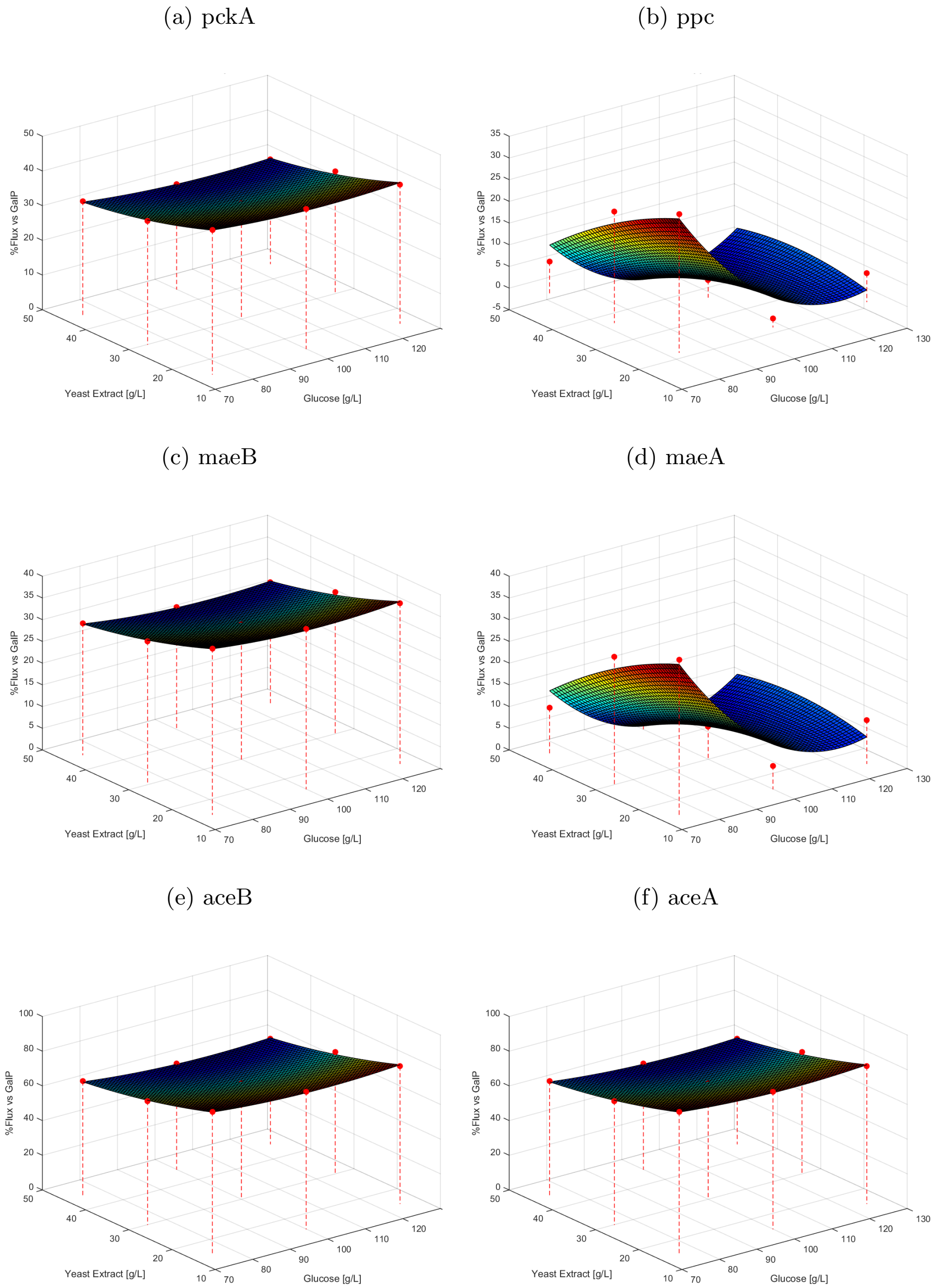


Figure 17: Anaplerotic genes calculated flux IESurfaces

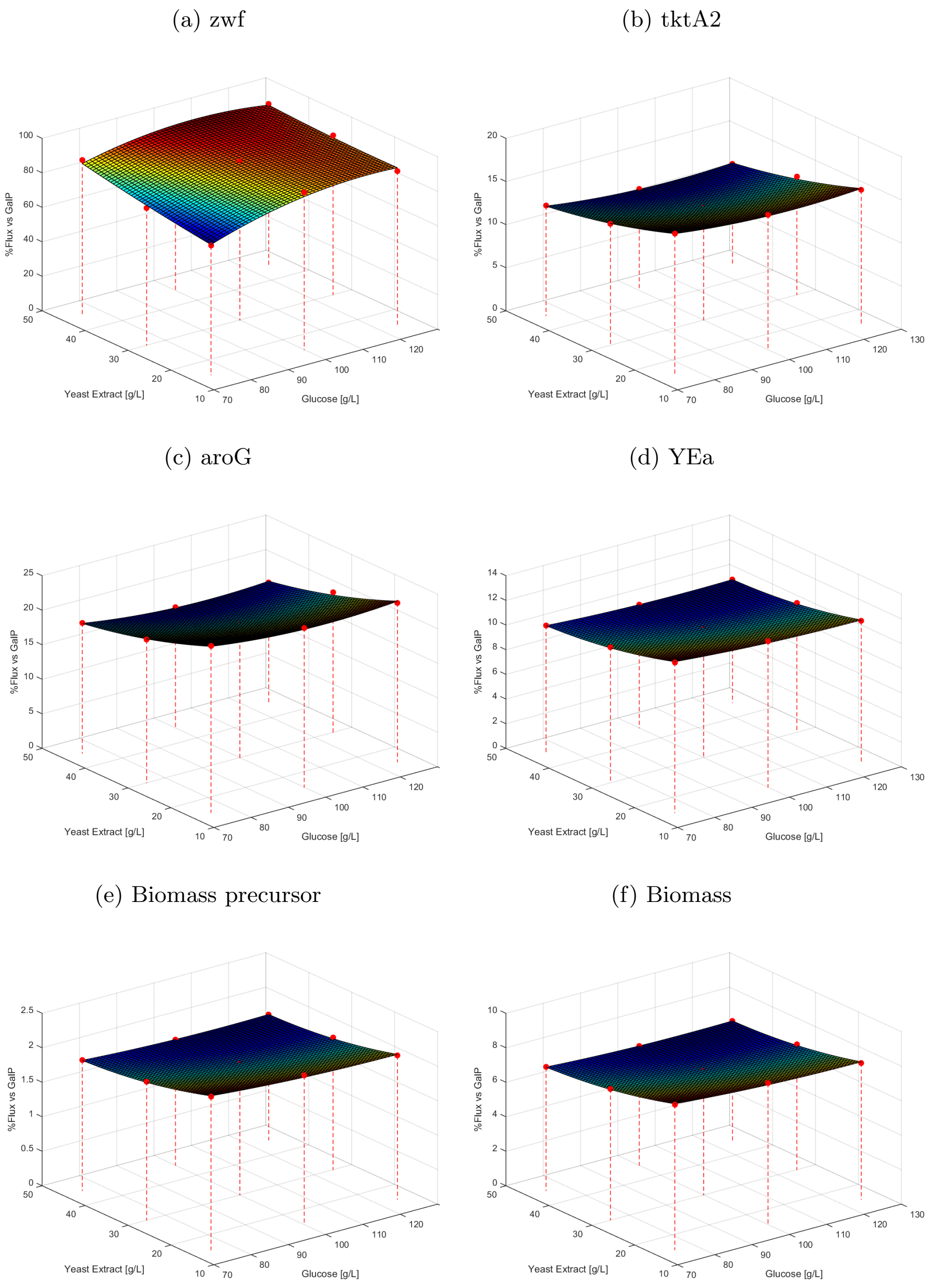


Figure 18: Syntetic operon genes calculated flux IESurfaces

# Mid Exponential Flux Response Surfaces

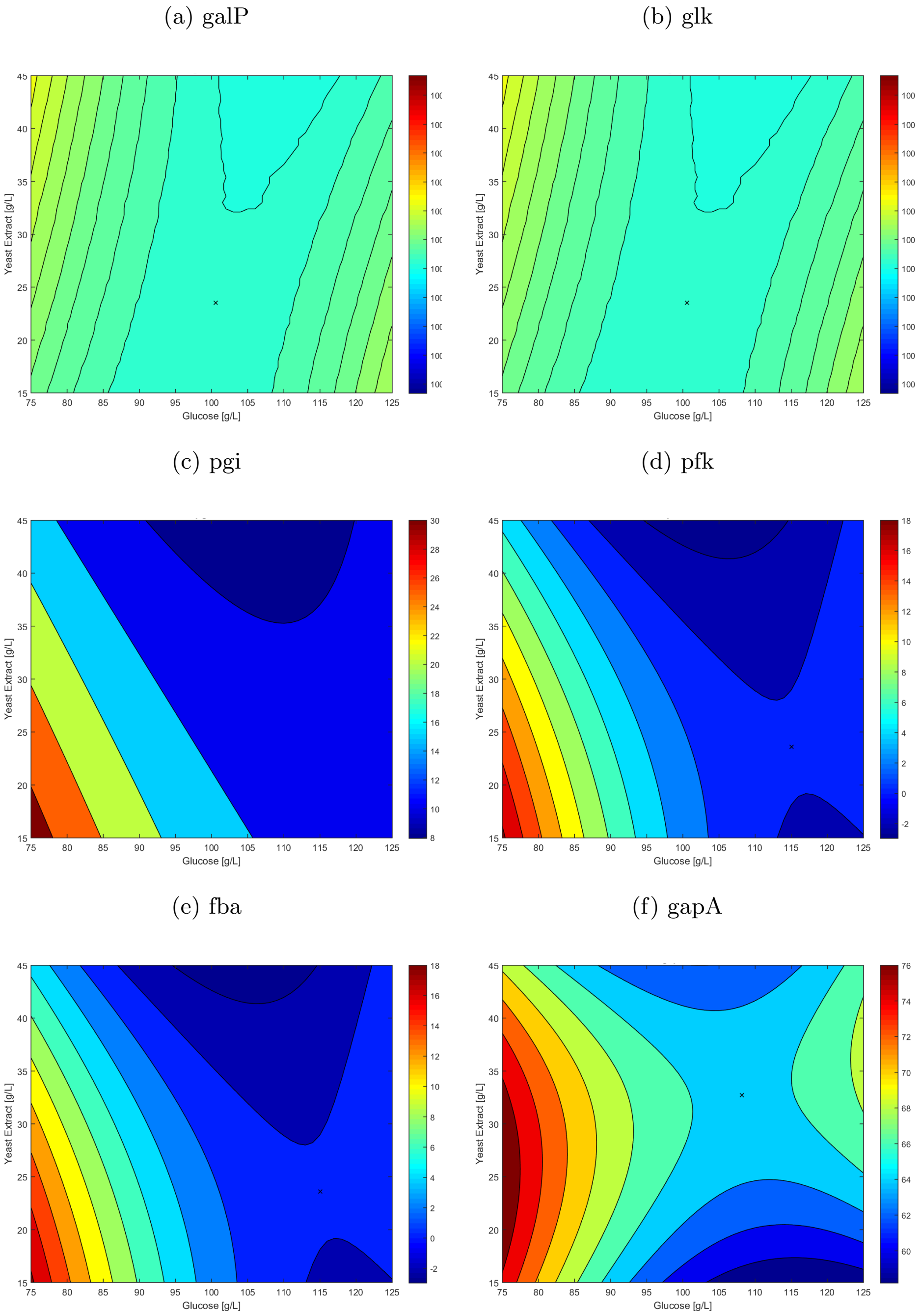


Figure 19: EMP pathway calculated flux MEContours pt.1

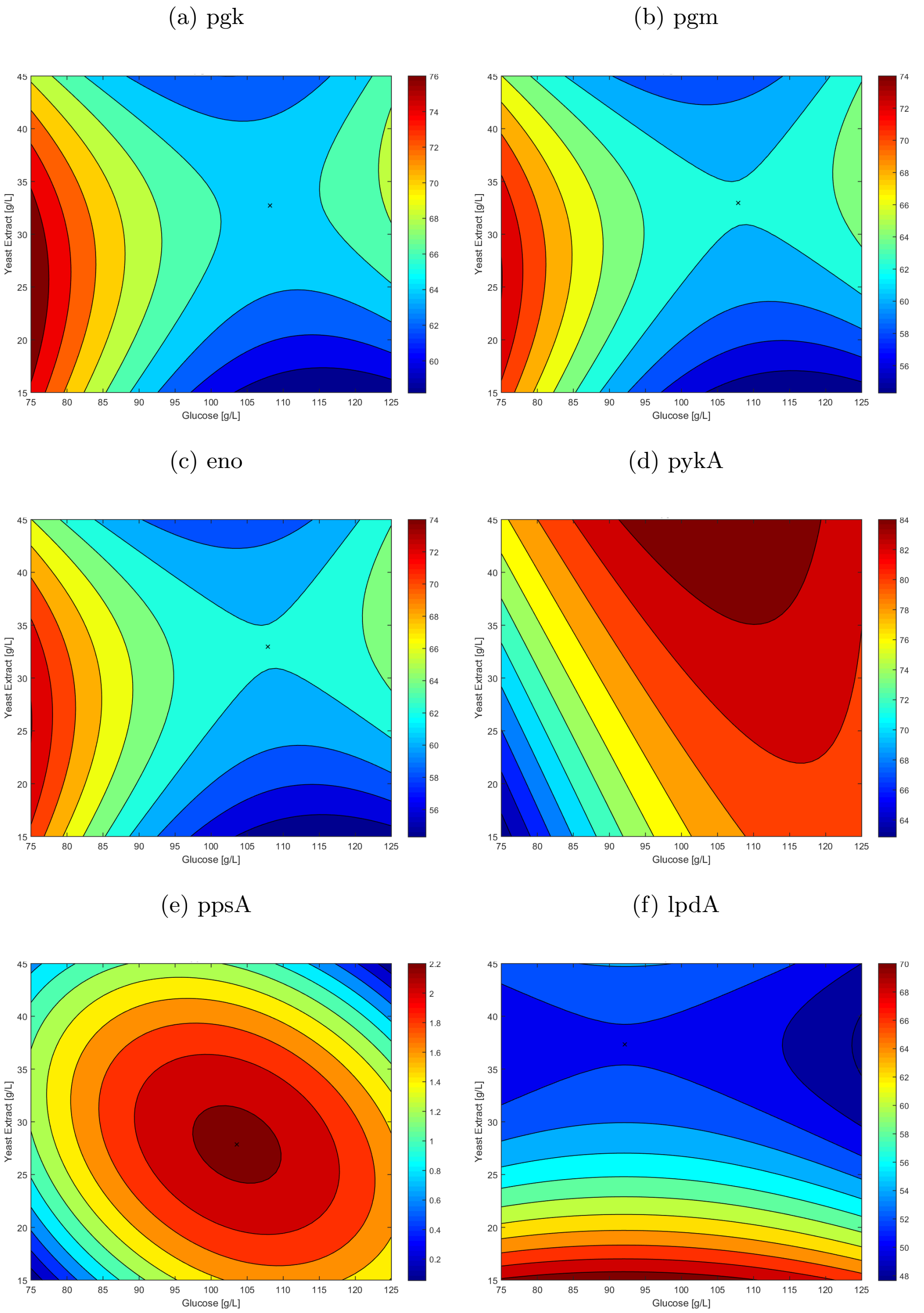


Figure 20: EMP pathway calculated flux MEContours pt.2

(a) pgl

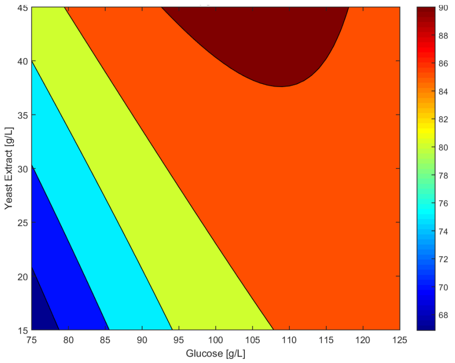


(b) gnd

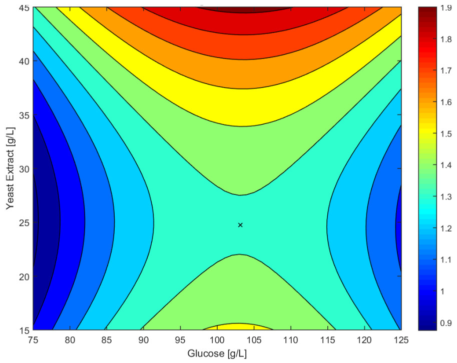


(c) tktA1

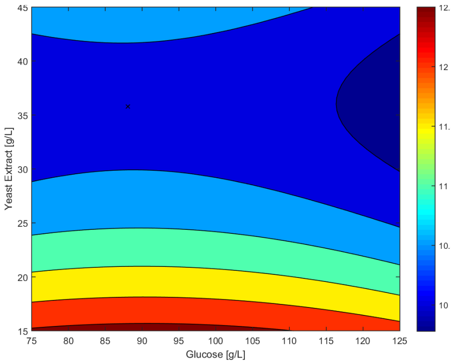


(d) talA

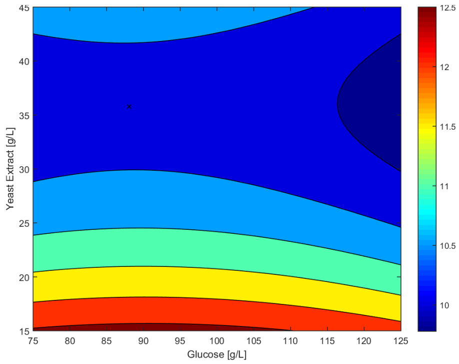


(e) edd

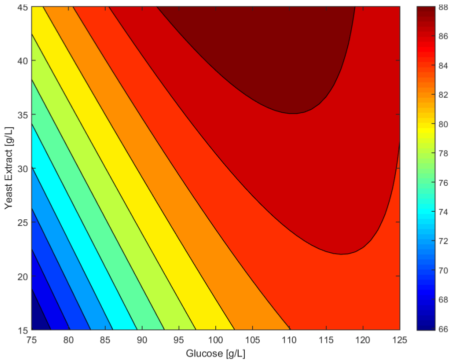


(f) eda

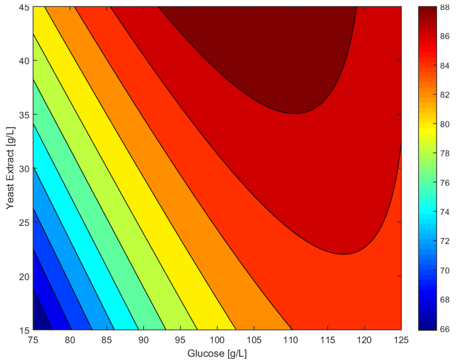


Figure 21: PPP pathway calculated flux MEContours

(a) gltA

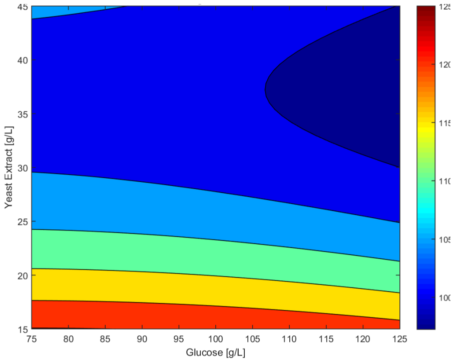


(b) acn

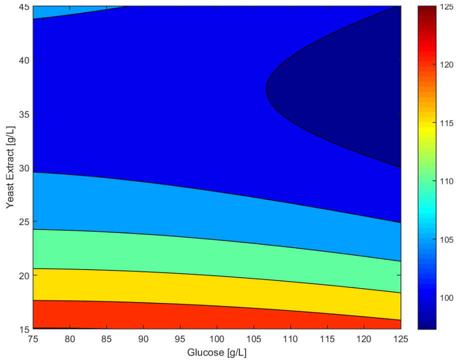


(c) icdA

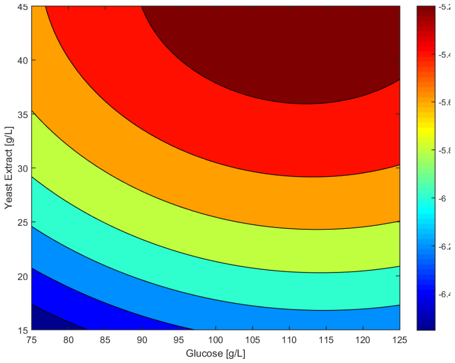


(d) sucAB

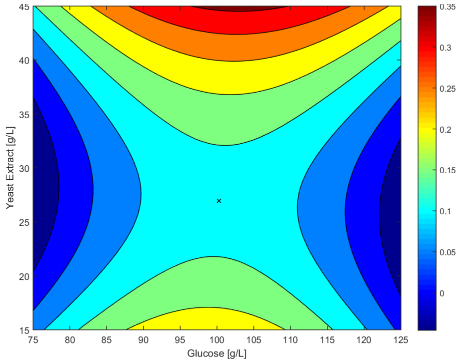


(e) sucCD

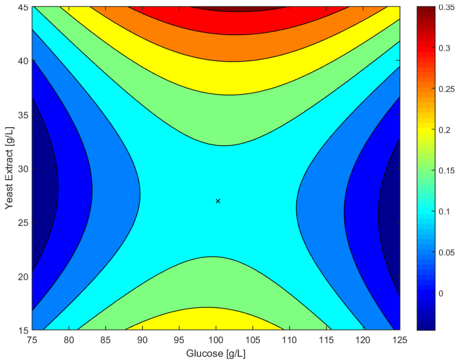


Figure 22: TCA pathway calculated flux MEContours pt.1

(a) sdhABCD

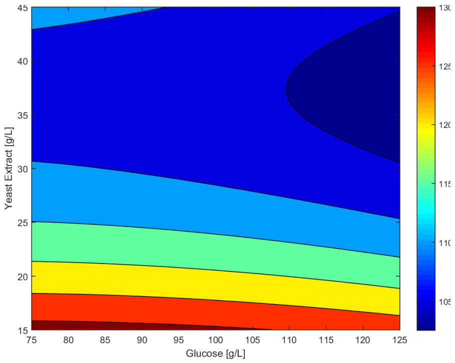


(b) fum

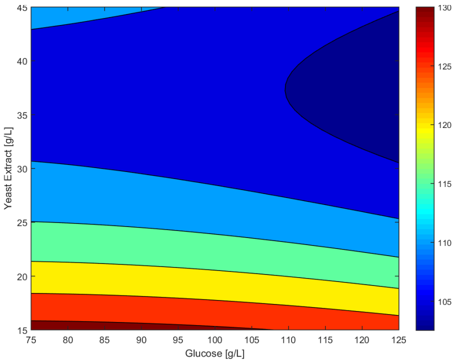


(c) mdh

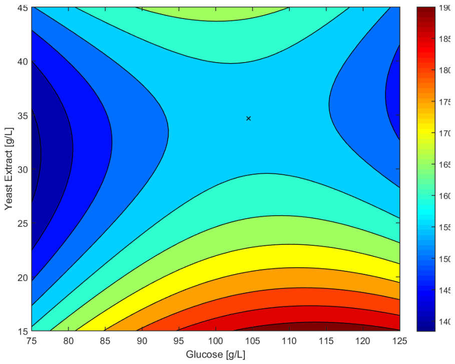


(d) gdhA

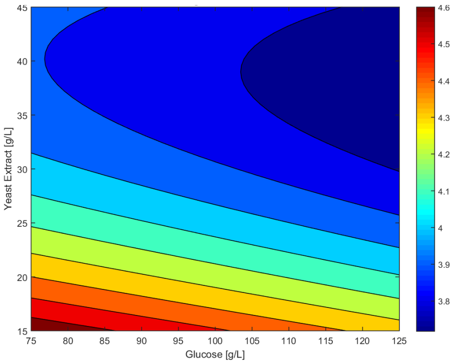


Figure 23: TCA pathway calculated flux MEContours pt.2

(a) eno

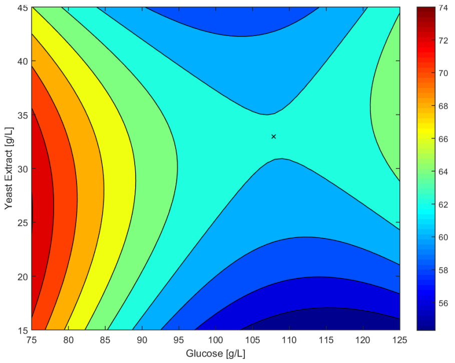


(b) ppsA

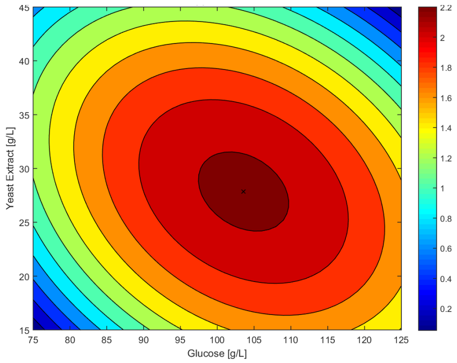


(c) pckA

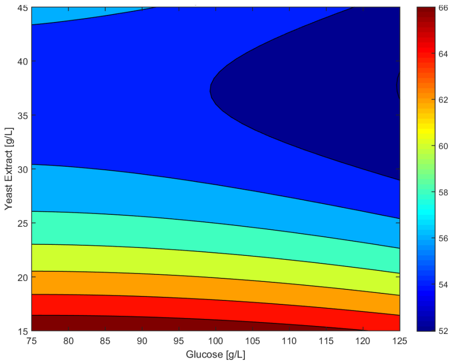


(d) pykA

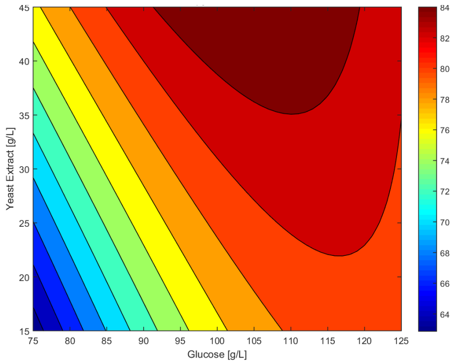


(e) ppc

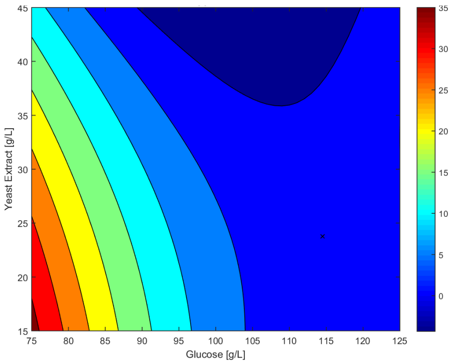


(f) aroG

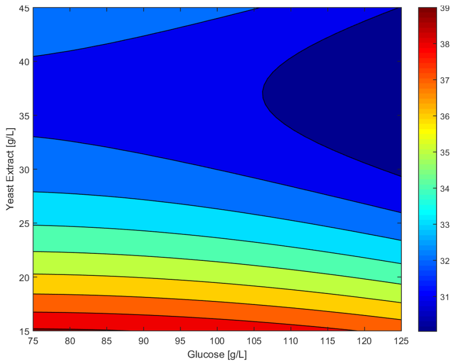


Figure 24: PEP metabolism calculated flux MEContours

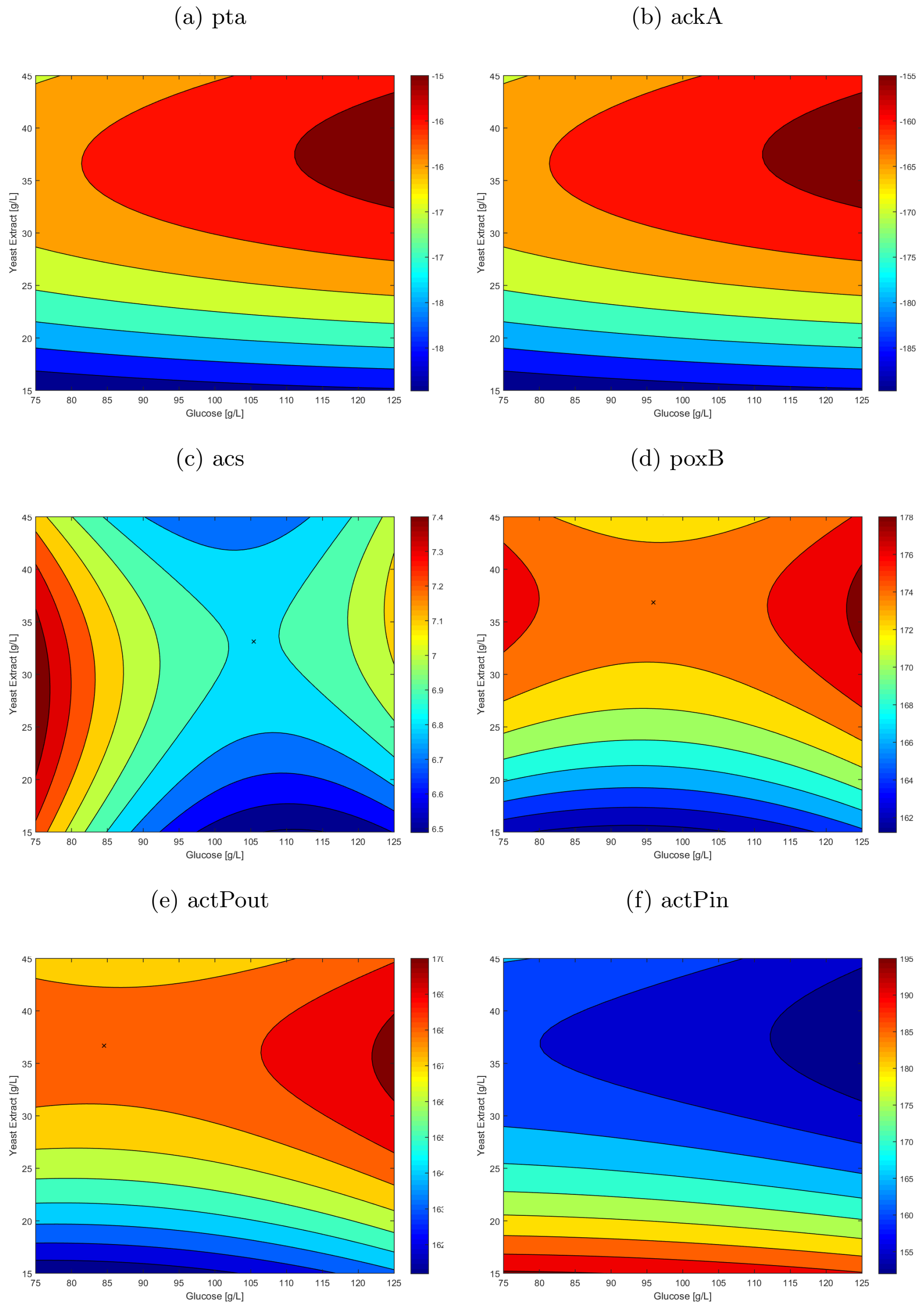


Figure 25: PYR metabolism calculated flux MEContours

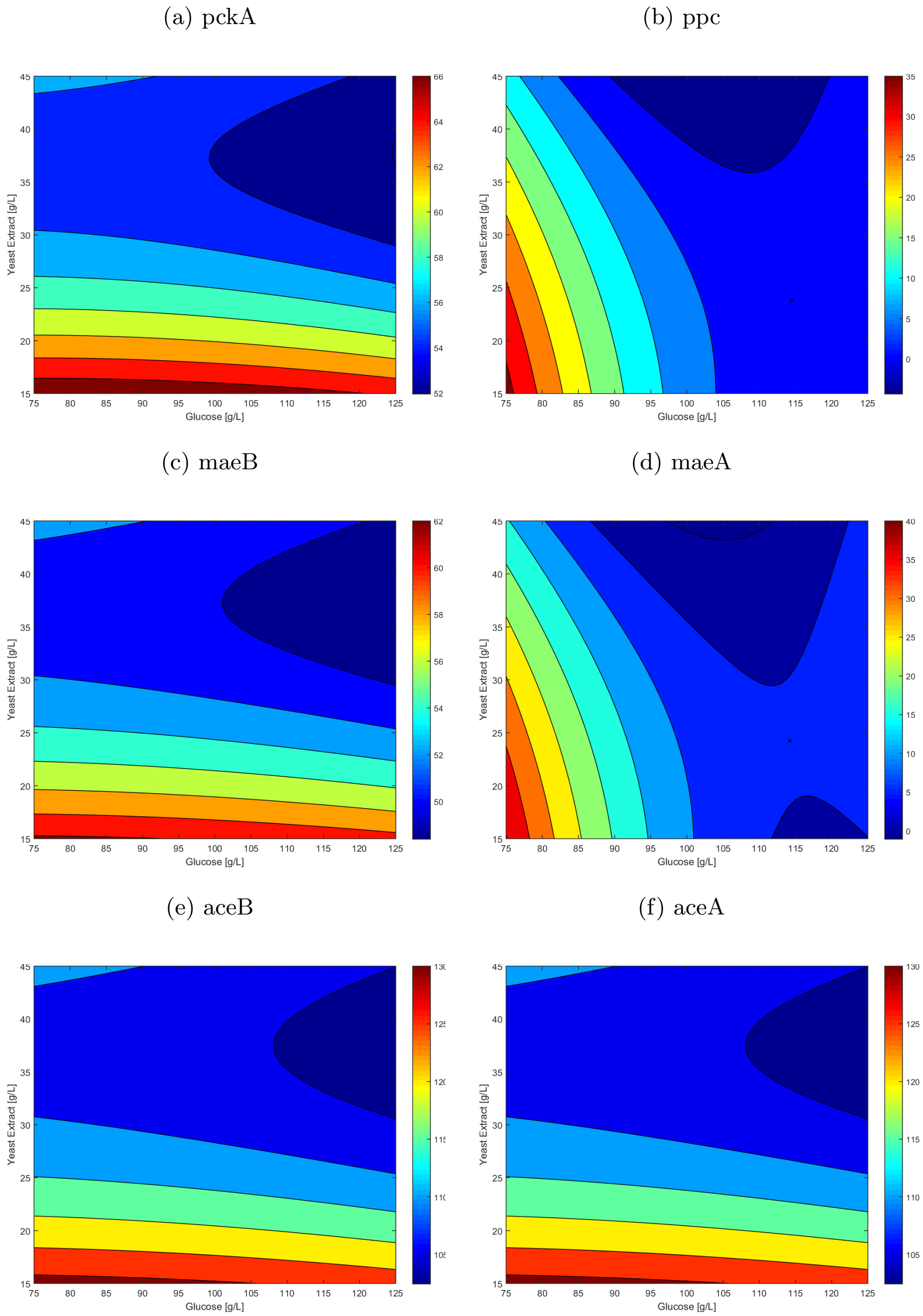


Figure 26: Anaplerotic genes calculated flux MEContours

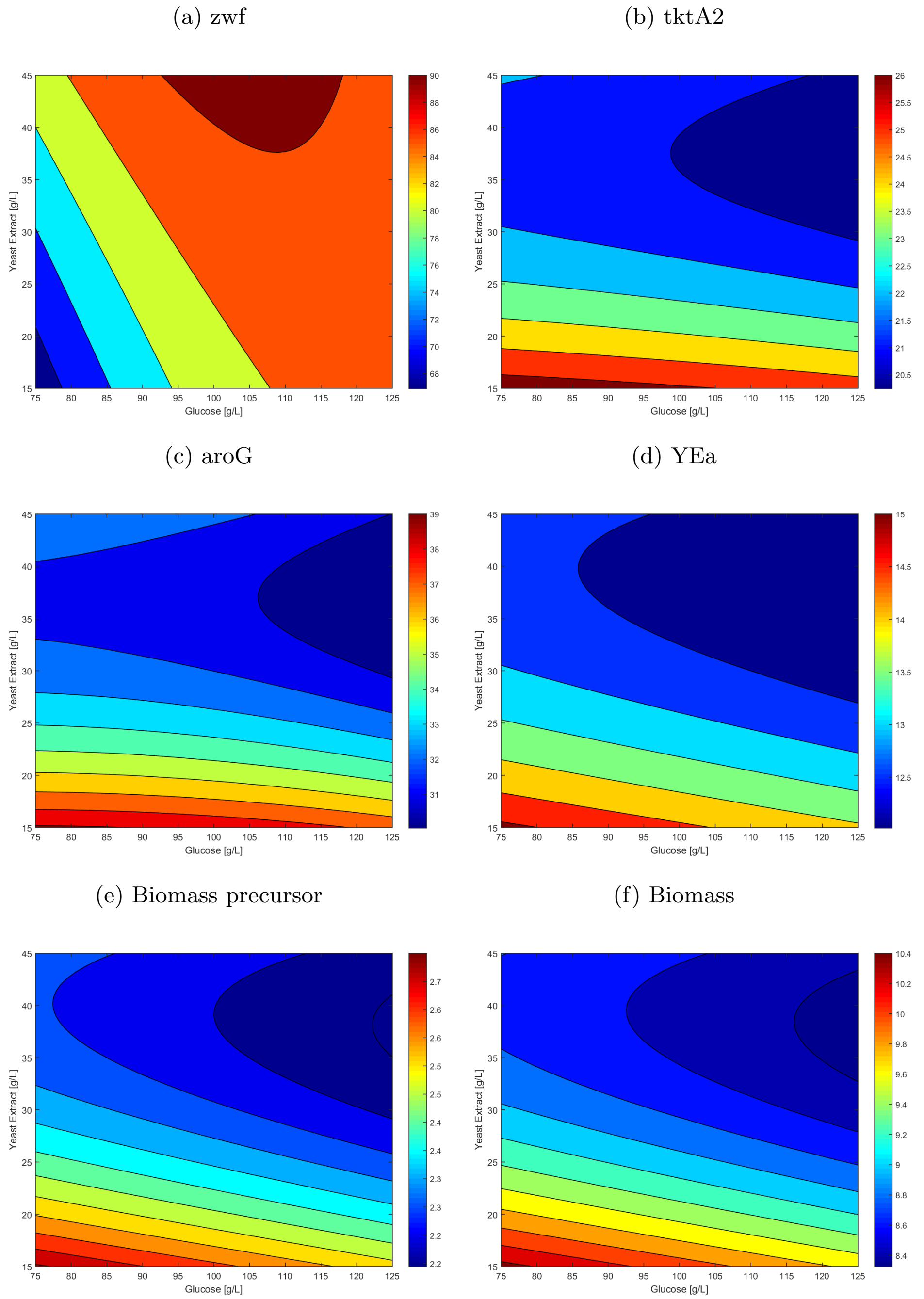


Figure 27: Syntetic operon genes calculated flux MEContours

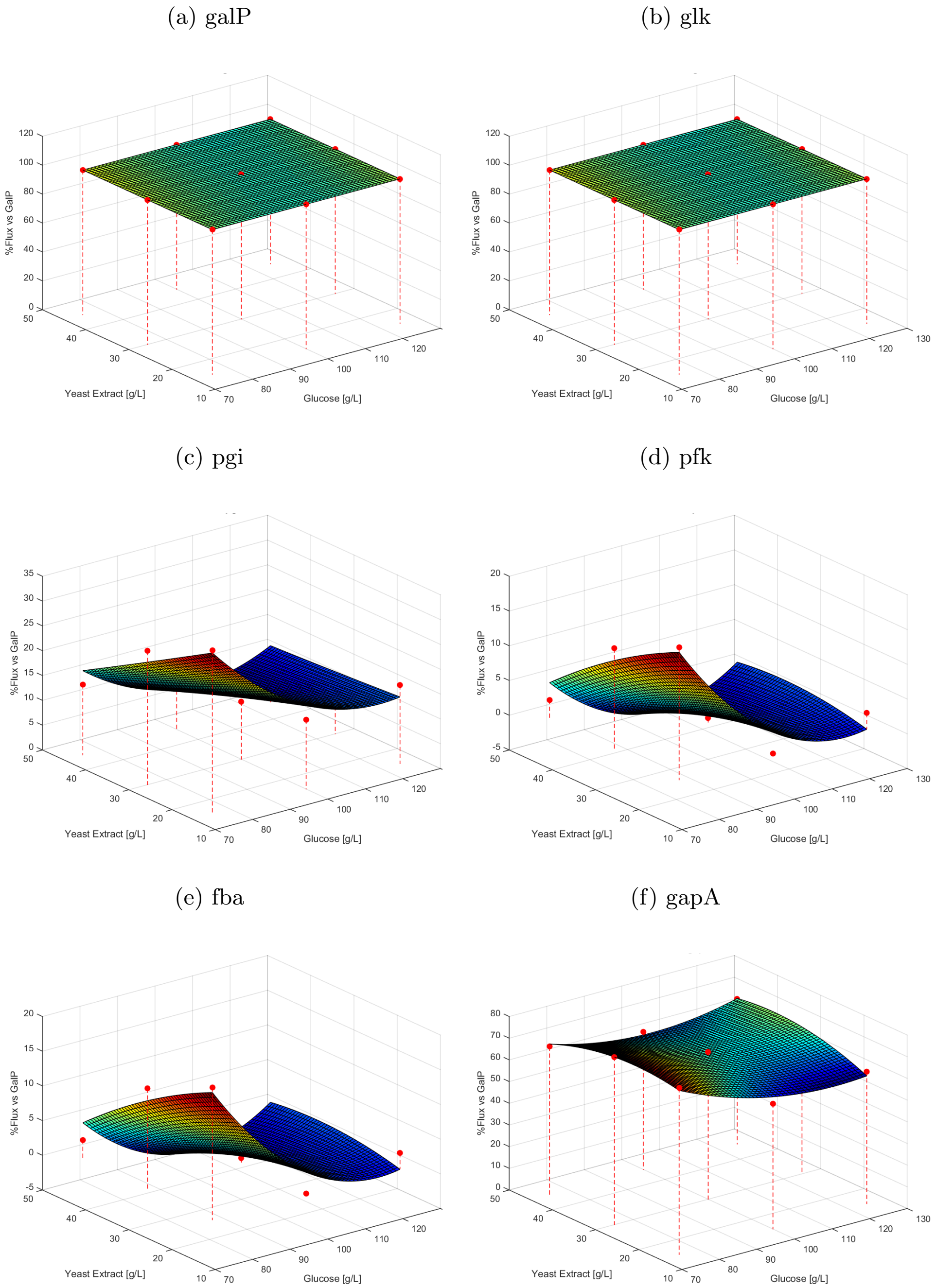


Figure 28: EMP pathway calculated flux MESurfaces pt.1

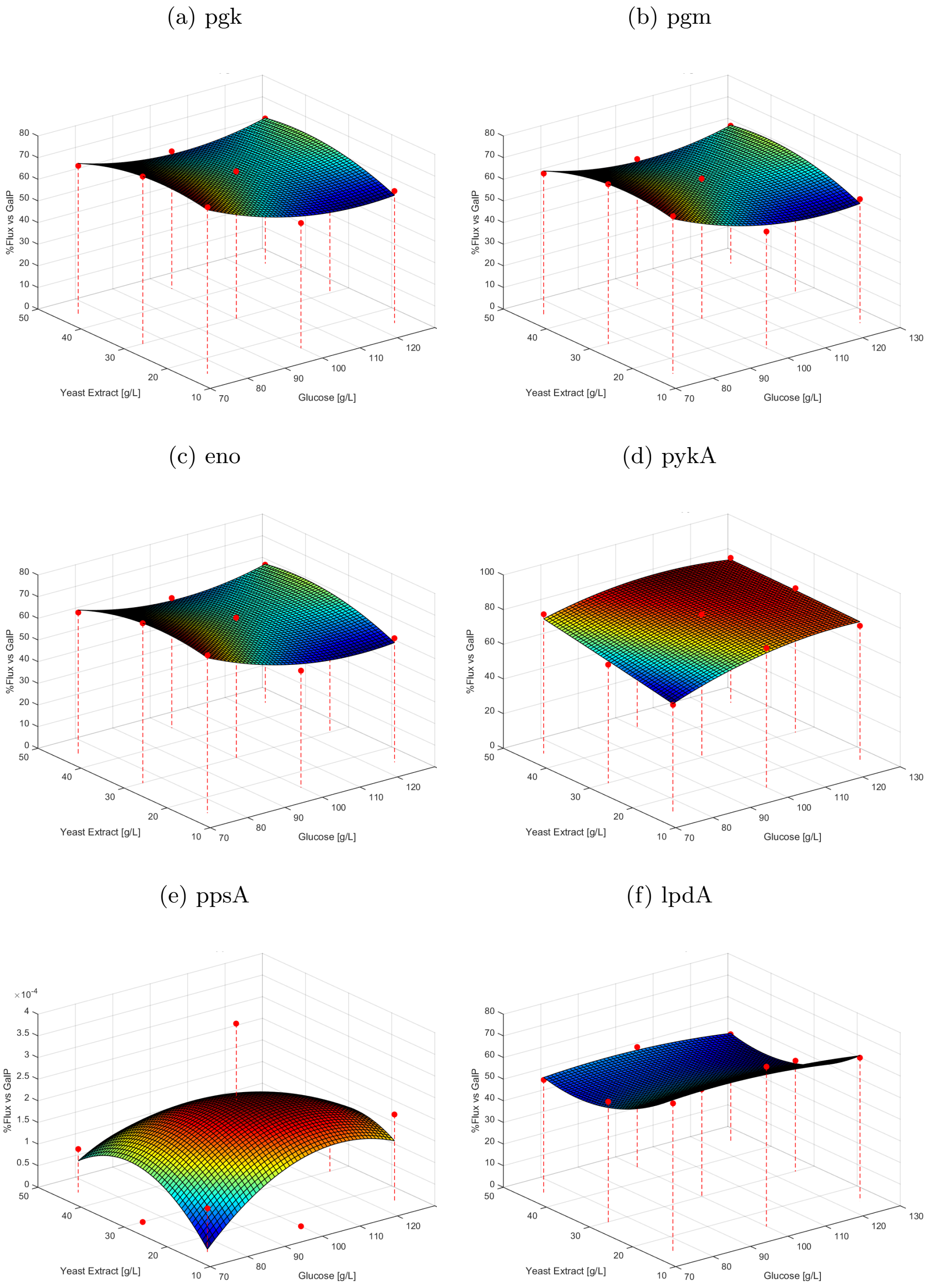


Figure 29: EMP pathway calculated flux MESurfaces pt.2

(a) pgl

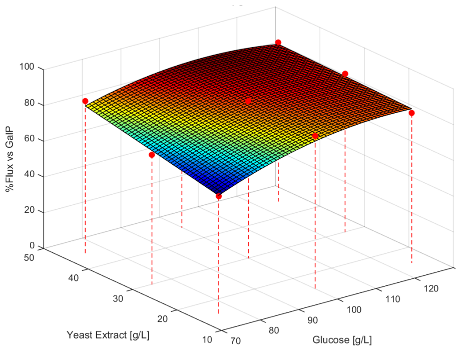


(b) gnd

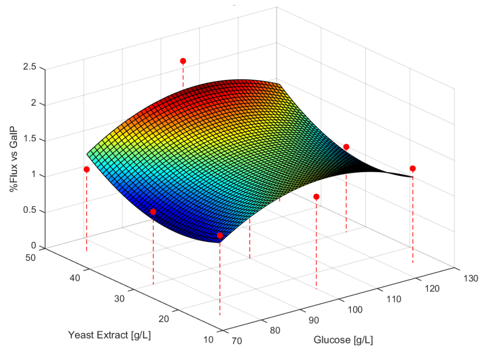


(c) tktA1

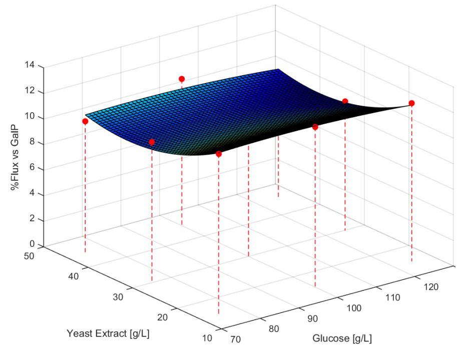


(d) talA

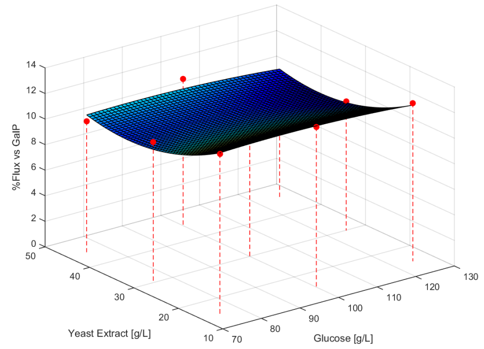


(e) edd

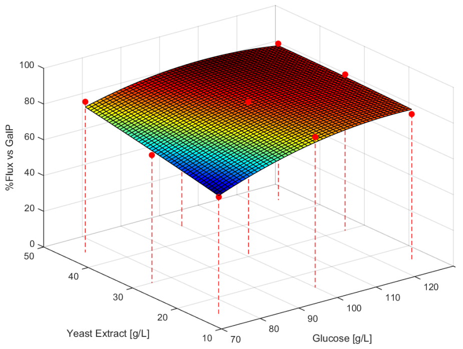


(f) eda

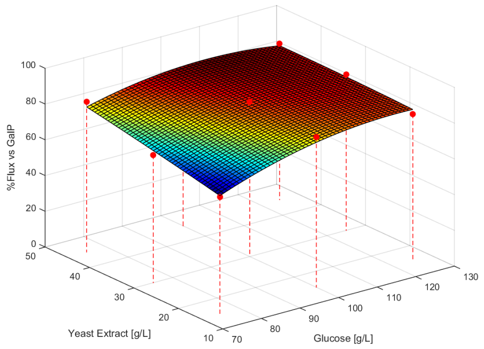


Figure 30: PPP pathway calculated flux MESurfaces

(a) gltA

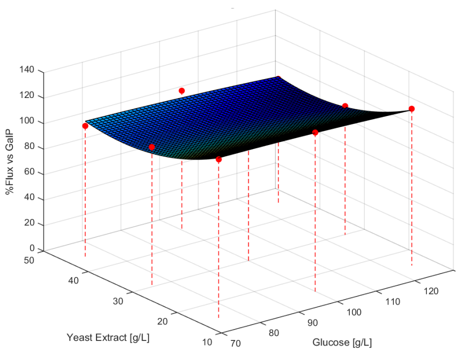


(b) acn

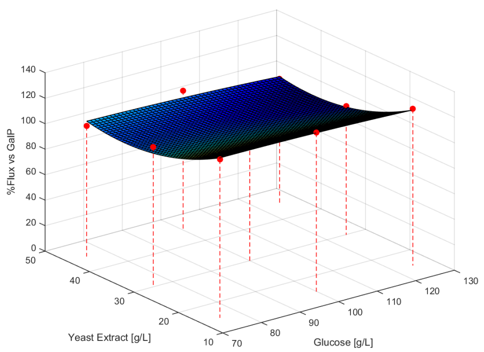


(c) icdA

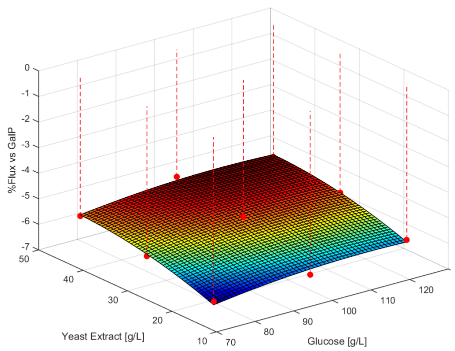


(d) sucAB

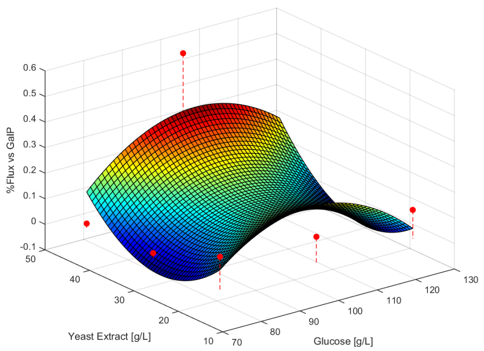


(e) sucCD

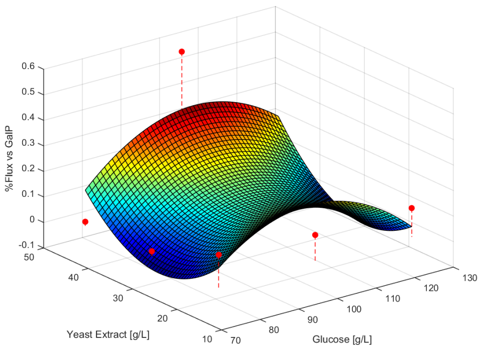


Figure 31: TCA pathway calculated flux MESurfaces pt.1

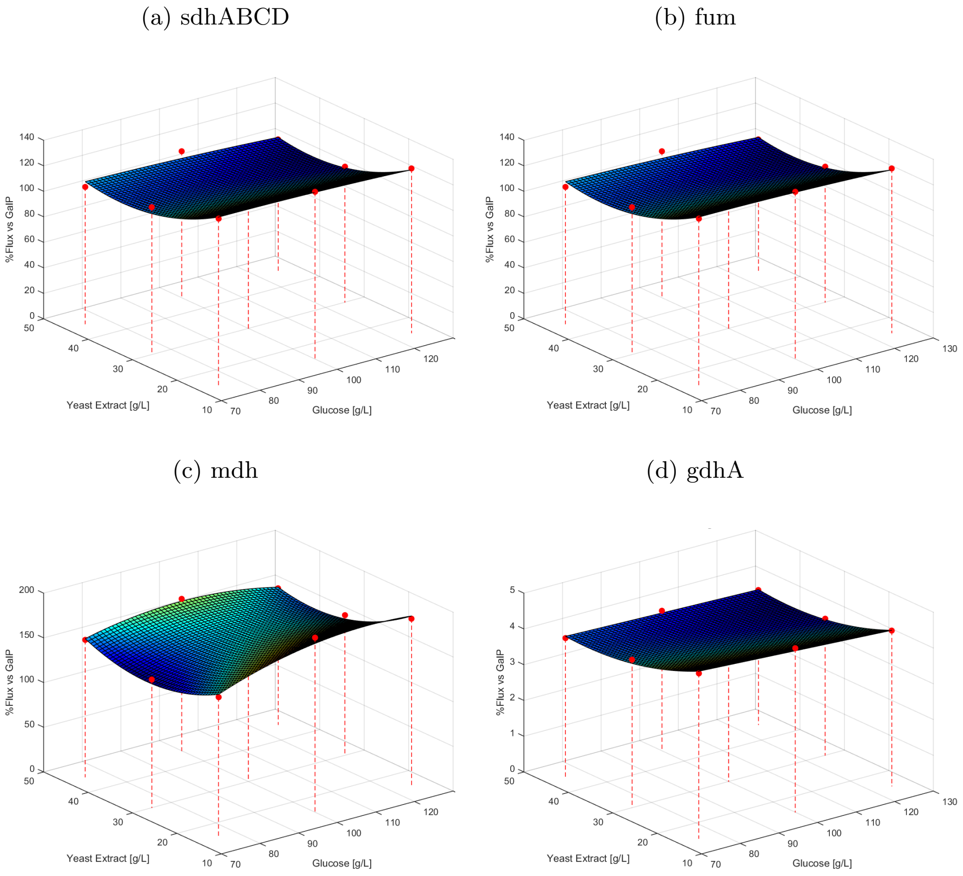


Figure 32: TCA pathway calculated flux MESurfaces pt.2

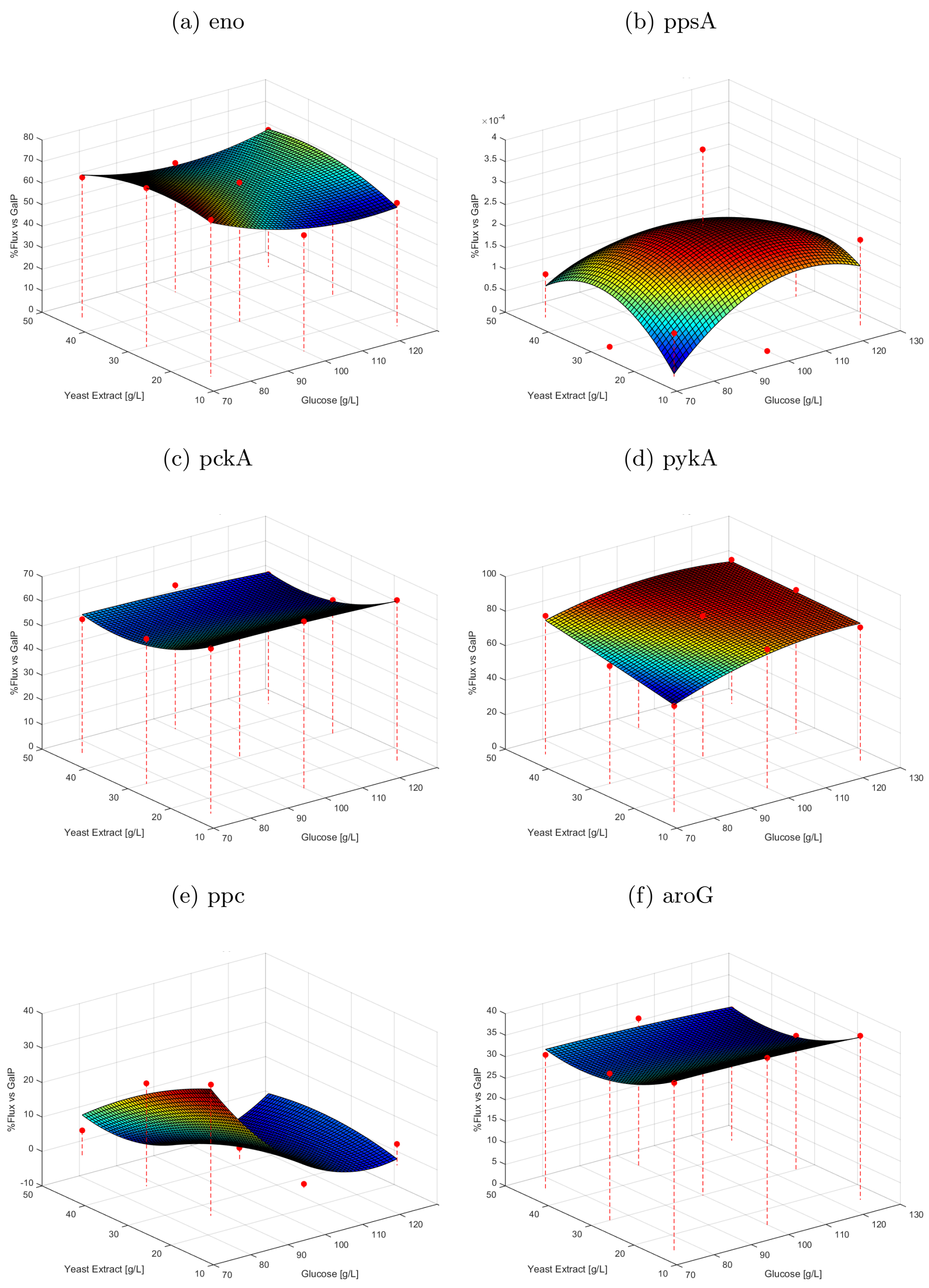


Figure 33: PEP metabolism calculated flux MESurfaces

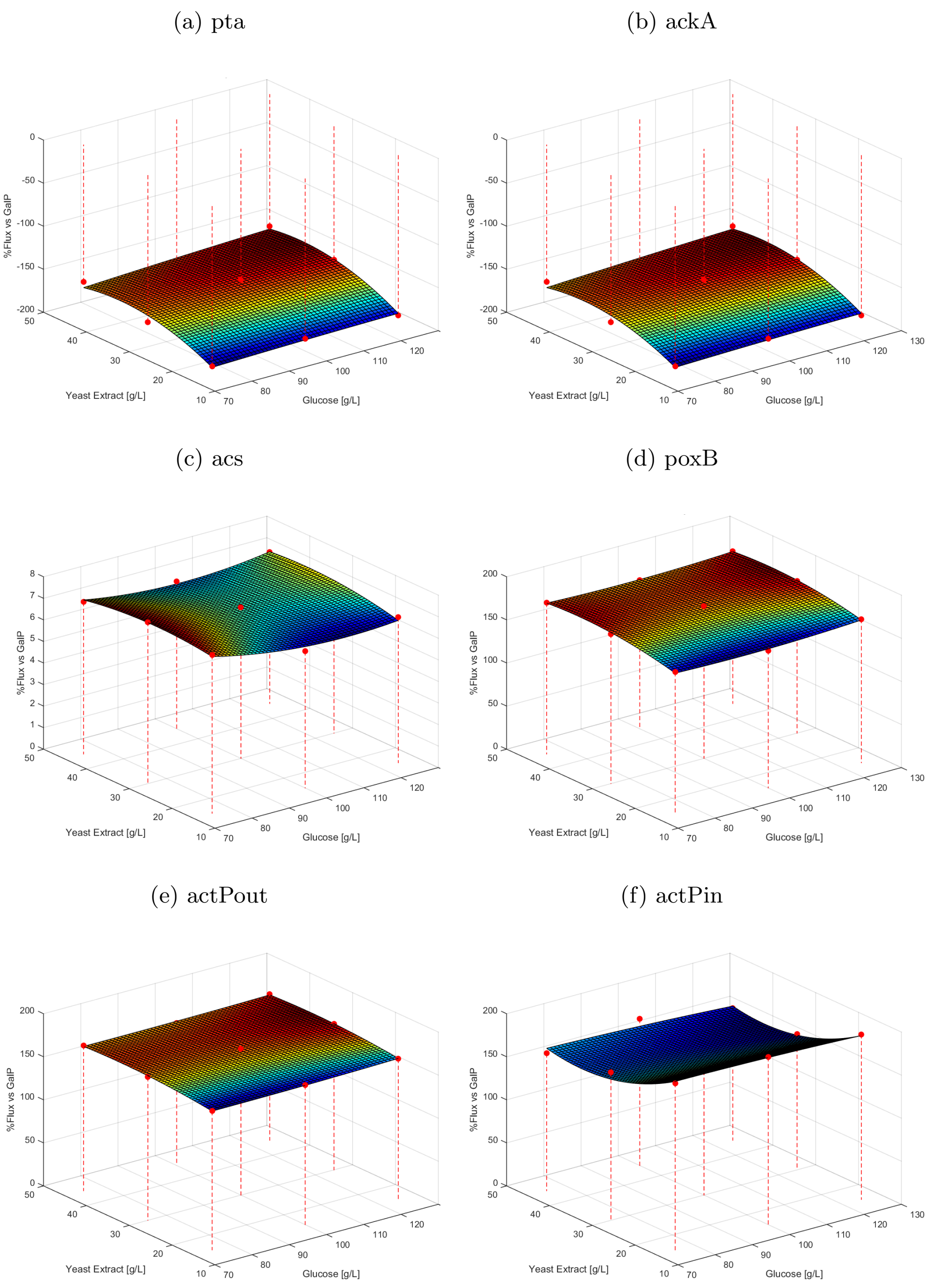


Figure 34: PYR metabolism calculated flux MESurfaces

(a) pckA

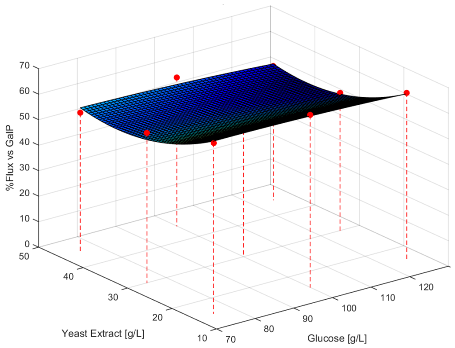


(b) ppc

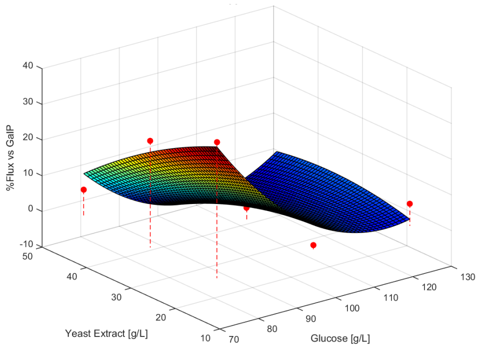


(c) maeB

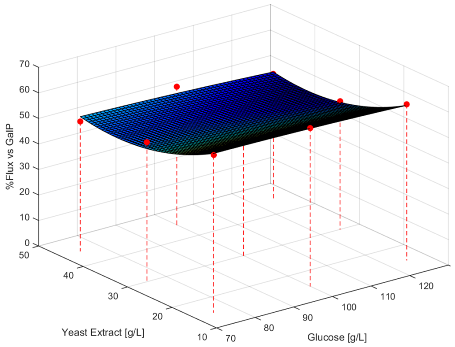


(d) maeA

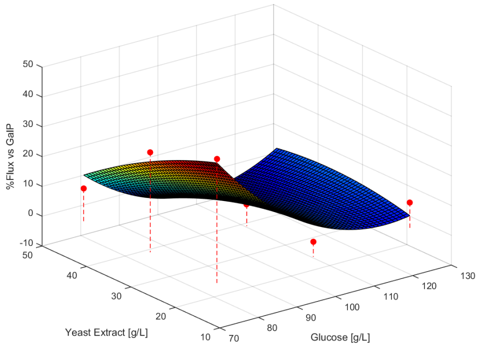


(e) aceB

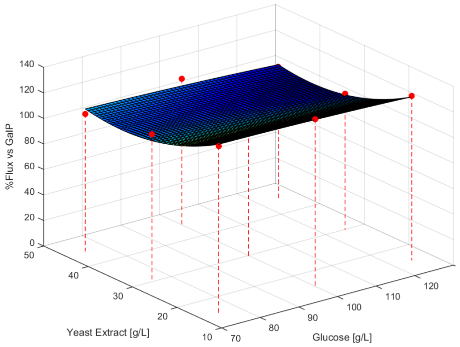


(f) aceA

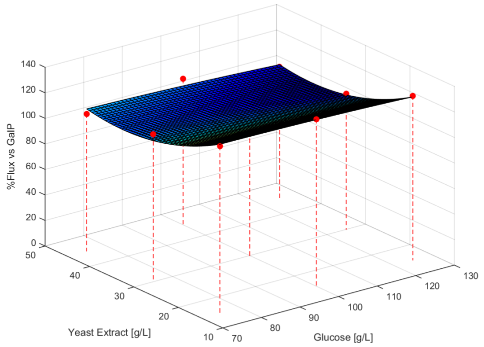


Figure 35: Anaplerotic genes calculated flux MESurfaces

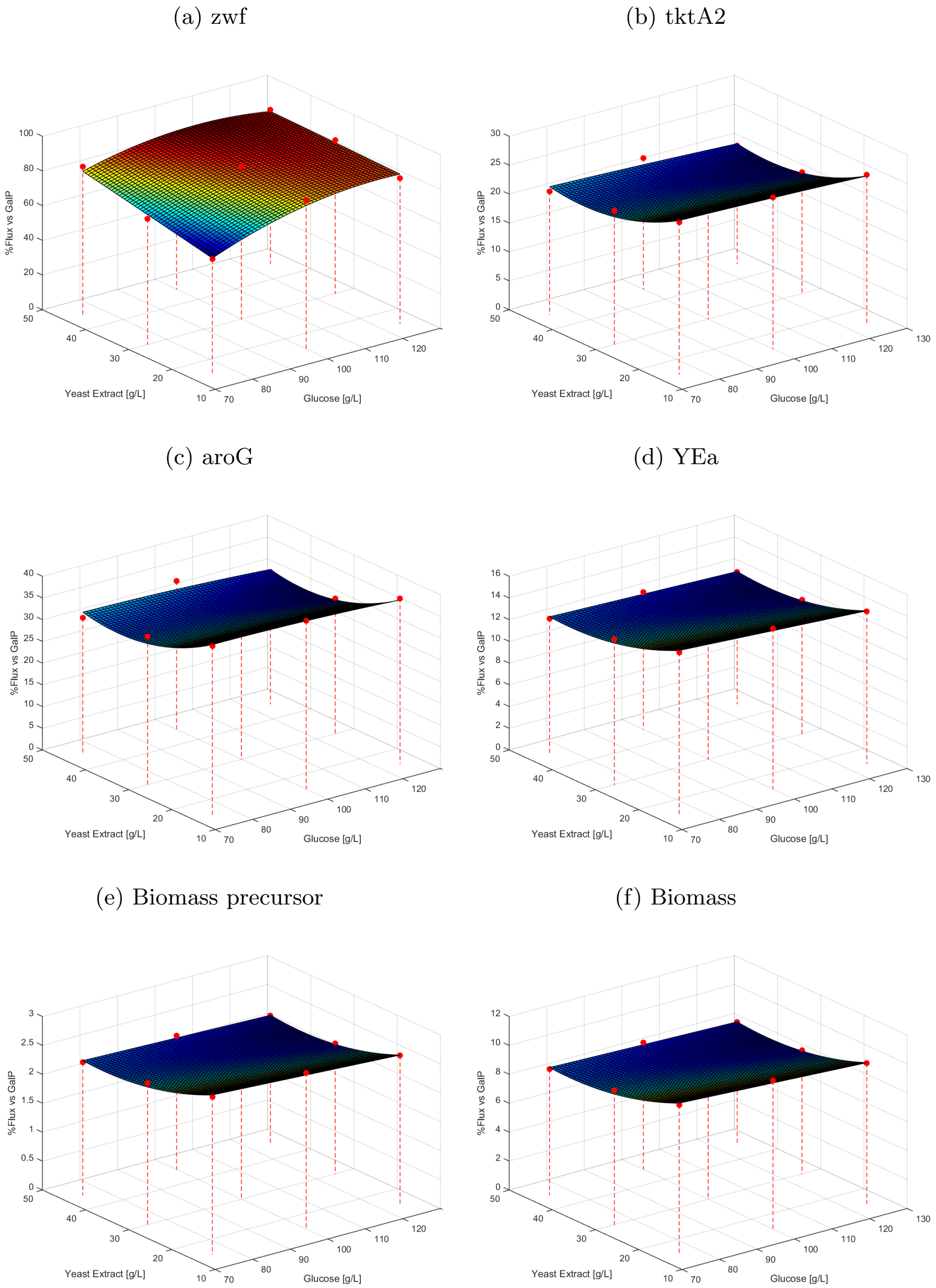


Figure 36: Syntetic operon genes calculated flux MESurfaces

# Mid Stationary Flux Response Surfaces

(a) galP

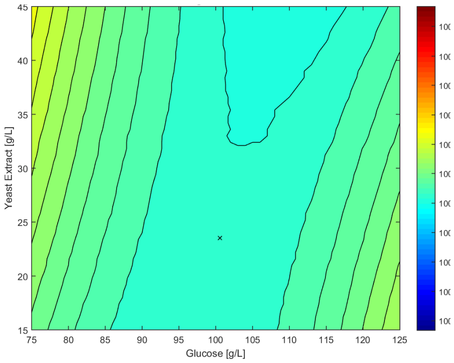


(b) glk

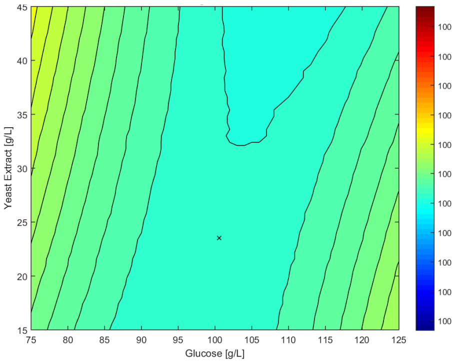


(c) pgi

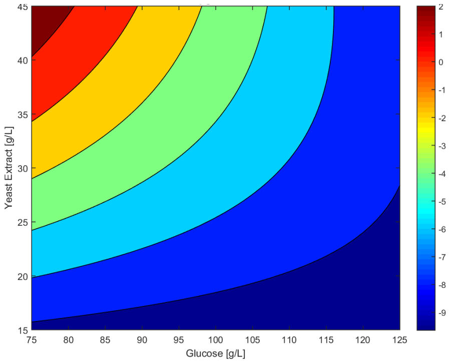


(d) pfk

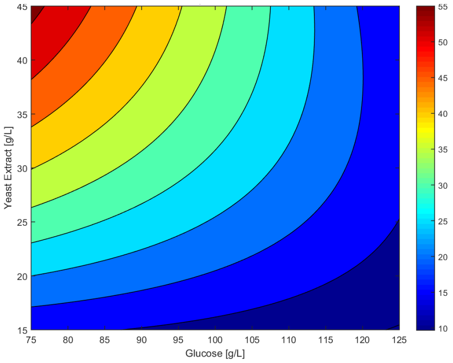


(e) fba

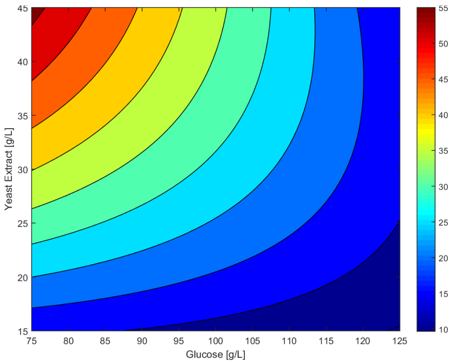


(f) gapA

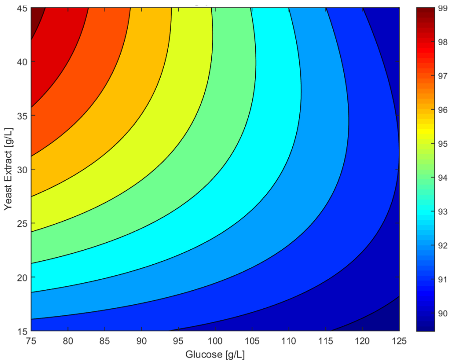


Figure 37: EMP pathway calculated flux MSContours pt.1

(a) pgk

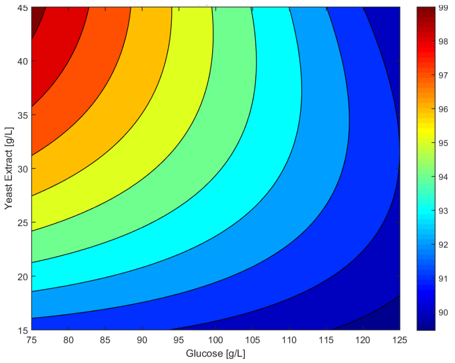


(b) pgm

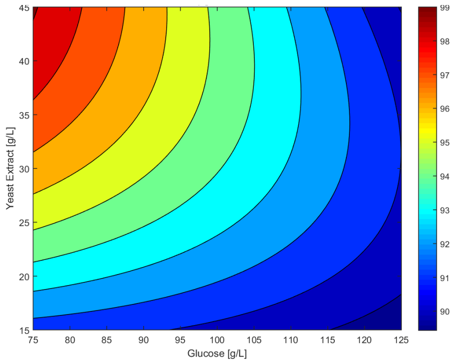


(c) eno

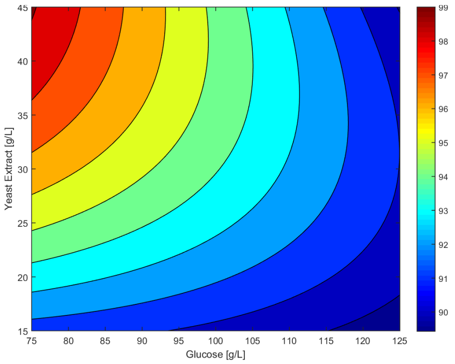


(d) pykA

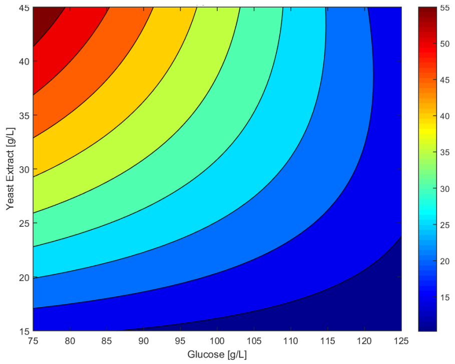


(e) ppsA

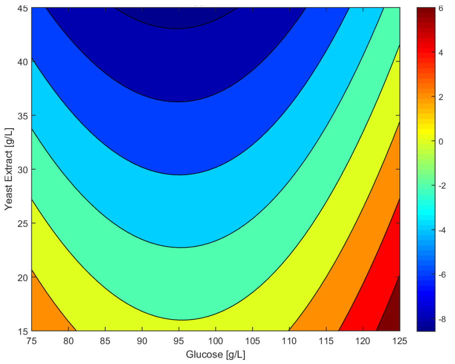


(f) lpdA

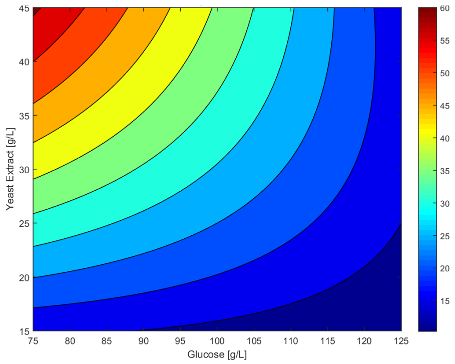


Figure 38: EMP pathway calculated flux MSContours pt.2

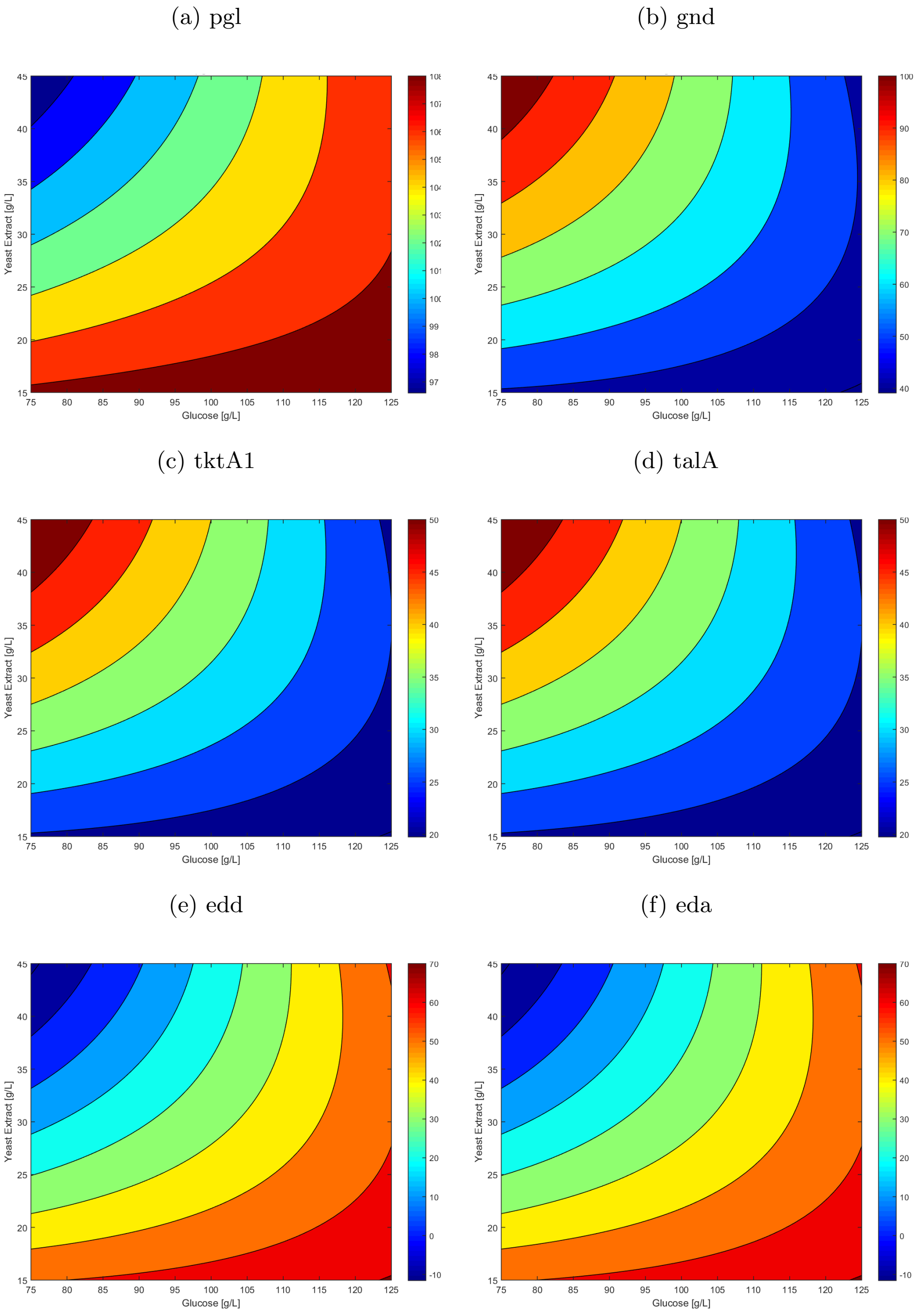


Figure 39: PPP pathway calculated flux MSContours

(a) gltA

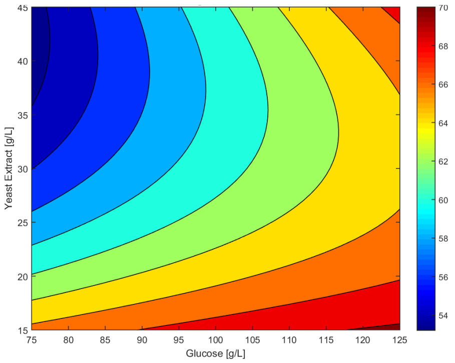


(b) acn

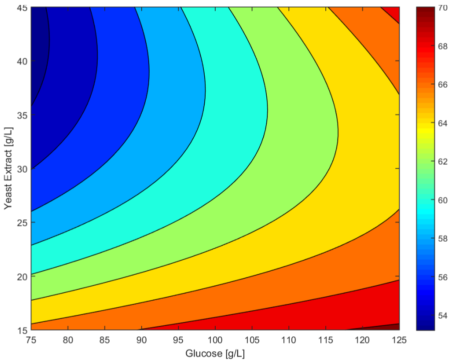


(c) icdA

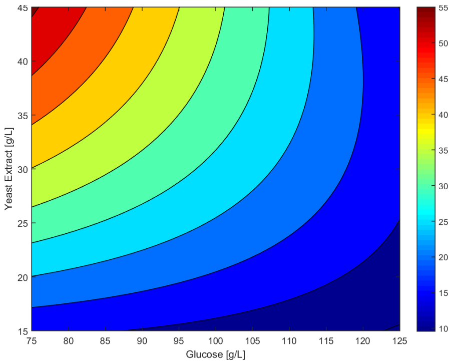


(d) sucAB

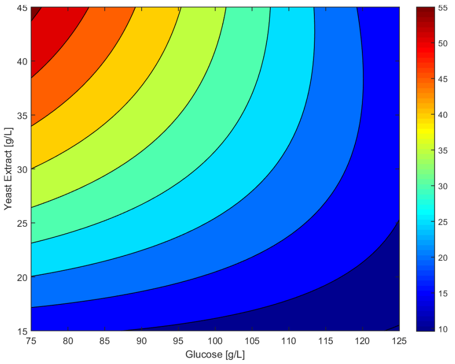


(e) sucCD

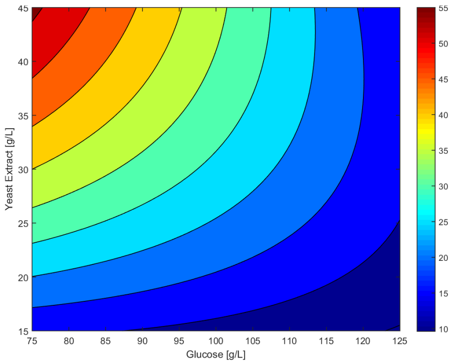


Figure 40: TCA pathway calculated flux MSContours pt.1

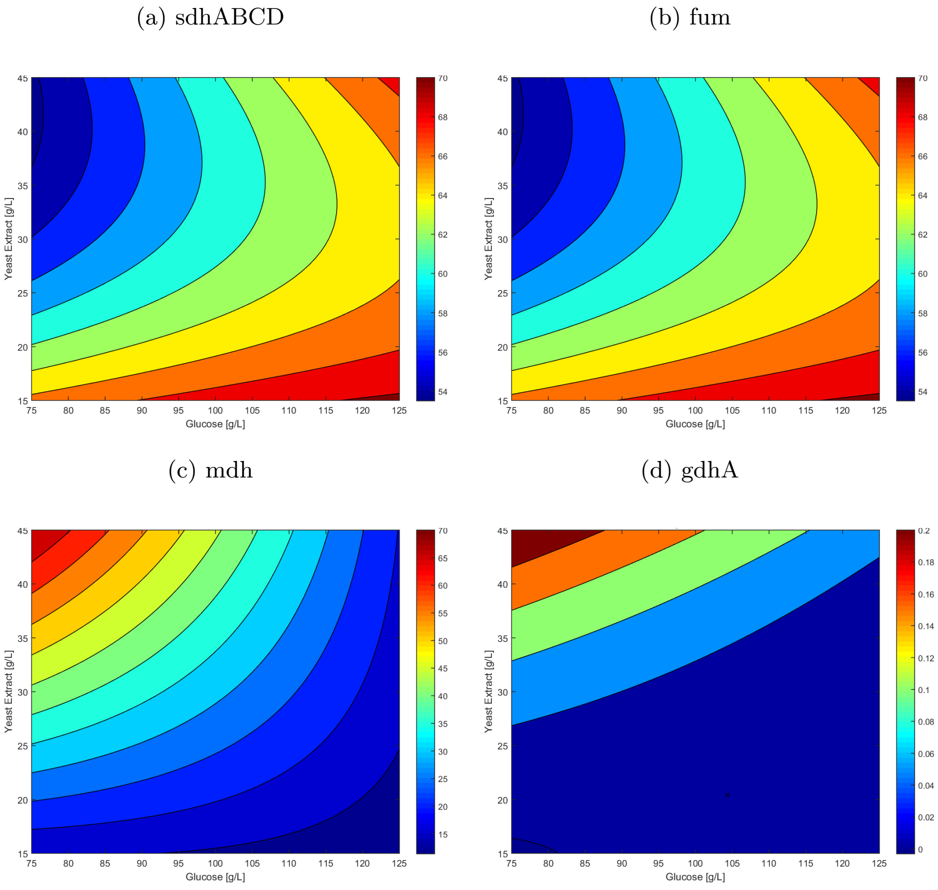


Figure 41: TCA pathway calculated flux MSContours pt.2

(a) eno

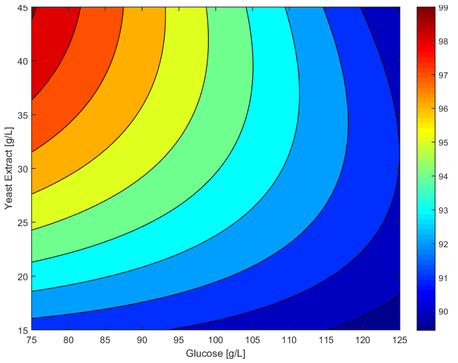


(b) ppsA

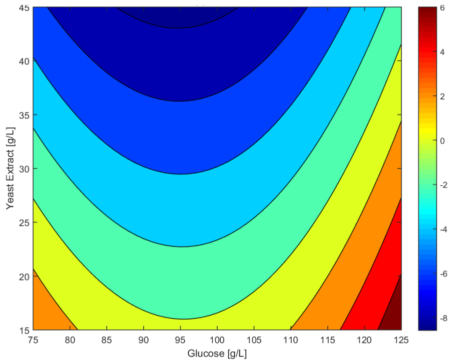


(c) pckA

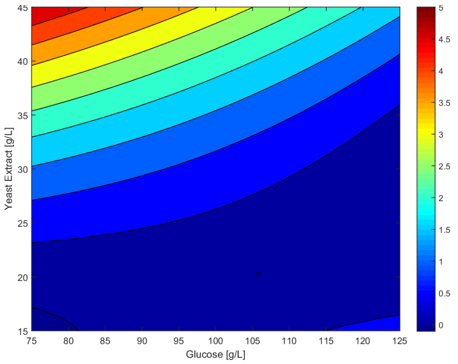


(d) pykA

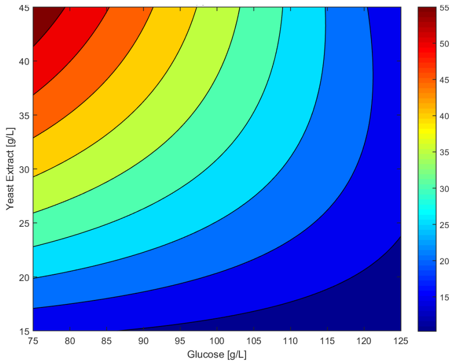


(e) ppc

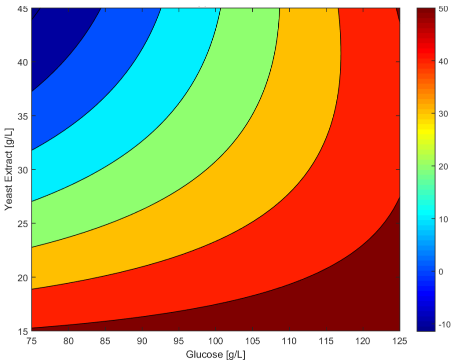


(f) aroG

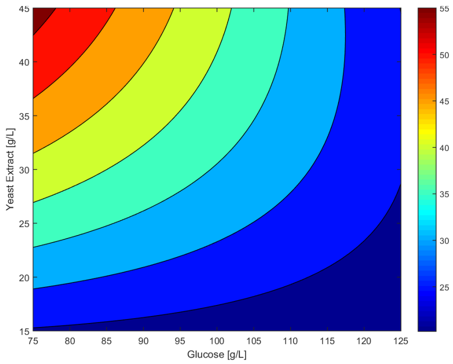


Figure 42: PEP metabolism calculated flux MSContours

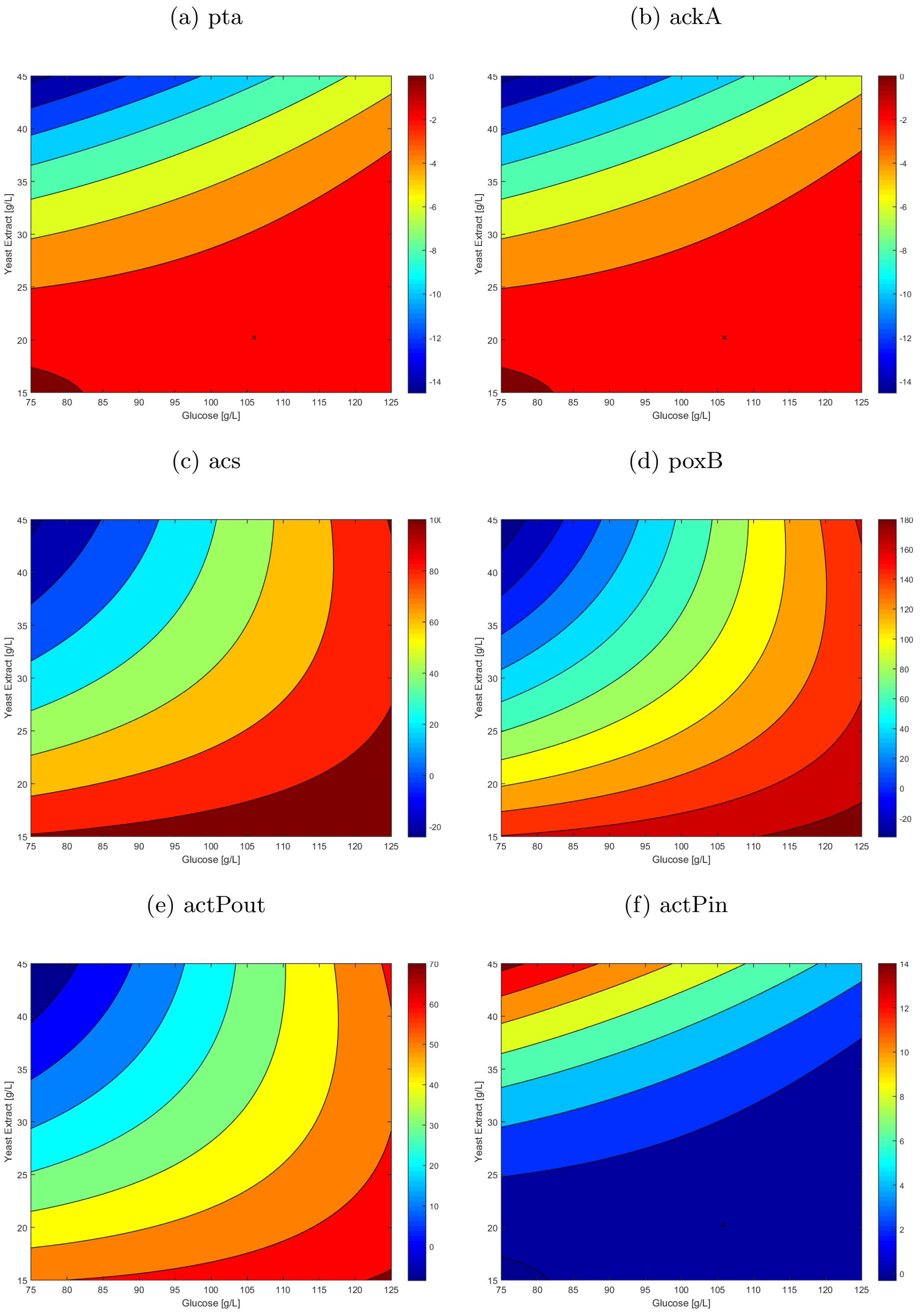


Figure 43: PYR metabolism calculated flux MSContours

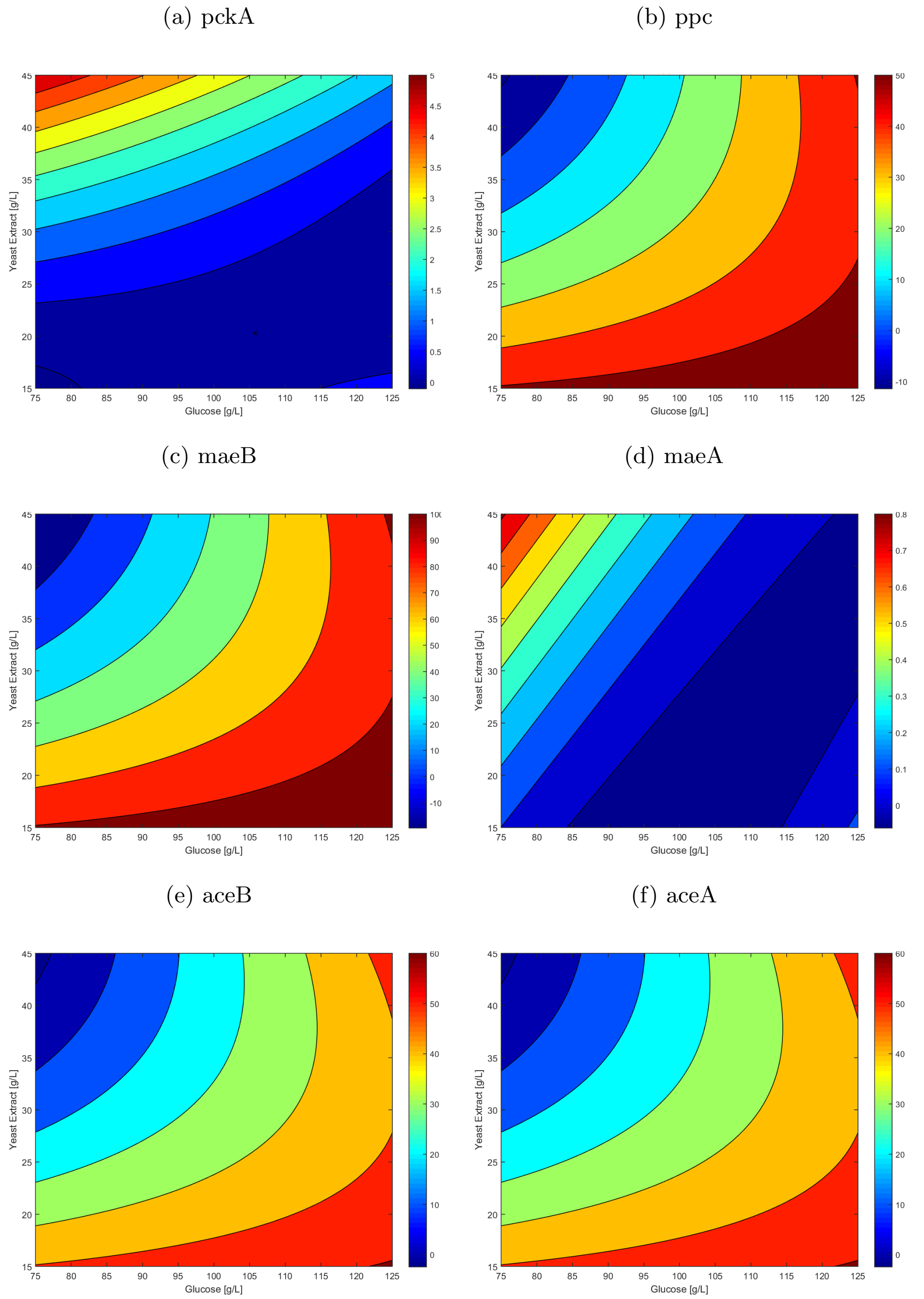


Figure 44: Anaplerotic genes calculated flux MSContours

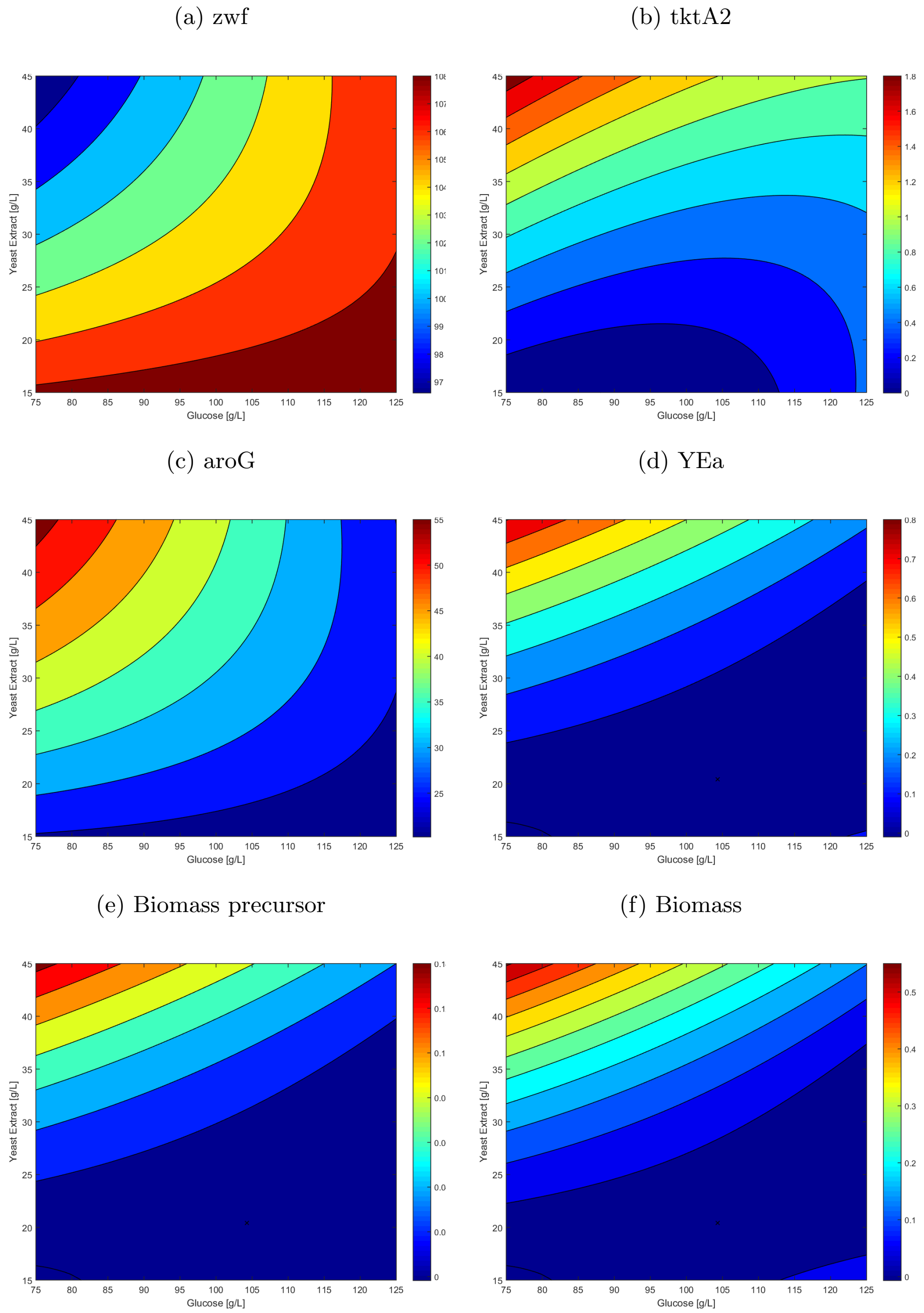


Figure 45: Syntetic operon genes calculated flux MSContours

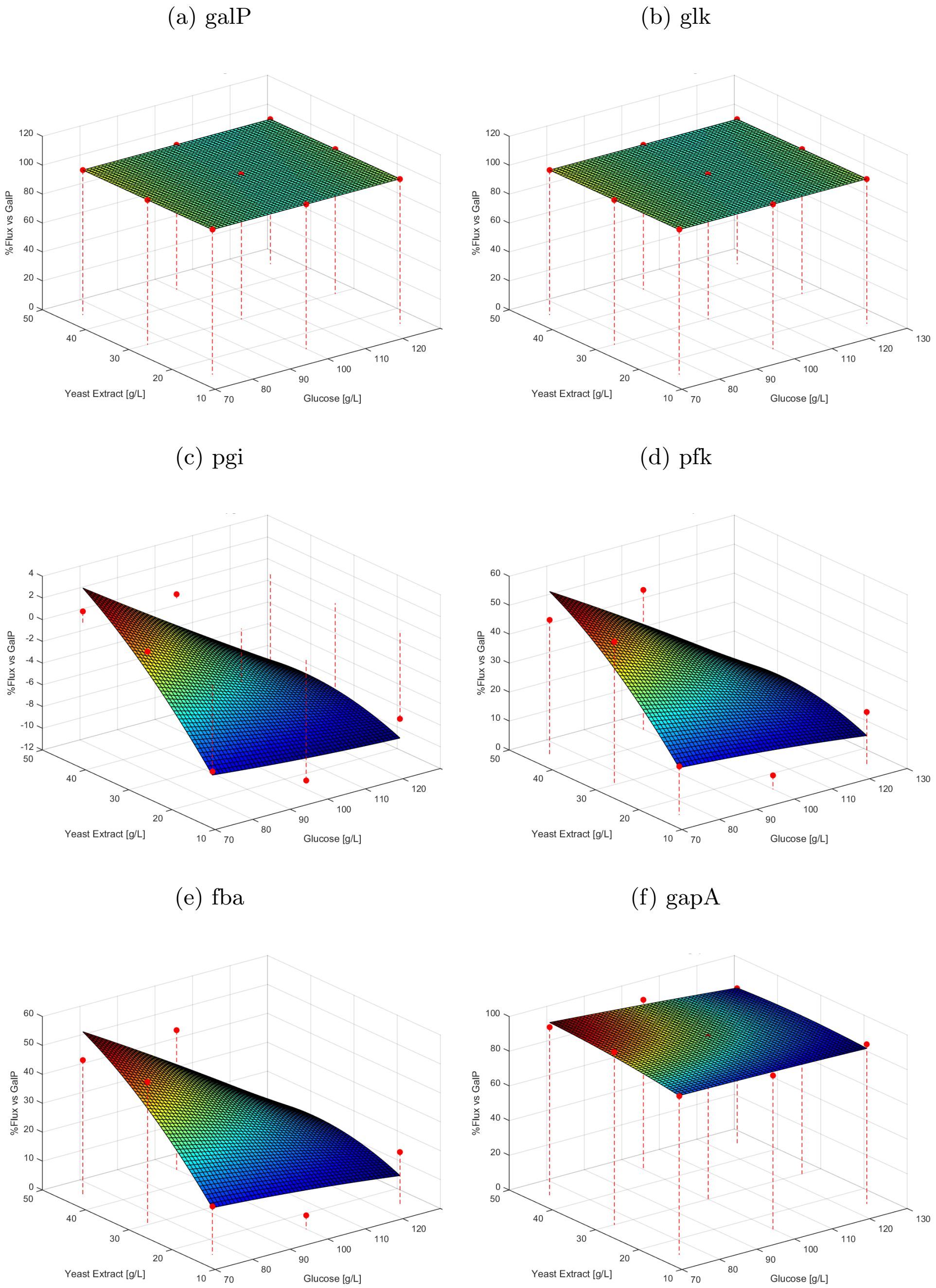


Figure 46: EMP pathway calculated flux MSSurfaces pt.1

(a) pgk

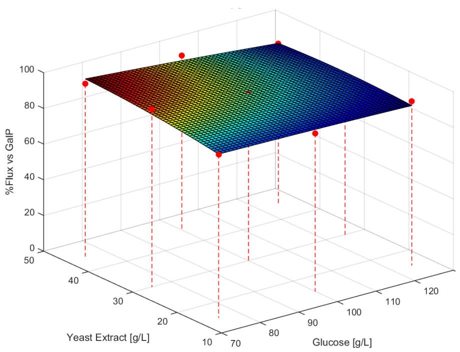


(b) pgm

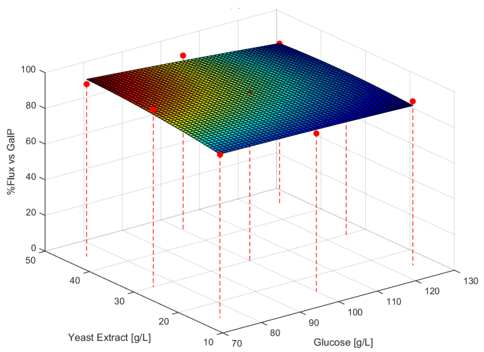


(c) eno

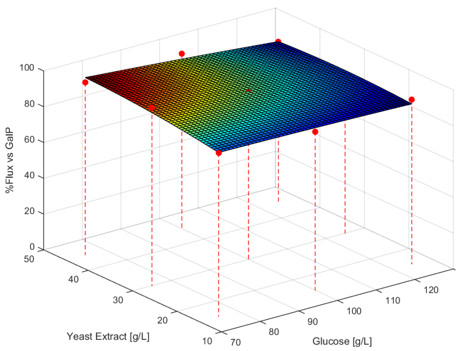


(d) pykA

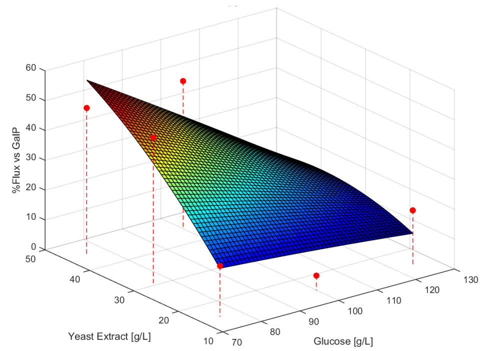


(e) ppsA

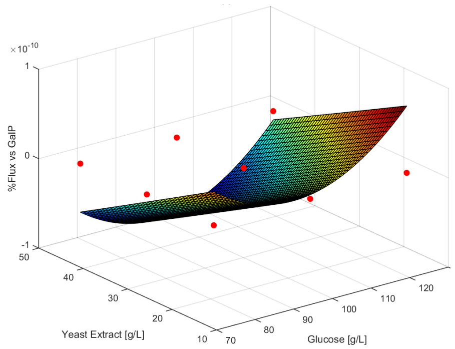


(f) lpdA

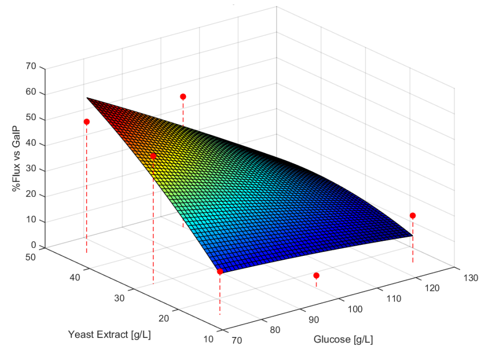


Figure 47: EMP pathway calculated flux MSSurfaces pt.2

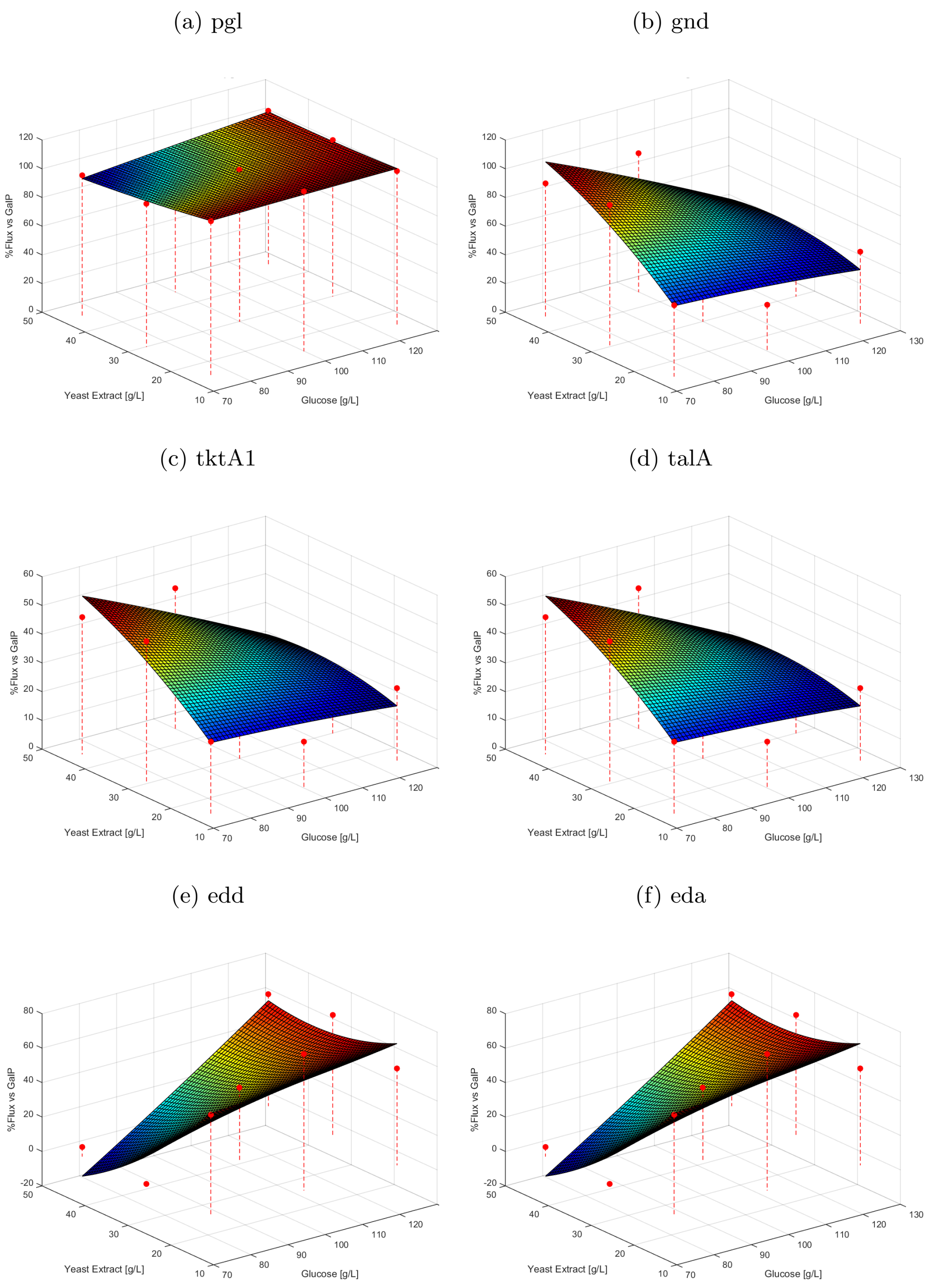


Figure 48: PPP pathway calculated flux MSSurfaces

(a) gltA

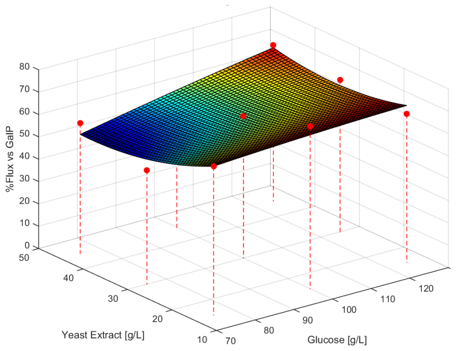


(b) acn

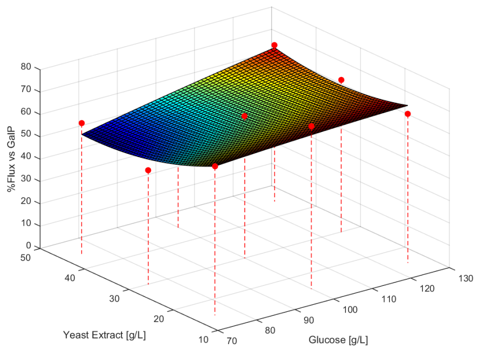


(c) icdA

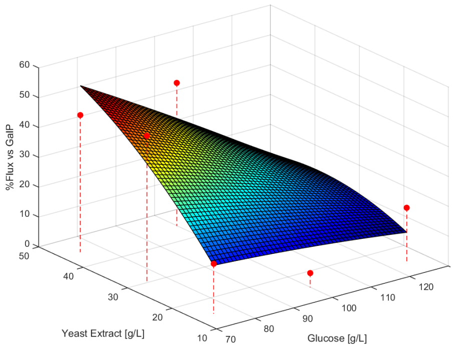


(d) sucAB

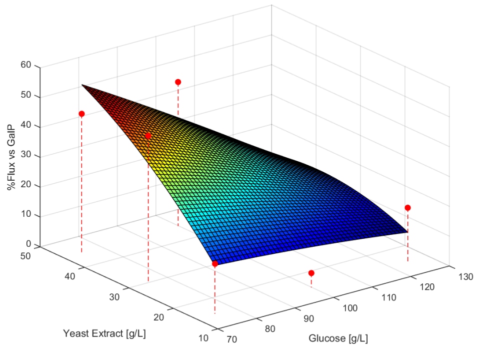


(e) sucCD

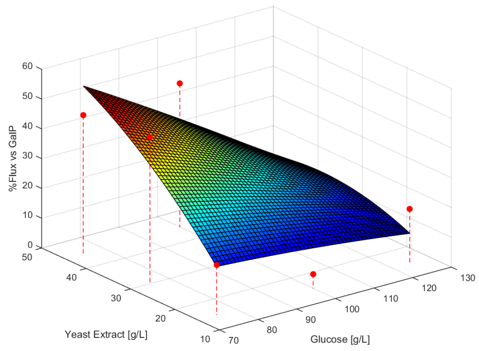


Figure 49: TCA pathway calculated flux MSSurfaces pt.1

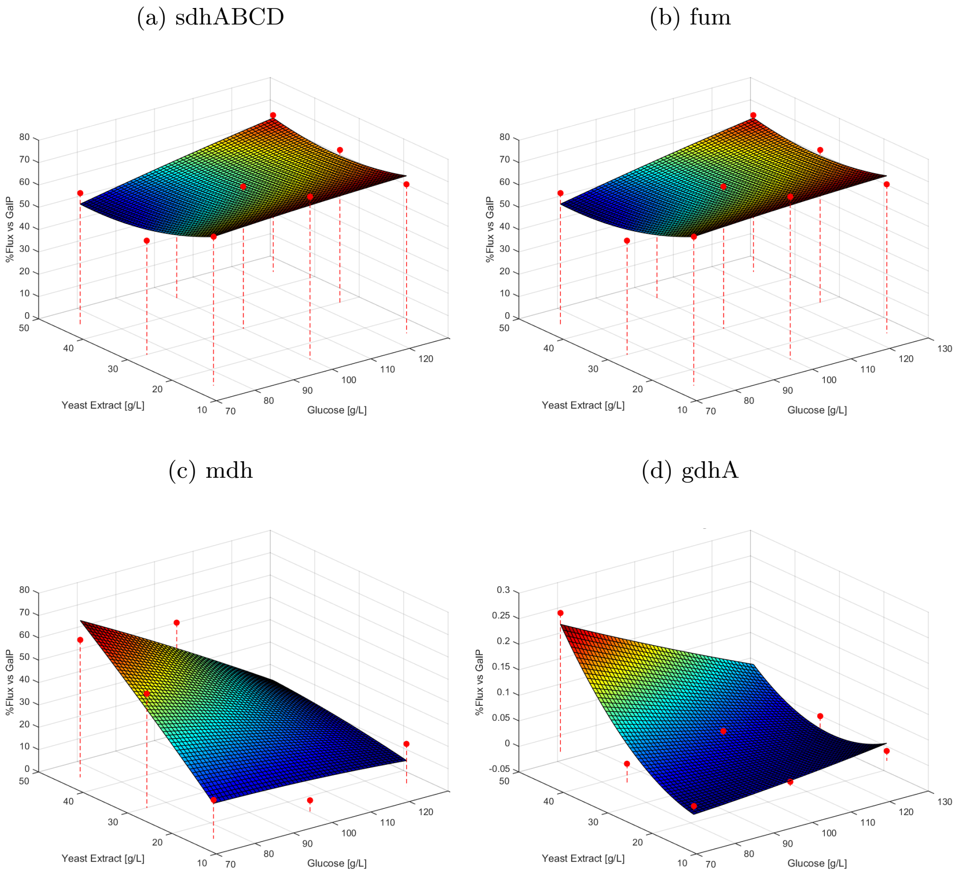


Figure 50: TCA pathway calculated flux MSSurfaces pt.2

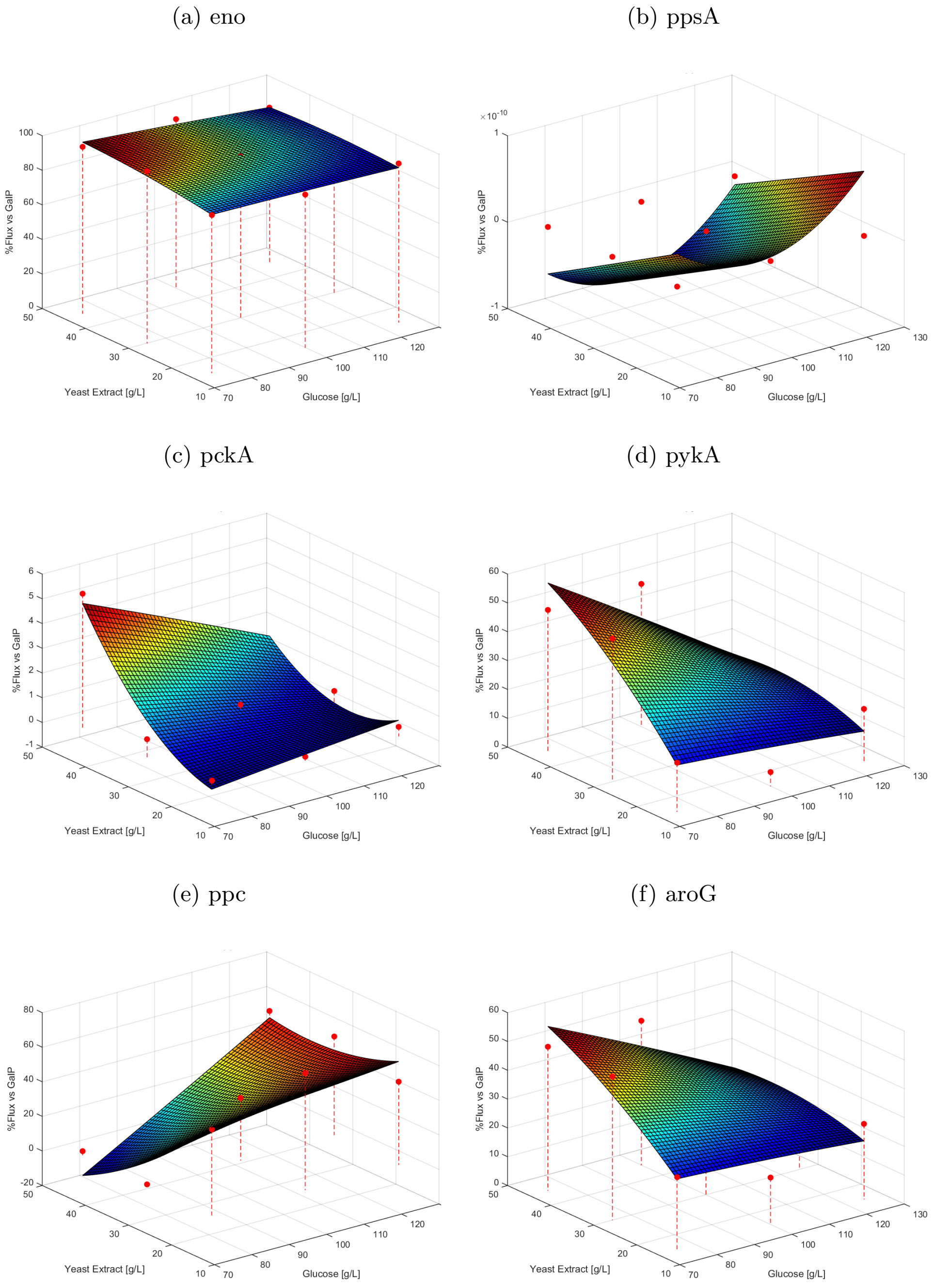


Figure 51: PEP metabolism calculated flux MSSurfaces

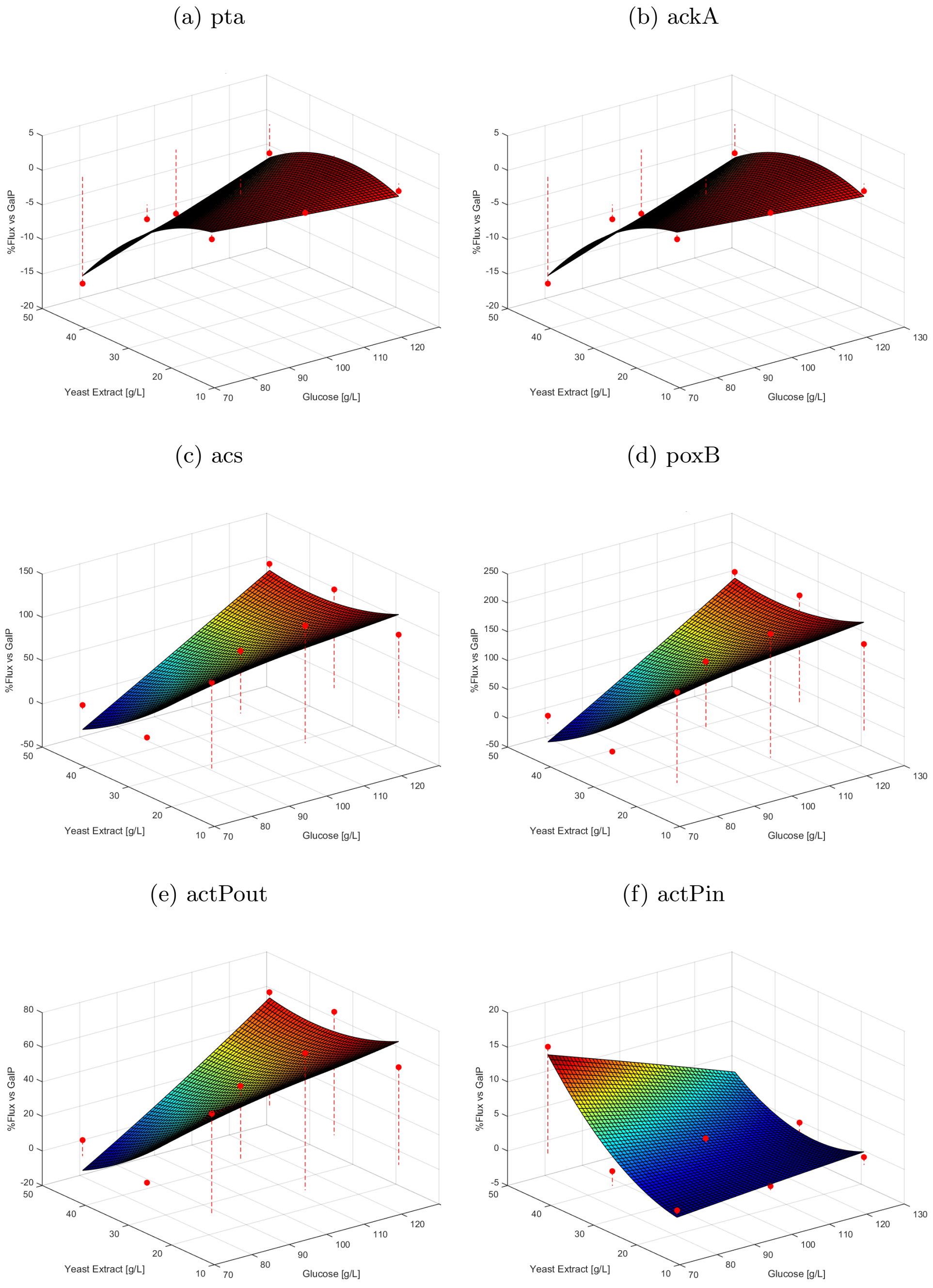


Figure 52: PYR metabolism calculated flux MSSurfaces

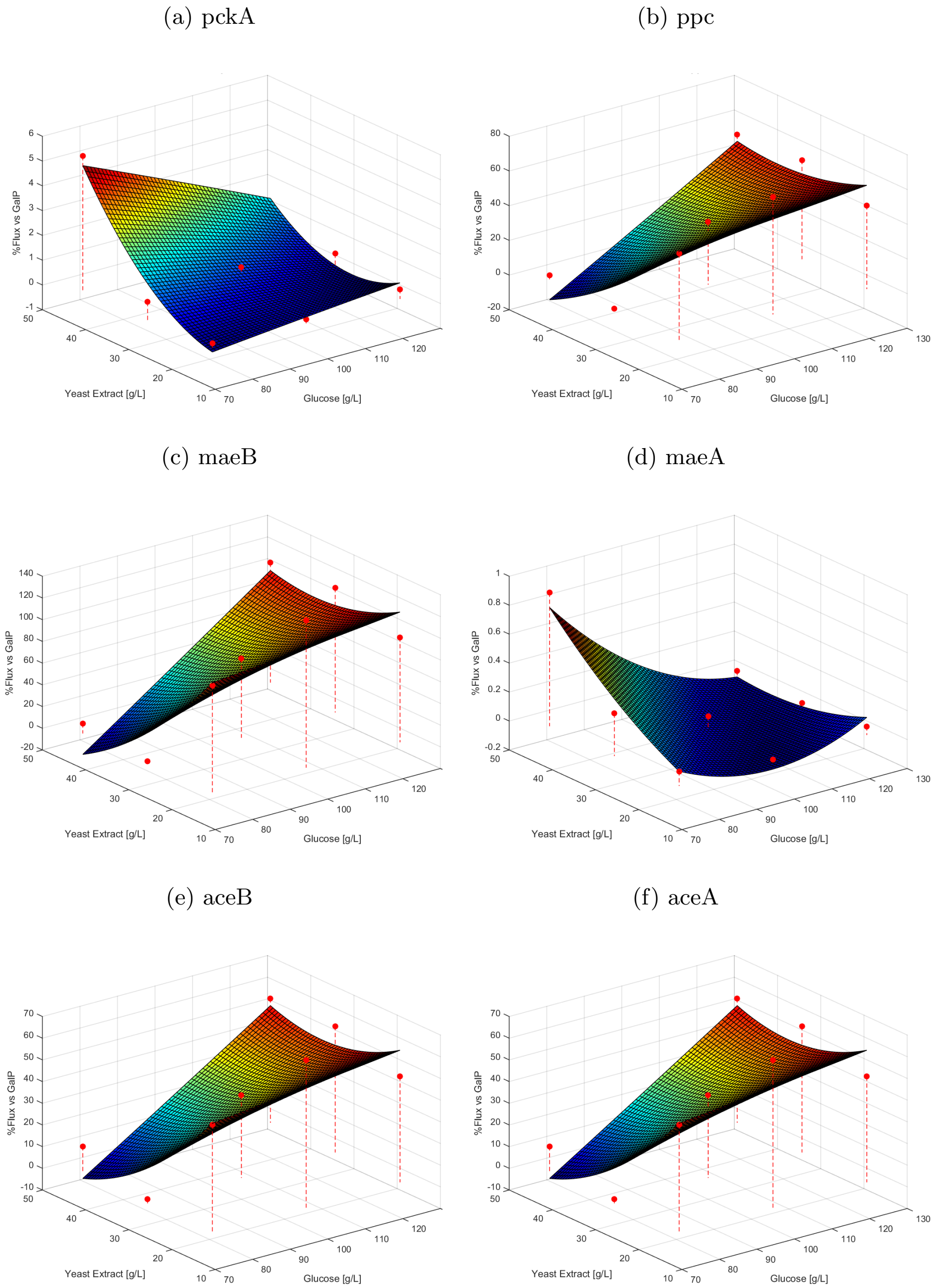


Figure 53: Anaplerotic genes calculated flux MSSurfaces

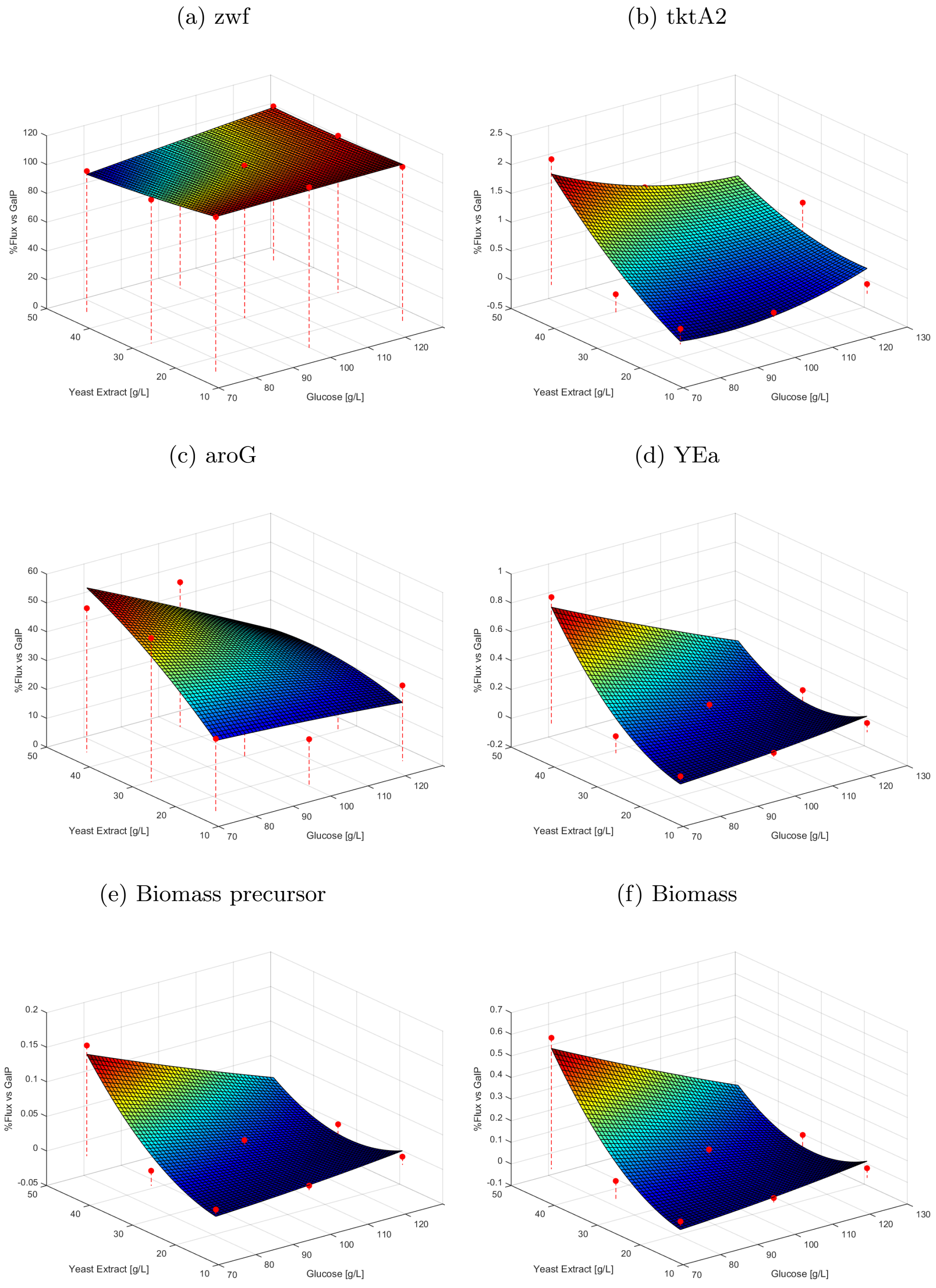


Figure 54: Syntetic operon genes calculated flux MSSurfaces
