# Supplementary material for: Metabolic modeling and response surface analysis of an Escherichia coli strain engineered for shikimic acid production
Source: BMC Syst Biol. 2018 Nov 12;12:102. doi: 10.1186/s12918-018-0632-4 (PMC6233605; doi:10.1186/s12918-018-0632-4)
Supplement: Supplementary file 4 — Strains, plasmids and oligonucleotides used on this work, AR36 poxB− figures. Description: Table for all plasmids and oligonucleotides used. AR36 ΔpoxB strain construction. Fermentation profiles for AR36 ΔpoxB and AR36 on highly concentrated substrate media. (PDF 215 kb) [file 12918_2018_632_MOESM4_ESM.pdf]

SUPPLEMENTARY MATERIAL 4

Metabolic modeling and response surface analysis for an engineered Escherichia coli for shikimic acid production

Juan A. Martínez, Alberto Rodriguez, Fabian Moreno, Noemí Flores, Alvaro R. Lara, Octavio T. Ramírez, Guillermo Gosset and Francisco Bolivar

In order to confirm the observations related to the flux carried by the product of poxB, this gene was inactivated in the strain AR36. The strain AR36ΔpoxB was constructed using the well standardized methodology reported by Datsenko et al. [1], which consist in the interruption of a sequence using homology recombination. An AR3 strain, which is the AR36 parental strain lacking the six genes operon plasmid (pTrcAro6), was transformed with the plasmid pKD46 expressing the Red recombinase system of phage λ. For chromosomal inactivation, a chloramphenicol resistance cassette (CAT) was amplified from the pKD3 plasmid, using a pair of oligonucleotides containing 50 pb homologies to the poxB flanking secuence and 21 pb homologies to CAT (table XX). The PCR product was electropored into the AR3+pKD46 strain cultivated with arabinose as inductor of the recombinase system. The electropored cells were resuspended in SOC broth and cultured during two hours at 30 $\circ$ C and then plated on LB solid media added with chloramphenicol as selecting media at 37 $\circ$C. A colony was selected and verified by PCR using external poxB oligonucleotides. A AR3ΔpoxB positive strain was then transformed with plasmid pTrcAro6 developed by Rodriguez et al. [2] to get a AR36ΔpoxB strain.

Table 1 Strains, Plasmids and oligonucleotides used

	**Strains**	
Name	Characteristics	Reference
AR3	JM101 $\Delta(ptsH, ptsI, crr) :: kanPTS^-$ Glc$^+$; laboratory-evolved strain; *lacI*$^-$ *aroK*$^-$ *aroL*$^-$ *pykF*$^-$	[2]
AR3e	AR3 + pTrc327par (plasmid vector without synthetic operon)	[2]
AR36	AR3 + pTrcAro6 (Trc/*aroB*$^+$*tktA*$^+$*aroGfbr*$^+$*aroE*$^+$*aroD*$^+$*zwf*$^+$)	[2]
	**Plasmids**	
Name	Characteristics	Reference
pKD3	PCR template for amplification of chloramphenicol resistance gene flanked by homologous sequences	[1]
pKD46	Plasmid expressing λ-Red recombinase system with thermosensitive origin of replication	[1]
pTrc327par	Contains the promoter, polylinker, and terminators of pTrc99A, and par and ori regions of pBR327par	[2]
pTrcAro6	pTrc327par containing a 6-gene synthetic operon to enhance the production of shikimate,(Trc/*aroB*$^+$*tktA*$^+$*aroGfbr*$^+$*aroE*$^+$*aroD*$^+$*zwf*$^+$)	[2]
	**Oligonucleotides**	
POXbComp Fwd	TATCG CGCCG GGCAA TATG::19bp	This work
POXbComp Rev	TTCGC GCGAG ACGTC AAGAT::20bp	This work
InactpoxB fwd	TCAGA TGAAC TAAAC TTGTT ACCGT TATCA CATTC AGGAG ATGGA GAACC GTGTA GGCTG GAGCT GCTTC G ::71bp	This work
InactpoxB rev	TCAGA TGAAC TAAAC TTGTT ACCGT TATCA CATTC AGGAG ATGGA GAACC GTGTA GGCTG GAGCT GCTTC G::71bp	This work

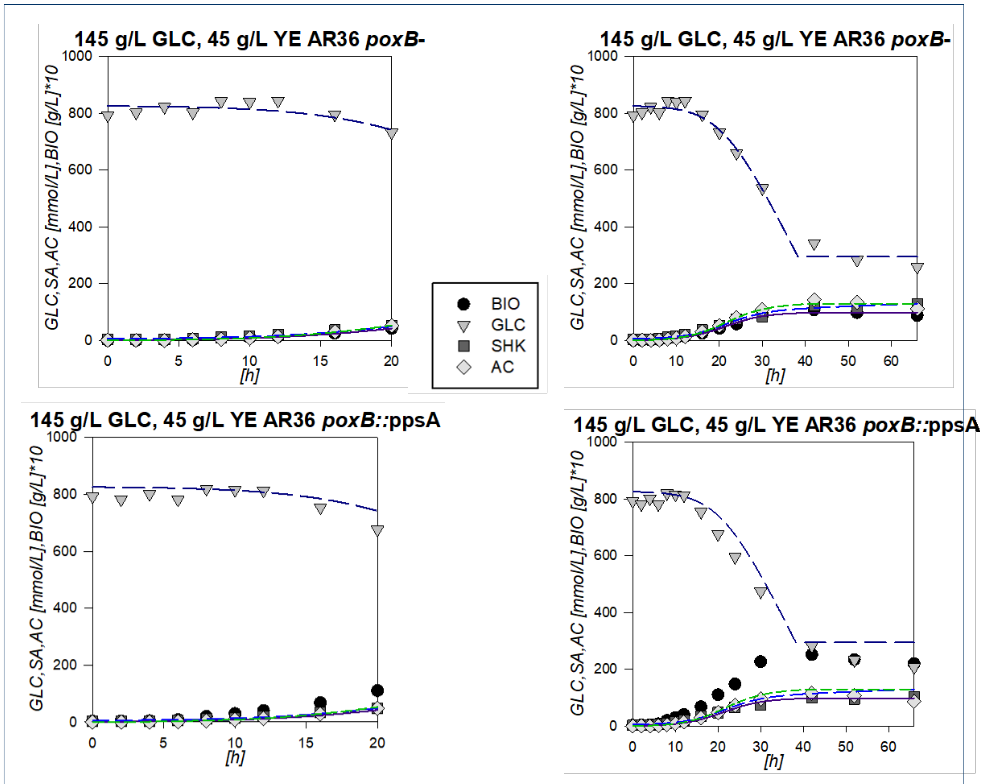

Figure 1 AR36$\Delta poxB$ strain 145:45 GLC:YE initial conditions fermentation profiles A) up to 20 hours B) to final fermentation time, and its comparison with a 145:45 GLC:YE initial conditions fermentation with a AR36 poxB::ppsA strain, C) up to 20 hours D) to final fermentation time. Lines on all graphs represent the model obtained for the AR36$\Delta poxB$ strain @ 145:45

Author details

References

1. Ka D, BL W. One-step inactivation of chromosomal genes in Escherichia coli K-12 using PCR products. Proc Natl Acad Sci U S A. 2000;6:6640–6645.
2. Rodriguez A, Martínez JA, Báez-Viveros JL, Flores N, Hernández-Chávez G, Ramírez OT, et al. Constitutive expression of selected genes from the pentose phosphate and aromatic pathways increases the shikimic acid yield in high-glucose batch cultures of an *Escherichia coli* strain lacking PTS and *pykF*. Microbial Cell Factories. 2013;12:86.
